# Maize RNA PolIV affects the expression of genes with nearby TE insertions and has a genome-wide repressive impact on transcription

**DOI:** 10.1186/s12870-017-1108-1

**Published:** 2017-10-12

**Authors:** Cristian Forestan, Silvia Farinati, Riccardo Aiese Cigliano, Alice Lunardon, Walter Sanseverino, Serena Varotto

**Affiliations:** 1 Department of Agronomy, Food, Natural resources, Animals and Environment, University of Padova, Viale dell’Università 16, 35020 Legnaro, PD Italy; 2 Sequentia Biotech SL, Calle Comte D’Urgell, 240 Barcelona, Spain; 3 Present Address: Department of Biology and Huck Institutes of the Life Sciences, Penn State University, University Park, Pennsylvania, PA 16802 USA

**Keywords:** *Zea mays*, Transcriptome analysis, RdDM, siRNAs, Transposable elements

## Abstract

**Background:**

RNA-directed DNA methylation (RdDM) is a plant-specific epigenetic process that relies on the RNA polymerase IV (Pol IV) for the production of 24 nucleotide small interfering RNAs (siRNA) that guide the cytosine methylation and silencing of genes and transposons. *Zea mays RPD1/RMR6* gene encodes the largest subunit of Pol IV and is required for normal plant development, paramutation, transcriptional repression of certain transposable elements (TEs) and transcriptional regulation of specific alleles.

**Results:**

In this study we applied a total RNA-Seq approach to compare the B73 and *rpd1/rmr6* leaf transcriptomes. Although previous studies indicated that loss of siRNAs production in RdDM mutants provokes a strong loss of CHH DNA methylation but not massive gene or TEs transcriptional activation in both Arabidopsis and maize, our total RNA-Seq analysis of *rpd1/rmr6* transcriptome reveals that loss of Pol IV activity causes a global increase in the transcribed fraction of the maize genome. Our results point to the genes with nearby TE insertions as being the most strongly affected by Pol IV-mediated gene silencing. TEs modulation of nearby gene expression is linked to alternative methylation profiles on gene flanking regions, and these profiles are strictly dependent on specific characteristics of the TE member inserted. Although Pol IV is essential for the biogenesis of siRNAs, the genes with associated siRNA loci are less affected by the *pol IV* mutation.

**Conclusions:**

This deep and integrated analysis of gene expression, TEs distribution, smallRNA targeting and DNA methylation levels, reveals that loss of Pol IV activity globally affects genome regulation, pointing at TEs as modulator of nearby gene expression and indicating the existence of multiple level epigenetic silencing mechanisms. Our results also suggest a predominant role of the Pol IV-mediated RdDM pathway in genome dominance regulation, and subgenome stability and evolution in maize.

**Electronic supplementary material:**

The online version of this article (10.1186/s12870-017-1108-1) contains supplementary material, which is available to authorized users.

## Background

Regulation of gene expression and transposable element (TE) activity is fundamental for both proper plant development and genome integrity protection over generations. In plants, 24 nucleotide small interfering RNAs (24 nt siRNAs) play a critical role in transcriptional gene silencing (TGS) of TEs, repetitive sequences, transgenes and endogenous genes, guiding the RNA-directed DNA methylation pathway (RdDM; [1–4]). These 24 nt siRNAs, integrated into ARGONAUTE (AGO) proteins (primarily AGO4, but also AGO6 and AGO9 [5, 6]), guide the cytosine methylation and histone modification of corresponding DNA sequences, leading to chromatin states that are refractive to transcription.

In the canonical RdDM pathway [7] two plant specific nuclear RNA polymerases, Pol IV and Pol V [8–11], together with a RNA-dependent RNA polymerase 2 (RDR2 [12]) are necessary for the synthesis of small and long non-coding RNAs that trigger RNA-dependent de novo DNA methylation at the homologous sequence.

Pol IV initiates the transcription of non-coding RNAs that are then converted into dsRNA by RDR2: both enzymes are essential for the biogenesis of 24 nt siRNAs and they physically associate in Arabidopsis and maize [13–15]. Pol IV transcripts are short (mostly 26-45 nt, with the peak of their size distribution occurring at approximately 30 bp) and expressed at low levels, characteristics that prevent their identification in classical RNA-Seq experiments [16, 17].

Pol V is not directly required for siRNA biogenesis at most loci [18] but generates long non-coding RNAs that act as a scaffold for base pairing to AGO-bound siRNAs [19, 20], allowing the recruitment of the de novo methyltransferase DRM2 (DOMAINS REARRANGED METHYLASE 2) and chromatin-modifying components to loci targeted for DNA methylation and TGS. RdDM guides cytosine methylation in CG, CHG and CHH sequence contexts, but CHH methylation is the best indicator of RdDM, as CG and CHG methylation can be maintained by other DNA methylation pathways (reviewed in [1]).

RdDM targets repetitive sequences such as TEs and it also affects many processes in plant development, defense and allelic crosstalk. Approximately 4000–8000 loci in Arabidopsis and 244,000 loci in maize give rise to clusters of 24 nt siRNAs [18, 21–23], and their accumulation is dramatically reduced in RdDM mutants in both species [22–25]. Mutant plants lacking Pol IV function are defective in siRNAs production [18, 22, 23, 26] and present pleiotropic defects at phenotypic level [8, 9, 27]. Arabidopsis *NUCLEAR RNA POLYMERASE D1A* (*NRPD1A*) loss of function mutants are late flowering [11], while maize *rna polymerase d1* (*rpd1*) mutants have multiple developmental defects and trans-generational degradation in plant quality compared to non-mutant siblings [27, 28]. The singular diversity on the number of siRNA loci identified in maize vs Arabidopsis and the different impacts of *rpd1/NRPD1A* mutations on phenotypes of the two species are potentially related to their different genomic TE contents (in maize they account for ~85% of the genome compared to ~10% in Arabidopsis [29–31]).

Recent works reported that loss of siRNAs production in RdDM loss of function mutants provokes a strong reduction of CHH methylation at RdDM loci. However, this loss of methylation at the so-called mCHH islands do not cause a massive TEs release, even in a TEs enriched genome such as that of maize [25, 32, 33], predicting that Pol IV-dependent cytosine methylation is not essential to maintain TE silencing. Furthermore, at genome-wide level, CHH methylation tends to be found upstream of the maize gene transcription starts and is only associated with certain types of transposons, while CG and CHG methylation is commonly found in all maize transposons but is relatively low near the start and stop of genes [34].

Being mobile elements that can move around the genome and accumulate through periodic transposition burst in the plant genome, TEs have for a long time been considered as parasitic junk DNA and at best “mortar” elements of the genome structure variation. However, when discovered by Barbara McClintock [35], TEs were first described as “controlling elements” based on their ability to influence the expression of nearby genes. They can affect gene function by interrupting gene coding sequences or altering their regulatory elements and several specific examples of TE influence on the expression of nearby genes have been reported [36–38]. TEs were further identified as candidates for involvement in plant response and adaptation to stressful environments [39].

In previous works [23, 40] we analyzed the maize stress-responsive transcriptome regulation and small RNA populations in both B73 wild type and *rpd1* Pol IV mutant (also known as *required to maintain repression 6* - *rmr6* [27]). The first re-annotation of the total maize transcriptome, including non-polyadenylated transcripts, was coupled with the identification of its non-coding portion, revealing hundreds of genes and lncRNAs differentially expressed in response to long-term stress application. Interestingly, the amplitude of the stress-modulated gene set is very different between B73 wild type and *rpd1/rmr6* mutant plants, as a result of a stress-like effect on genome regulation caused by the epiregulator mutation itself, which appears to activate many stress-related genes even in control growth condition [40]. 24 nt siRNAs are dramatically reduced in *rpd1/rmr6* mutant plants but mutant gene up-regulation was not significantly correlated with loss of flanking siRNAs [23]. Similarly, several recent studies reported transcriptome, siRNA and methylation analysis in maize RdDM mutants, highlighting that despite the dramatic reduction of 24 nt siRNA loci and the subsequent loss of mCHH island observed, a relatively low number of genes were found differentially expressed, the majority of them were up-regulated [25, 32, 33, 41–43]. These works clearly demonstrated that RdDM activity is critical for creating chromatin boundaries between silenced near-gene inserted transposon and genes themselves, but also supported the thesis that the global alterations in siRNA and methylation profiles caused by RdDM impairing had a minor impact on transposon and gene transcription [1, 44].

In this study, starting from the first total RNA-Seq analysis in maize, we thoroughly investigated the *rpd1/rmr6* mutant transcriptome to identify and characterize RdDM targets. Global analyses of the transcribed portion of the genome and expression profile comparisons of the full gene set showed an overall increase in transcription of the maize genome in *rpd1/rmr6* mutant compared to wild type. We also provided further details on misregulated genes and new insights into the role of Pol IV-mediated transcriptional regulation in maize. By extensive and integrated analysis of gene expression, TEs distribution, smallRNA targeting and DNA methylation levels, our results point to the genes with nearby TE insertions as being the most strongly affected by Pol IV-mediated gene silencing, but also indicate that although Pol IV is essential for the biogenesis of siRNAs, the genes with associated siRNA loci are the less affected by Pol IV loss of function. In addition, our findings indicate an involvement of the Pol IV-mediated RdDM pathway in genome dominance regulation, and subgenome stability and evolution.

## Results

### Quantitation of the maize genome transcription extent

The analyzed RNA-Seq dataset consists of 16 strand-specific and 16 non-directional, total transcriptome, Illumina single-end libraries obtained from the youngest wrapped leaf of B73 wild-type and *rpd1/rmr6–1* mutant plants. Maize plants were grown under normal (C), drought-stress (D), salinity-stress (S) or drought plus salinity-stresses (D + S) and samples were collected after ten days of treatment or seven days of post-treatment recovery as described in [40]. After quality trimming and rRNA contaminant filtering, the high quality reads (ranging from 23 to 54 million reads per library; Additional file 1) were mapped to the maize B73 reference genome (RefGen ZmB73 Assembly AGPv3). Multi-mapped reads (reads mapped equally well to two or more genomic positions, primarily due to paralogous genes and repetitive sequences), account for about 50–60% of our reads (Additional file 1). Since these multi-mapped reads contain important biological information, but also represent an important challenge for RNA-Seq analysis, we decided to discard those mapping on more than 10 different positions, reducing the multi-mapped reads to about the 30–40% of the mapped reads (Additional file 1). To overcome the different library sizes, the 16 filtered BAM files from each genotype were initially combined, obtaining 438,275,238 and 458,429,084 reads for B73 and *rpd1/rmr6*, respectively (corresponding to 714,514,067 and 775,422,668 alignments on the genome). Normalization was then performed by down-sampling, randomly selecting 700 million alignments for both B73 and rpd1/*rmr6* (deriving from 430 M and 410 M reads, respectively) to evaluate the transcribed fraction of genome in the two genotypes.

At a threshold of at least two RNA-Seq reads, we observed reads mapping to 10.94% and 10.32% of the genome in *rmr6* and B73, respectively. In the 2 billion bases maize genome, these percentages result in the additional transcription of about 13 million base pairs in *rpd1/rmr6* compared to B73 (226,194,451 bp expressed in *rpd1/rmr6* vs the 213,448,475 bp in B73, values that indicate a net increase of 6% of the transcribed fraction; Additional file 2A). Similar proportions were obtained exclusively considering 290 million uniquely mapped reads (unambiguously assigned to just one position in the reference) for each genotype, with 8.90% of the genome (184,118,131 bp) covered by at least two reads in *rpd1/rmr6* compared to 8.45% (174,635,415 bp) in B73 (Additional file 2B).

To exclude any bias introduced by down-sampling of the entirety set of reads (i.e. selection of different ratios of reads derived from stressed and control conditions in the two genotypes), the transcribed fraction of the genome was calculated also for each individual sample (after down-sampling at level of individual RNA-Seq libraries). The lower number of reads analyzed resulted in the missing of many low-expressed regions: for example, in control condition at T0, 140,831,603 bp and 141,845,343 bp resulted covered by at least 2 reads in B73 and *rpd1*/*rmr6* respectively, compared to the 213–226 million previously observed from the pooled reads. Despite this lower coverage, the independent analysis (considering either uniquely mapped or multi-mapped reads) confirmed the increase of the portion of genome transcribed in *rpd1*/*rmr6* compared to B73 in all the analyzed samples, regardless of the stress treatment or time-point (Additional file 3). Following this approach, the increased fraction of genome transcribed in *rpd1*/*rmr6* consists, on average of about 2 million of base pairs. When the single down-sampled libraries were pooled (to get equivalents number of reads from B73 and *rpd1*/*rmr6* from each growth conditions) the increase in genome transcription consists of about 12.8 million base pairs (considering multi-mapped reads) or 9 million base pairs (exclusively considering the uniquely mapped read; Additional file 3).

Altogether, these results indicate that the transcriptional rise in *pol IV* mutants is not restricted to repetitive sequences (the main RdDM targets) but strongly interests unique genome regions, and, being more represented by regions covered by few reads, it is likely to be caused by small increases at many loci rather than a strong activation of few genes. To identify the genes responsible for this transcriptional gain (and possibly their common features, if any, that make them Pol IV targets), we investigated the expression profile of the whole set of maize genes, comparing them between *rpd1/rmr6* and wild-type.

### Global expression variations of maize genes in *rmr6* mutant leaves

**Fig. 1:**
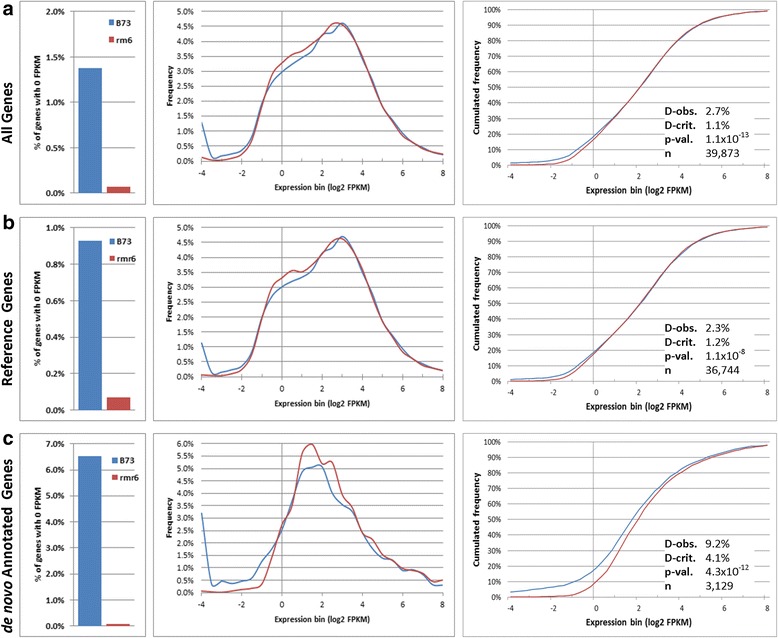
Distributions of gene expression differ between B73 and *rpd1/rmr6* mutant. Frequency of genes with 0 FPKM (left), histograms of expression distribution (center) and cumulative frequency (right) for genes with >0 FPKM are reported for all the expressed genes (**a**), and further subdivided into reference annotations (**b**) and de novo annotated loci (**c**). In all three comparisons, the frequency of genes with no expression (0 FPKM) in one genotype is significantly higher in B73 than in *rpd1/rmr6* (*P* = 0, by Fisher’s exact test) and the distributions of gene expression (including only genes with FPKM > 0) are statistically different between the two genotypes (*P* < 0.01 by Kolmogorov-Smirnov test). X-axis is log2 transformed FPKM value of gene expression (0 corresponds to FPKM =1, 2 to FPKM = 4, 4 to FPKM = 16, and so on). D-obs. represents the maximum vertical distance observed between the two curves while D-crit. is the critical distance value for the test. When both D-obs > D-stat and *p* < 0.01 the expression distributions are considered statistically different

Expression values (expressed as FPKM, fragments per kilobase per million fragments) for all the reference annotated genes (AGPv3.20) and for the de novo annotated ones (see Methods and [40]) for a detailed description of genome guided transcriptome assembly and the annotation of new maize expressed loci) were calculated and normalized between samples with Cuffquant and Cuffnorm [45] at both gene and transcript level. Different growth conditions and timepoints were considered together for the same genotype. Genes and transcripts non-expressed or expressed at too low levels in both genotypes were filtered out (see Methods; Additional files 4 and 5), and the expression distributions of expressed genes were investigated and compared between B73 and *rpd1/rmr6* as described in [46]. Among the 40,457 filtered genes, the frequency of genes with no expression in only one genotype (0 FPKM) was significantly higher in B73 than in *rmr6* (*P* = 0, by Fisher’s exact test; Fig. 1a) and the distribution of gene expression (including only genes with FPKM > 0) was statistically different between the two genotypes (*P* = 1.1 × 10^−13^, by Kolmogorov-Smirnov test; Fig. 1a). The *rpd1/rmr6* mutation appeared to cause a global increase in the basal expression of a vast number of genes, with higher frequency values ranging from 0.5 to 4 FPKM (expression bins from −1 to 2 in Fig. 1a), compared to the higher frequency of expression values lower than 0.5 FPKM observed in B73. Separating the reference annotation genes from the de novo identified ones we observed a significantly lower frequency of genes with 0 FPKM (P = 0, by Fisher’s exact test) in *rpd1/rmr6* mutant in both groups, with a more marked difference at de novo identified genes level (Fig. 1b-c). Kolmogorov-Smirnov (K-S) tests on gene expression distributions also revealed significant differences between B73 and *rpd1/rmr6* for both reference (*P* = 1.1 × 10^−7^) and de novo annotated (*P* = 4.3 × 10^−12^) genes.

These comparisons showed that the frequency of genes not expressed or with low expression values (FPKM <1) is higher in B73 than in *rpd1/rmr6*, while greater expression frequencies at higher expression values characterize the mutant, confirming the gene expression rise caused by loss of Pol IV activity. This transcriptional increase, being not exclusively caused by or restricted to de novo annotated loci, appeared to involve a large part of the maize genes.

### Pol IV controls the basal expression of non-coding TE-related transcripts

**Fig. 2:**
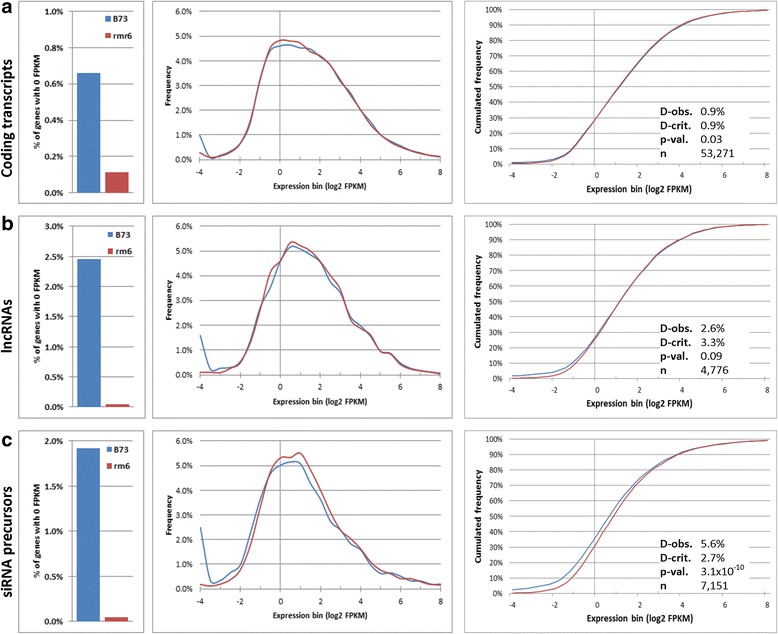
Distributions of non-coding smallRNA precursor’s expression differ between B73 and *rpd1/rmr6* mutant. Frequency of transcripts with 0 FPKM (left), histograms of expression distribution (center) and cumulative frequency (right) for transcripts with >0 FPKM are reported for the expressed coding transcripts (**a**), lncRNAs (**b**) and non-coding smallRNA (sRNA) precursors (**c**). In all three transcript groups, the frequency of transcripts with no expression (0 FPKM) in one genotype is significantly higher in B73 than in *rpd1/rmr6* (P = 0, by Fisher’s exact test) while the distributions of transcript expression (including only transcripts with FPKM > 0) are statistically different between the two genotypes exclusively for non-coding sRNA precursors (P < 0.01 by K-S test)

To identify the subgroup of genes directly responsible for the *rpd1/rmr6* overall transcriptional increase, we thoroughly analyzed the expression profiles of specific gene subgroups (sub-divided based on discriminating characteristics) in *rpd1/rmr6* compared to B73 wild-type. We initially focused on the coding potential as distinctive feature, investigating the expression profiles of coding, non-coding and TE-related transcripts. The whole set of maize transcripts was previously classified as protein-coding and non-coding transcripts (that are longer than 200 bp and do not encode proteins, or if an Open Reading Frame is present, this should be shorter than 120 amino acid and the predicted protein must not match any protein in public database). The set of non-coding transcripts was further sub-divided into non-coding smallRNA precursors (if they had a match against the known sequences of maize smallRNAs) and long non-coding RNAs [40]. The 66,153 expression filtered transcripts (see Methods; Additional files 4 and 5) were therefore sub-divived in protein-coding (53,654 transcripts), long non-coding RNAs (lncRNAs, 4898 transcripts) and non-coding smallRNA precursors (sRNA precursors, 7292 transcripts). For these three classes, the frequency of transcripts with no expression (0 FPKM) was significantly higher in B73 than in *rmr6* (*P* = 0, by Fisher’s exact test; Fig. 2) and the percentage of B73 zero expression transcripts was higher in ncRNA classes (2.45–1.92%) than for coding ones (0.66%), while in *rpd1/rmr6* the trend was opposite (0.11% of coding transcripts versus 0.04% of nc-classes). Surprisingly, the distribution profiles of transcript expression significantly diverged between the two genotypes exclusively for the non-coding sRNA precursors that were globally more expressed in *rpd1/rmr6* mutant than in B73 (*P* = 3.1 × 10^−10^, by K-S test; Fig. 2c).

**Fig. 3:**
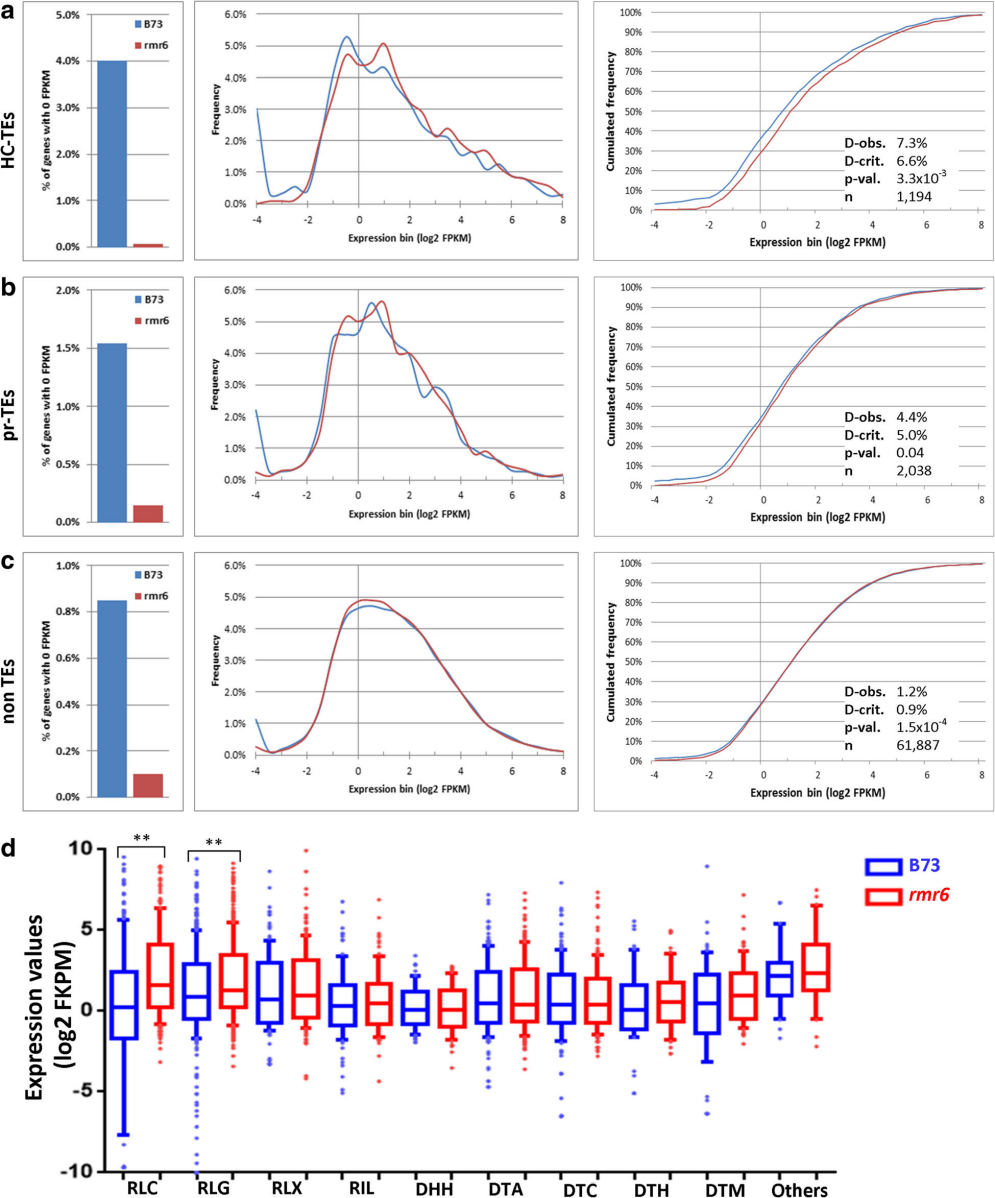
Distributions of TE-related but also non-TE transcript expression differ between B73 and *rpd1/rmr6* mutant. Frequency of transcripts with 0 FPKM (left), histograms of expression distribution (center) and cumulative frequency (right) for transcripts with >0 FPKM are reported for the previously classified high-confident-TEs (HC-TEs; **a**), putative/relics-TEs (pr-TEs; **b**) and non-TE transcripts (**c**). For all three groups, the frequency of transcripts with no expression (0 FPKM) in one genotype is significantly higher in B73 than in *rpd1/rmr6* (P = 0, by Fisher’s exact test). The distributions of transcript expression are statistically different between the two genotypes for HC-TEs and non-TE transcripts (P < 0.01 by K-S test). Expression value plots for HC-TE transcripts subdivided in super-families (**d**) indicate higher expression in *rpd1/rmr6* than B73 for RLC-Copia and RLG-Gypsy related transcripts (**: p < 0.01; Mann-Whitney U), while remaining super-families show comparable expression levels in the two genotypes. The boxes indicate the first quartile (bottom range) and third quartile (upper line) and are intersected by a crossbar corresponding to the median of the dataset. The whiskers represent the data that are within the 10th - 90th percentile and single dots depict the outliers

We then profiled the expression behavior of TE-related transcripts identified by a Blast-search approach against the Maize TE database and classified them as high-confident-TEs (HC-TEs; 9766 transcripts of which 1245 passed the expression filter) or putative/relics-TEs (pr-TEs; 9013 transcripts, 2073 expressed; see Methods). The percentage of TE-related transcripts with 0 FPKM expression was higher in B73 than in *rmr6* (*P* = 0, by Fisher’s exact test; Fig. 3a-c) and the silenced fraction was directly correlated with their TE likeness (4.02% of HC-TEs, 1.54% of pr-TEs, 0.85% of no-TEs transcripts). Similarly, their overall expression distributions were significantly but not dramatically different between the two genotypes exclusively for HC-TEs (*P* = 3.3 × 10^−3^ by K-S test; Fig. 3). Among HC-TE transcripts, those corresponding to Copia (RLC) and Gypsy (RLG) LTR elements are significantly highly expressed in *rpd1/rmr6* compared to B73, while expression profiles of transcripts matching other TE superfamilies (RLX and RIL for class I retroelements and DHH, DTA, DTC, DTH, and DTM for class II DNA TEs) showed comparable expression profiles between the two genotypes (Fig. 3d, Additional file 6). This evidence seems to point to TE-related transcripts as important targets of Pol-IV-mediated gene silencing and also confirms the diverse transcriptional regulation of specific TE subfamilies in RdDM mutants [41, 43, 47].

### TEs located in gene boundaries are responsible for the mutant transcriptional boost

Although TE-related transcripts appear to be targets of Pol IV-mediated gene silencing, we argue that these thousand transcripts cannot explain the overall transcriptional increase observed in the *rmr6* mutant leaves, also given that transcriptional boost also globally affects non-TE transcripts (*P* = 1.5 × 10^−4^ by K-S test; Fig. 3c). Considering that 66% of maize genes are located within 1 Kb of an annotated transposon [30, 39], TEs may drive the genome-wide expression variation observed in the *rmr6* mutant.

To test this hypothesis, we considered TE insertions as distinctive features, identifying genes with TEs inserted within 1 Kb upstream of the transcription start site (−1Kb TSS), in the gene body, or within 1 Kb downstream of the transcription termination site (+1Kb TTS; see Additional file 7 for a schematic representation of how genes were classified with respect to TEs) and analyzing their expression profiles in B73 and *rpd1/rmr6* mutants. As predictable, for all three gene classes, the percentage of genes with 0 FPKM expression was higher in B73 (1.52% upstream, 1.53% downstream and 1.06% inside) than in *rpd1/rmr6* (0.08%, 0.08% and 0.04% respectively). In addition, the distribution of gene expression was statistically different between the two genotypes (Additional file 8A-C), particularly for genes with TEs inserted upstream of the TSS (*P* = 3.3 × 10^−11^, by K-S test) or downstream of the TTS (*P* = 1.0 × 10^−11^) compared to genes with TE insertion within the gene body (*P* = 8.6 × 10^−9^). Histograms of expression distributions and cumulative frequencies showed higher expression of genes with nearby TE insertions (and to a lesser extent with TE inside gene bodies) for expression values ranging from 0.5 to 10 FPKM (expression bins −1 to 3 in Additional file 8A-C) in *rpd1/rmr6* mutant compared to B73. On the contrary, genes without TE insertions (inside or nearby) showed similar distribution of gene expression between genotypes (*P* = 0.08, by K-S test; Additional file 8D). Furthermore, the average gene expression level and the *rpd1/rmr6* silencing effect on TE- neighboring genes was directly correlated with the distance to the nearest TE (Additional file 8E). Genes with proximal TEs (both up- or downstream) were on average expressed at lower levels in B73 than those without proximal TEs and *pol IV* loss of function resulted in their higher expression (Additional file 8E).

**Fig. 4:**
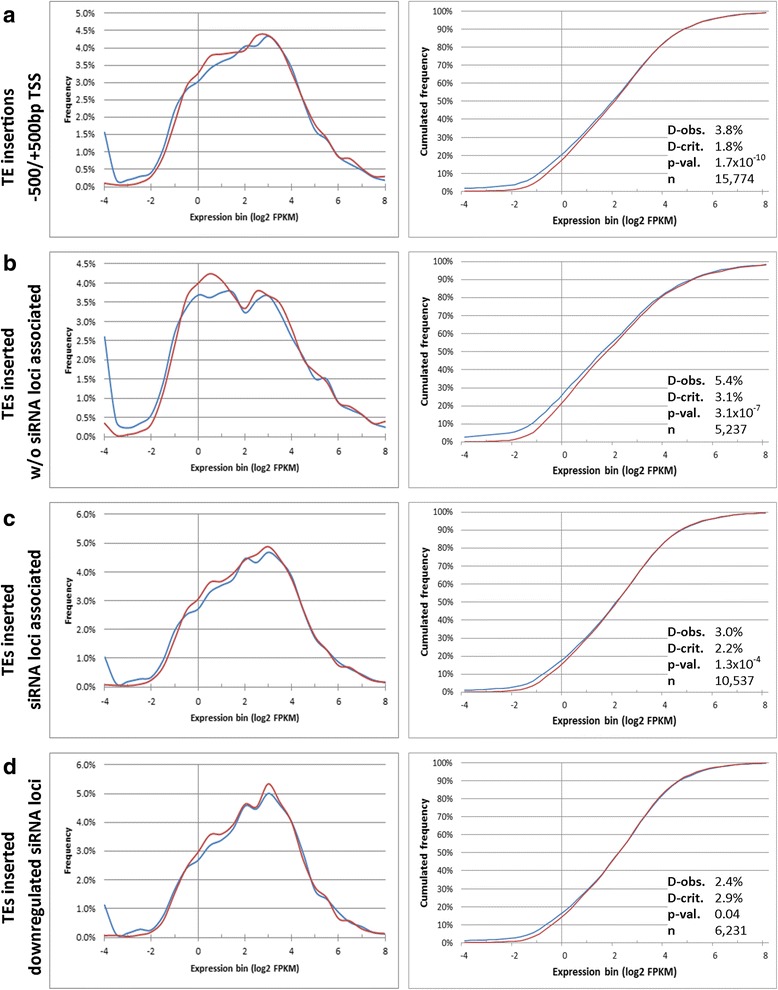
TE insertions spanning gene TSS affect expression of neighboring genes in *rpd1/rmr6* mutant, independently of siRNAs loci. Histograms of expression distribution (left) and cumulative frequency (right) for genes with >0 FPKM, reveal the high variance in expression distributions between *rpd1/rmr6* and B73 for genes with TE insertions spanning the TSS (−500/+500 bp TSS; **a**; P < 0.01 by K-S test). These genes present the maximum distance between the two genotypes’ cumulated frequency (3.8%) compared to genes with TEs inserted within 1 Kb upstream of the transcription start site (−1Kb TSS), 1 Kb downstream of the transcription termination site (+1Kb TTS) or in the gene body (see Additional file 8). Genes with nearby TEs insertion (−500/+500 bp TSS) were further subdivided in: without associated siRNA loci (**b**), with siRNA loci associated in their upstream region (−1Kb TSS; **c**) and with siRNA loci previously identified as down-regulated in *rpd1/rmr6* mutant (**d**). The distributions of gene expression are strongly statistically different between the two genotypes (D-obs = 5.4%; P < 0.01 by K-S test) for genes without siRNA-associated loci (**b**) and, to a lesser extent (D-obs = 3%) for genes with associated siRNA loci (**c**). The genes with an siRNA-associated locus down-regulated in the *rpd1/rmr6* mutant show instead non dissimilar profiles between the two genotypes (*P* = 0.04 by K-S test)

The analysis of TE’s effect on gene expression was improved by scanning the gene list coordinates considered for TE insertion using 1 Kb sliding windows, redefining an interval of maximum TE effect ranging from -500 bp to +500 bp of the TSS. This gene set, with TE insertions spanning the TSS (see schematic representation on Additional file 7), showed the higher percentage of 0 FPKM expression in B73 (1.9%) compared to *rmr6* (0.11%; P = 0 by Fisher’s exact test). Also the distributions of their expression profiles were strongly statistically different (*P* = 1.7 × 10^−10^, by K-S test; Fig. 4a), with a maximum distance between the two genotype’s cumulated frequency of 3.8% (the corresponding values were 3.4%, 3.5% and 3.3% for genes with insertion upstream of the TSS, downstream of the TTS or inside the gene body, respectively). The influence of TEs spanning the TSS on inserted gene’s expression is very marked: subtracting these loci from those with insertion upstream of the TSS or inside the gene body resulted in the flattening of expression profile differences of both these gene groups and the loss of any statistical difference between genotypes (data not shown).

All together, these results point to TEs located nearby genes (in particular in the interval − 500/+500 bp with respect to the TSS) as the main regulators of Pol IV-mediated gene silencing release in *rmr6* mutant.

### TE-mediated gene expression changes are not directly linked to the loss of siRNAs in *rmr6* mutants

Pol IV is needed to produce 24 nt siRNAs that enter the RdDM pathway triggering DNA methylation and transcriptional silencing at homologous DNA sequences (reviewed in [1]). Our research group recently profiled siRNA abundances in B73 and *rpd1/rmr6* leaves, identifying about 244,000 genomic loci producing 23-24 nt siRNAs (preferentially located in gene flanking regions), a quarter of which was significantly down-regulated in *rpd1/rmr6* compared to B73. Anyway, this loss of siRNAs caused by the *rpd1/rmr6* mutation was not being generally predictive of expression induction of the 809 genes up-regulated in the mutant [23].

To further address the effect of *pol IV* loss of function on siRNA production and TE-controlled gene regulation, we intersected the genomic coordinates of 24 nt siRNA loci previously identified, with those of the 16,091 genes with TE insertions spanning the TSS (−500/+500 bp TSS) and compared the expression distribution of different subgroups. The 5425 genes without siRNA-associated loci (within 1 Kb of the TSS) presented strongly different expression profiles between *rm6* and B73 (*P* = 3.1 × 10^−7^ by K-S test; Fig. 4b). In this gene set the expression frequency is higher in *rpd1/rmr6* than in B73 for values ranging from 1 to 8 FPKM (expression bins 0 to 3), with a maximum distance between the two genotype’s cumulated frequency of 5.4%, while for the 10,666 remaining genes associated with at least one siRNA locus, the expression profiles are less divergent (maximum distance 3.0%; *P* = 1.3 × 10^−4^; Fig. 4c). Unexpectedly, the 6289 genes with a siRNA-associated locus, which was previously found significantly down-regulated in the *rpd1/rmr6* mutant [23] showed non dissimilar profiles between the two genotypes (maximum distance 2.4%; *P* = 0.04 by K-S test; Fig. 4d).

The same results were obtained profiling the 21,447 genes with TE insertions within 1 Kb upstream of the TSS and the 21,333 genes with +1Kb TTS associated TE insertions, of which 14,952 and 13,215 have a siRNA locus associated (8699 and 6111 with down-regulated siRNA loci), respectively (data not shown).

Although Pol IV is required for siRNAs biogenesis, these results indicated that genes with associated siRNA loci are less affected by Pol IV loss of function, than those without them, confirming also at genome-wide level that loss of siRNAs is not directly associated to transcriptional activation at those loci.

### Identification of specific genes misregulated in *rmr6–1* mutant leaves

Having ranked the nearby TE insertions as the predominant feature associated to Pol IV-mediated silencing of gene expression, we performed differential expression analysis to gain further insights into Pol IV targets. Unlike the expression profile comparisons previously applied, differential expression analysis are biased toward the identification of highly expressed genes; in other words, in standard transcriptome analysis, the probability that highly expressed genes would be detected as differentially expressed is greater than that for low-count genes, that mostly account for *rpd1/rmr6* transcriptional rise (reviewed in [48]). Anyway, this differential expression analysis approach has the advantage of restricting the number of targets, specifically focusing on the predominant and statistically significant ones. Given the large number of samples analyzed (4 growth conditions, two timepoints for each genotype), we performed three independent pairwise comparisons grouping the sequenced samples in different combinations: i) Control-test-set (B73:C_T0 + B73:C_T7 vs *rmr6*:C_T0 + *rmr6*:C_T7); ii) Stress-test-set (B73:D_T0 + B73:S_T0 + B73:S + D_T0 vs *rmr6*:D_T0 + *rmr6*:S_T0 + *rmr6*:S + D_T0); iii) All-test-set (all B73 samples vs all *rmr6* samples). In addition to the typical TopHat/Cuffdiff pipeline [49], this last set was also analyzed with RSEM software tools [50] that use an alternative strategy to effectively handle ambiguous/multiple-mapping reads, accurately estimating gene-level abundance starting from large numbers of short single-end reads. After RSEM quantification, differential expression was calculated with EBSeq [51]. RSEM + EBSeq identified a larger number of differentially expressed genes (log_2_FC > |2|, corresponding to 4-fold transcriptional variation, and FDR < 0.05) than the more conservative Cuffdiff (Table 1).

**Table 1:** Summary of differential expression analyses results

	All-test-set	All-test-set - RSEM + EBSeq	Stress-test-set	Control-test-set	Shared
*rpd1/rmr6* Up-regulated	1415	3573	964	936	880
*rpd1/rmr6* Down-regulated	202	393	148	148	71

Number of genes resulting as differentially expressed (log2FC > |2|; FDR < 0.05) in each pairwise comparison, and shared in at least three independent comparisons

The larger number of genes up-regulated in all four analyses (7–9 times more than silenced ones) confirmed that RPD1/RMR6 negatively regulated gene expression. A diagnostic phenotype of *rpd1/rmr6–1* homozygous individuals is the release of epigenetic silencing of the *b1* (*booster1*) gene, which encodes a basic helix-loop-helix protein that is a transcriptional activator of anthocyanin biosynthetic enzymes [52]. One hallmark of *pol IV* loss of function is therefore the upregulation of *b1,* which was found as highly up-regulated in all the differential expression analyses performed (Additional file 9).

The genes differentially expressed in at least three of the four independent expression analyses were selected for further investigation as they likely represent RPD1/RMR6 specific targets. Genes located in a region of 30 Mb surrounding the *RMR6–1* locus (21 up-regulated and 12 down-regulated) were excluded from downstream analysis: their misregulation could be due to the sequence polymorphisms (increasing gene expression or impairing read mapping) between the introgressed *rpd1/rmr6* mutant and the B73 [41]. The resulting 880 loci commonly de-repressed in *rpd1/rmr6* compared to the only 71 up-regulated in B73 point to a higher conservation of *pol IV* de-repressed targets in contrast to the silenced ones, which could instead represent indirect, not-shared targets (Additional file 10).

**Fig. 5:**
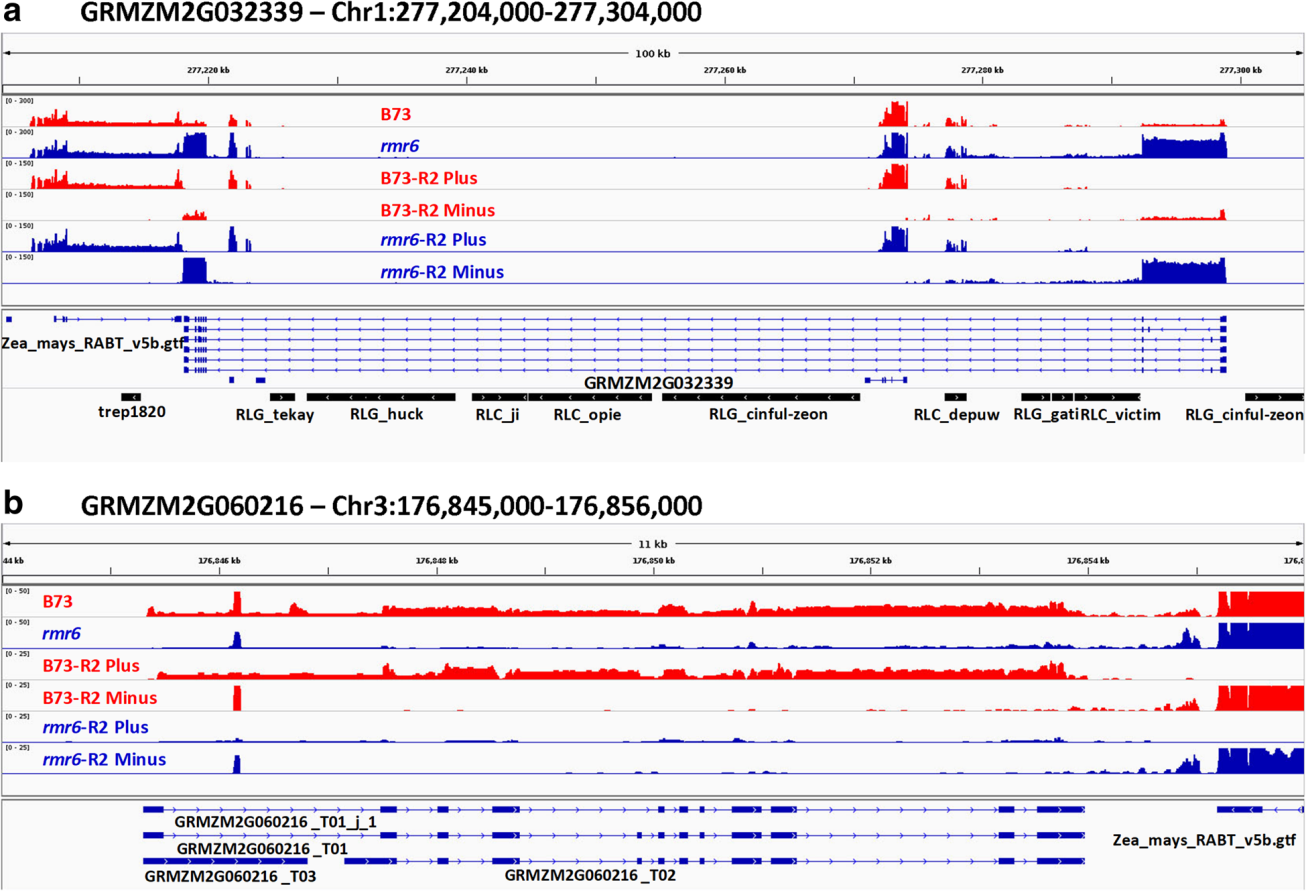
Genome browser view of RNA-Seq reads mapped at *rpd1/rmr6* misregulated transcription factors. Genome browser (IGV - Integrative Genomics Viewer; http://software.broadinstitute.org/software/igv/) views of B73 (red) and *rpd1/rmr6–1* mutant (blue) RNA-Seq reads (normalized to the total of mapped reads) over the ZmMADS4 (**a**) and LG2 (**b**) transcription factors. Total mapped reads (replicates 1 and 2) and strand-specific mapped reads (replicate 2) are reported. Repeats depicted in (**a**) are only a small part of the more than thirty TEs annotated in the long central intron

Among the shared differentially expressed genes no GO terms were enriched; however the DNA integration (GO:0015074) term was the most represented between the *rpd1/rmr6* up-regulated genes, suggesting an over-representation of TE-related genes in this set. About one third of them are represented by de novo annotated intergenic loci (Additional file 10; see also [40]); many well-characterized protein-coding genes also were differentially expressed in *rpd1/rmr6* leaves, as previously shown in seedlings [41]. TE-controlled over-expression of the homeodomain leucine zipper IV (HD-ZIP IV) *ocl2* has also been confirmed in leaves, together with the identification of two MADS-box transcription factors strongly up-regulated by Pol IV loss of function: ZmMADS4 (GRMZM2G032339; Fig. 5a) and ZmMADS52 (GRMZM2G446426). The former was identified as a fundamental regulator of both floral induction and inflorescence development [53], while the latter was not fully characterized although its high expression in shoot tips and inflorescence primordia (http://www.qteller.com/qteller4/) suggests its involvement in both vegetative to reproductive phase transition and floral development.

Among down-regulated genes we found another regulator of leaf development: the basic region/leucine zipper motif (bZIP) transcription factor *liguleless2* (*LG2*/GRMZM2G060216; Fig. 5b) that functions in narrowing the region from which the ligule and auricle develop in maize leaf, and in regulating vegetative to reproductive phase transition [54, 55].

Mutant analysis revealed that *ZmMADS4* over-expression resulted in an early flowering phenotype [53], while in the absence of *lg2* gene function the normal vegetative to inflorescence phase transition might be delayed [55]: the net result is a significant delay in flowering in *rpd1/rmr6* mutant plants [28].

### Prediction of RdDM direct targets by *rmr6–1* and *mop1–1* misregulated genes integration

Pol IV transcripts are used as templates by the Pol IV-associated RNA-dependent RNA polymerase 2 (RDR2 [14, 16]), and processed into double-stranded RNA (dsRNA) to enter the RdDM silencing pathway. To identify those genes that are likely direct targets of RdDM we intersected *rpd1/rmr6–1* misregulated genes with those identified in two independent studies on the *mop1–1/rdr2* maize mutant [42, 43].

Considering the two different tissues analyzed in *mop1–1* mutant - shoot apical meristems [43], and immature ears [42], we found 27 genes that were up-regulated and 7 that were down-regulated in both genotypes (Additional files 11 and 12): they could represent the genes most likely directly regulated by Pol IV/RDR2 epigenetic regulation pathway.

The first shared RdDM up-regulated gene was an MFP1 attachment factor 1 (GRMZM2G479245), that contains a nuclear envelope targeting domain (WPP) necessary for correct protein targeting and cell division stimulation. The second candidate is GRMZM2G012160, a gene encoding for cystatin2, a cysteine proteinase inhibitor. Its overexpression in Arabidopsis increases tolerance to abiotic stresses (i.e. salt, osmotic, cold stress; [56, 57]. This observation further confirms the previously hypothesized stress-like effect on genome regulation caused by epiregulator mutations [40]. The up-regulation of the previously described *ZmMADS4* (GRMZM2G032339) gene in both mutants underlines the role of RdDM in controlling important aspects of plant development such as flowering and reproduction.

One of the shared down-regulated genes is GRMZM2G131756, the putative ortholog of REPRESSOR OF SILENCING 1 (ROS1)/ DEMETER-LIKE1 (DML1), an Arabidopsis DNA glycosylase protein that actively demethylates DNA [58, 59]. ROS1 is down regulated in several Arabidopsis RdDM mutants [60] and, as previously hypothesized, a reduction in demethylation activity could cause the downregulation of several secondary targets in RdDM mutants [42].

### Pol IV silenced loci are enriched in class I TEs

**Fig. 6:**
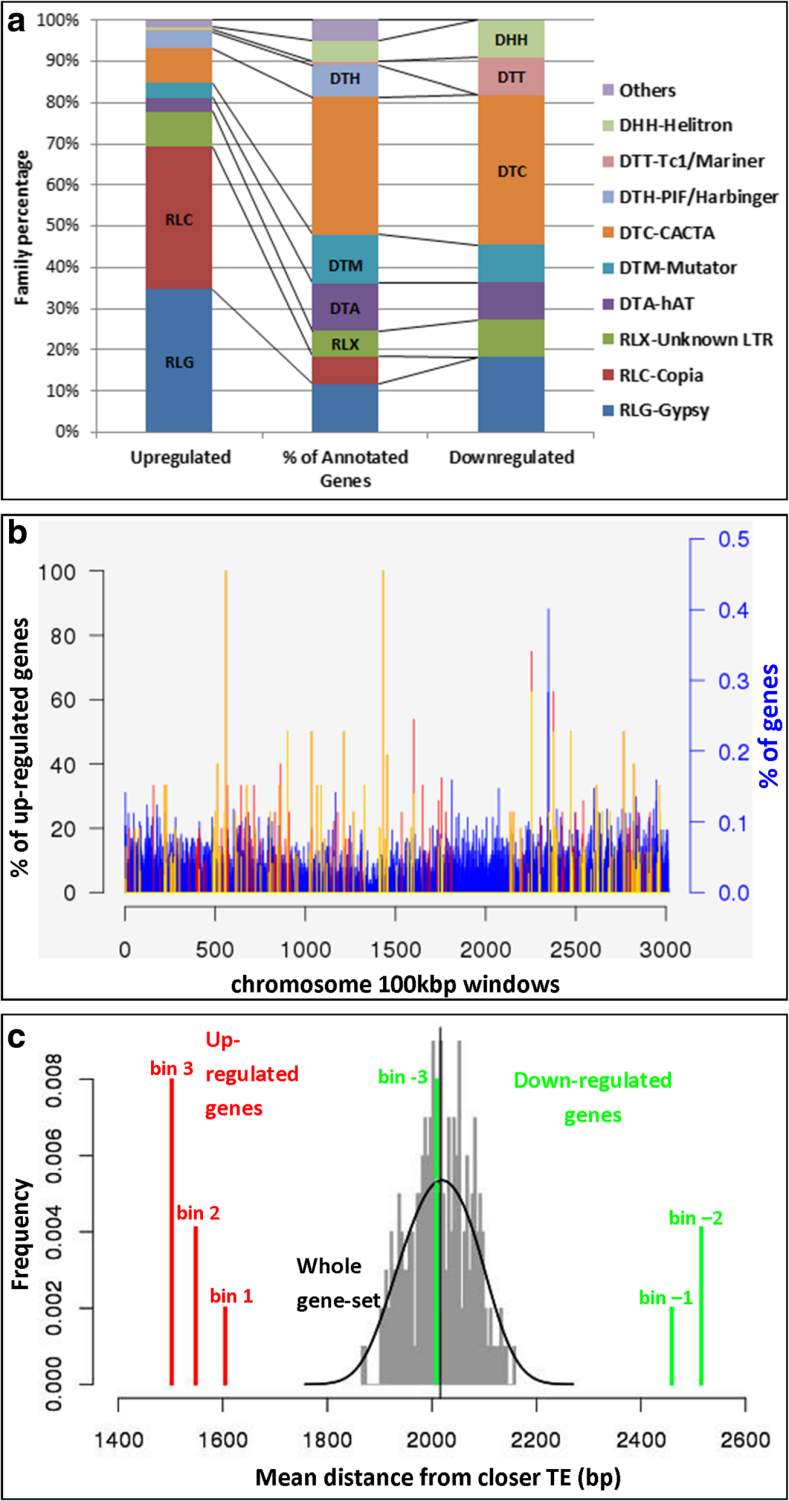
Pol IV silenced loci are enriched in class I transposable elements and are preferentially co-localized. Relative percentage of up- and down-regulated TE superfamilies, compared to their abundance in the gene annotation (**a**), indicate that the RLC-Copia and RLG-Gypsy class I retroelement super-families are notably enriched among the *rpd1/rmr6* up-regulated genes, while DTC-CACTA and other class II DNA transposon super-families ore over-represented among down-regulated genes. The distribution plot (**b**) of the differentially expressed genes along chromosome 1 indicates the preferential co-localization of *rpd1/rmr6* de-repressed genes. The chromosome was divided in 100 Kbp not-overlapping windows and for each window the percentage of genes (with respect to the total chromosome genes; blue bars) and over-expressed genes (with respect to the window gene content) are reported. Yellow and red bars depict the window percentage of up-regulated genes shared in at least three or two independent comparisons, respectively. Permutation test using the regioneR R package (**c**) showed that *rpd1/rmr6* strongly up-regulated genes (bin 3) were closer to TEs than moderately or slightly up-regulated ones (bins 2 and 1): 1500 bp, 1547 bp and 1605 bp are the corresponding average distances from the closest TEs for each bin (the average distance for the whole gene set is 2017 bp; grey distribution). Similarly, slightly and moderately down-regulated genes (bins −1 and −2) were located progressively farther from TEs (2461 bp and 2514 bp, respectively), while genes of bin −3 (those showing the strongest down-regulation) did not shown any positional deviation from the whole gene set (2008 bp average distance). Differentially expressed genes for permutation analysis (log2FC > |1|, FDR < 0.05) were obtained with Cuffdiff starting from all the sequenced samples .This graph was produced by combining the single graphs obtained from the independent analysis of up- and down-regulated bins

Despite the important role that emerged of TEs in controlling gene expression in the absence of a functional Pol IV, the overall TE transcription appeared modest. The loci with at least one transcript previously classified as HC-TE or pr-TE represented about 20% (176 genes) and 15.5% (11 genes) of the up- and down-regulated genes, respectively, while these TEs represented 16% of the whole gene annotation. Breaking the differentially expressed TE-related gene set into distinct families (Fig. 6a, Additional file 13), the RLC Copia and the RLG Gypsy class I retroelement super-families are particular enriched among the *rpd1/rmr6* up-regulated genes, while DTC-CACTA and other class II DNA transposons are included among the eleven down-regulated, TE-related genes. To determine if distinct TE families were differentially expressed in the mutant leaf transcriptome compared to wild type, we re-mapped all the sequenced reads against the genomic sequences representative of 1526 full-length TE families retrieved from the Maize transposable element database (http://maizetedb.org/~maize/). In this analysis, exclusively uniquely mapped reads were selected to perform a differential expression analysis with edgeR [61], which led to the identification of 43 strongly up-regulated and 13 down-regulated transposon families in *rpd1/rmr6* mutant (Additional file 14). In addition, this analysis confirmed the de-repression of many LTR retrotransposons (RLG and RLC superfamilies), together with many type II Mutator elements, while RLX and CACTA elements were the most abundant among the silenced TEs.

The RLG Ahoru was the element with the highest over-expression in *rpd1/rmr6* mutant and it is also one of the most represented among differentially expressed newly annotated intergenic loci. If its RMR6 silencing is undeniable, manual inspection of read alignment files revealed that all the uniquely mapped reads were restricted to a 300 bp long region situated immediately upstream of the GAG coding region, while all the other DE elements presented reads equally distributed on the whole TE sequence. This 300 bp long region is highly repeated in the B73 genome (at least 100 near-perfect matches by blastn analysis) and could correspond to a fraction of the 385 Ahoru partial fragments annotated in the maize genome by [31]. These authors reported for Ahoru only one single full-length insertion and 385 partial fragments of 384 nt average length, which are probably subjected to RdDM silencing.

*rpd1/rmr6* over-expressed LTR families are low-copy-number families (Additional file 14) that were previously shown to be most often inserted in or near genes [31], confirming the recently proposed role of RdDM in controlling near TE inserted genes.

### Pol IV silenced loci are co-localized and closer to TEs

Distribution analysis of the 880 *rmr6* up-regulated genes along the maize genome revealed that about one third of them are in clustered positions. We identified more than one hundred 100 Kbp genome windows, containing 2 to 10 *rpd1/rmr6* de-repressed genes (Fig. 6b and Additional file 15). The 71 down-regulated genes resulted instead uniformly distributed along the maize chromosomes, being included in 67 independent genome windows (Additional file 16). As control, the distribution of the up-regulated genes along maize chromosomes was compared with the average distribution of 100 independent datasets, each composed of 880 genes randomly selected from the list of 40,457 expressed genes. The two distributions resulted strongly statistically different (*P* = 1 × 10^−16^, by Wilcoxon test): the random genes resulted more uniformly distributed along the genome (Additional file 15) and on average they were included in 876 genome 100 Kb windows versus the 737 including the up-regulated genes (143 up-regulated genes −16.25%- resulted then co-localized in the same window of other up-regulated genes).

Manual inspection of clustered up-regulated genes revealed that TEs were inserted within each cluster or nearby, with a clear predominance of RLC and RLG superfamilies of LTR retrotransposons (Additional file 17). In addition, we also found in many co-expressed clusters up-regulated genes that were significantly up-regulated in only one or two of the previously described independent comparisons (Fig. 6b, Additional file 15).

Further investigations on the association between differentially expressed genes and nearby TE insertions revealed that up-regulated genes are closer to TEs than the average of genes in the genome (*P* < 0.005): their average distance from TEs was 1550 bp compared to 2017 bp for the whole gene set in the genome, obtained by permutation test (Additional file 18). On the contrary average distance from TEs of down-regulated genes was longer (2374 bp; P < 0.005) than that of genes in the genome (Additional file 18).

When the differentially expressed genes were subdivided based on their fold change variation (Additional file 19), a direct correlation between transcriptional variation and distance from closest TEs was even more evident, especially for up-regulated genes. *rpd1/rmr6* strongly up-regulated genes (bin 3) are closer to TEs than moderately (bin 2) or slightly up-regulated ones (bin 1), being 1500 bp, 1547 bp and 1605 bp from closest TE, respectively (Fig. 6c). Similarly, slightly (bin −1) and moderately (bin −2) down-regulated genes are located progressively farther from TEs (2461 bp and 2514 bp, respectively). On the contrary, permutation tests revealed that the genes showing the strongest silencing in *rpd1/rmr6* mutant (bin −3) are as distant from TEs as the average of the maize genes (2008 bp; Fig. 6c).

### *pol IV* misregulated loci are located on regions with distinct DNA methylation profiles in B73

**Fig. 7:**
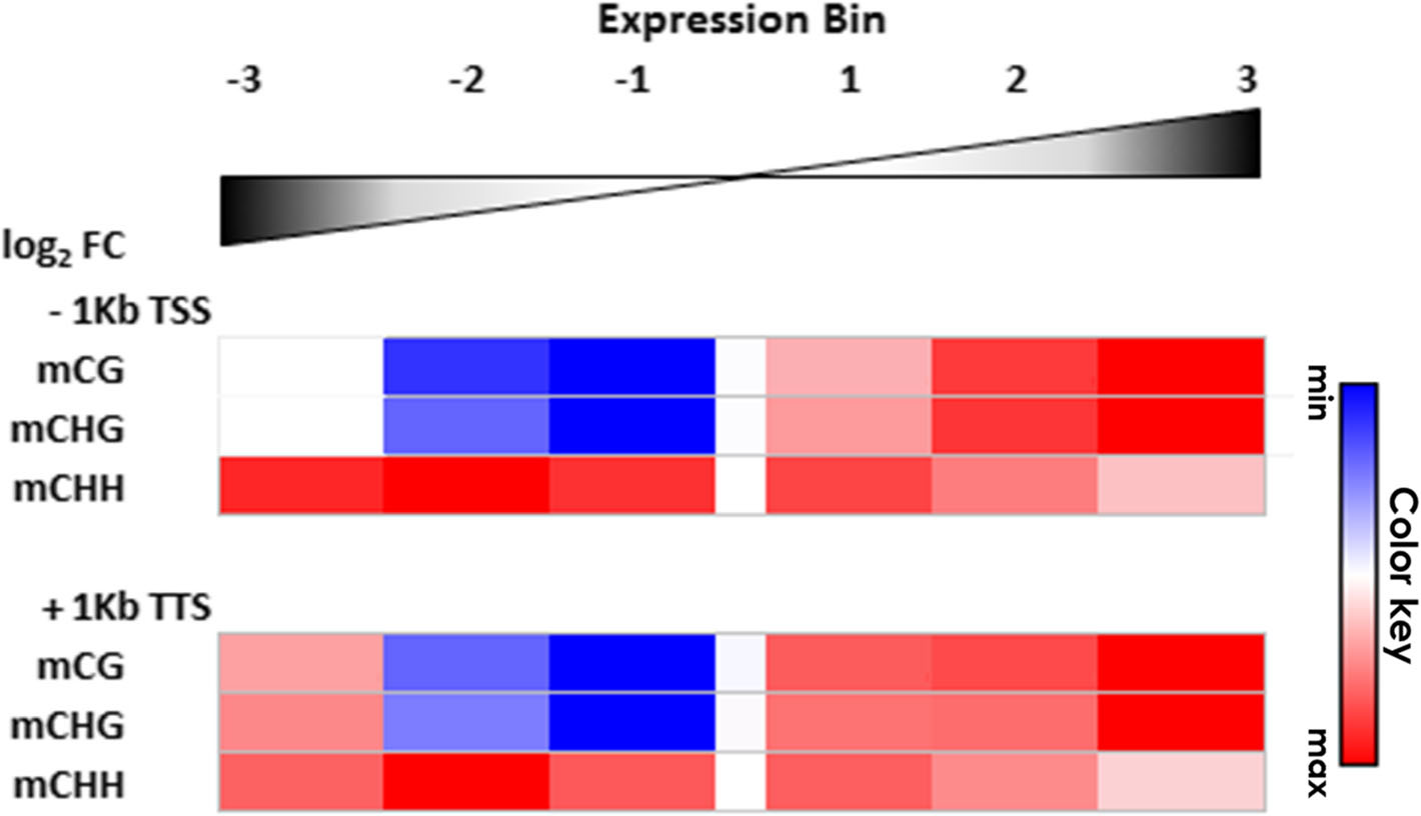
DNA methylation profiles at differentially expressed gene flanking regions. Methylation levels in each context (CG, CHG and CHH) were computed for the flanking regions (2 Kb for CG and CHG, 1 Kb for CHH; see Methods) of differentially expressed genes and compared to the average of genes in the genome using regioneR permutation approach. For each bin, in each flanking region, the average methylation level is reported by means of heatmaps. Gradients of red and blue indicate methylation values statistically above or below the average of the whole gene set. Average methylation values and associated *p*-values are reported in Additional file 21

The results described above further demonstrated the interplay between TEs and gene misregulation. They also indicated the existence of multiple, interconnected mechanisms for gene regulation: if proximity to TEs directly correlates with the magnitude of gene over-expression in *rpd1/rmr6* mutant, the strongly silenced genes do not show any particular positional deviation from them. Because Pol IV, throughout the RdDM pathway, is required for the establishment/maintenance of DNA methylation, in particular at the level of mCHH islands, we determined the locus-specific DNA methylation status at identified misregulated loci. B73 genome-wide map of cytosine methylation was retrieved from a published whole-genome bisulfite sequencing study on seedlings [62] to create a snapshot of DNA methylation levels. DNA methylation profiles in each context (CG, CHG and CHH) were evaluated on the flanking regions of the misregulated genes, subdivided in expression bins as previously described (see Methods; Additional file 19). Methylation plots and permutation analysis indicated that *rpd1/rmr6* up-regulated genes are flanked by CG and CHG highly methylated regions compared to the whole gene set (and the enrichment seems to be directly correlated to the degree of overexpression), while down-regulated ones, belonging to bins −1 and −2, had lower CG and CHG methylation on both flanking regions (Fig. 7, Additional files 20 and 21). Special reference needs to be made to those genes showing the strongest down-regulation in *rpd1/rmr6* (bin −3): in their flanking regions these genes had CG and CHG methylation levels comparable to average levels observed for the whole gene set and higher than those found in bins −2 and −1, (Fig. 7, Additional files 20 and 21).

On the contrary, the genes showing the strongest up-regulation in *rpd1/rmr6* (bin 3) are flanked by slightly CHH methylated regions, compared to other up-regulated (bins 1 and 2) and down-regulated ones (Fig. 7, Additional file 21), where mCHH islands are clearly detectable as sharp methylation peaks (Additional file 20).

### Relationship between Pol IV activity and duplicated gene regulation

Syntenic comparison of the maize genome with the genomes of other grass species revealed that maize experienced a whole genome duplication 5 to 12 million years ago [63, 64], which resulted in two distinct subgenomes each of which originally contained a complete set of genes and regulatory sequences [65]. In many cases, one of the two copies of a gene was lost from the genome in a process known as fractionation; however 3000 to 5000 gene pairs are retained in the modern maize genome [65]. The maize gene complement can therefore be divided into three categories: pairs of duplicate genes shared by both subgenomes (both genes have been retained), single-copy genes present in only one subgenome, also called singletons (the corresponding homeolog has been lost since tetraploidization), and genes that cannot be assigned to a subgenome. Subgenome partitioning revealed that the maize recessive subgenome 2 is characterized by higher gene loss than the dominant subgenome 1. In addition, among retained pairs, the gene in the recessive subgenome tends to have lower expression than its dominant homeolog [65]. On the contrary the third category is characterized by a lack of syntenic orthologs in the genomes of other grass species, indicating that most of these nonsyntenic genes are created by single gene duplication mechanisms, likely after the maize whole genome duplication [66].

To explore the role of Pol IV in duplicated gene regulation and genome dominance, we analyzed the paralog expression dynamics in B73 and *rpd1/rmr6* mutant leaves. Starting from the set of validated gene models (FGS: filtered gene set; 39,656 genes), we identified 23,895 genes passing the previously described expression filter. Among these, we developed two sets of gene lists: i) genes significantly up-regulated in *rmr6* mutant (log2FC > 1, FDR < 0.05) or ii) genes significantly down-regulated (B73 overexpressed, log2FC < −1, FDR < 0.05). Expression fold changes for this analysis were obtained with Cuffdiff on the previously described “All-test-set”. Frequencies of each gene set partitioned into the two subgenomes (http://www.skraelingmountain.com/datasets/maize_indexed_by_subgenome.csv) and the five different classes are reported in Additional file 22. Note that the total numbers of up- and down-regulated genes are quite similar in between the FGS, that were selected based upon sequence similarity to other species and the existence of a putative full-length coding sequence to exclude transposons, pseudogenes and other low-confidence structures.

**Fig. 8:**
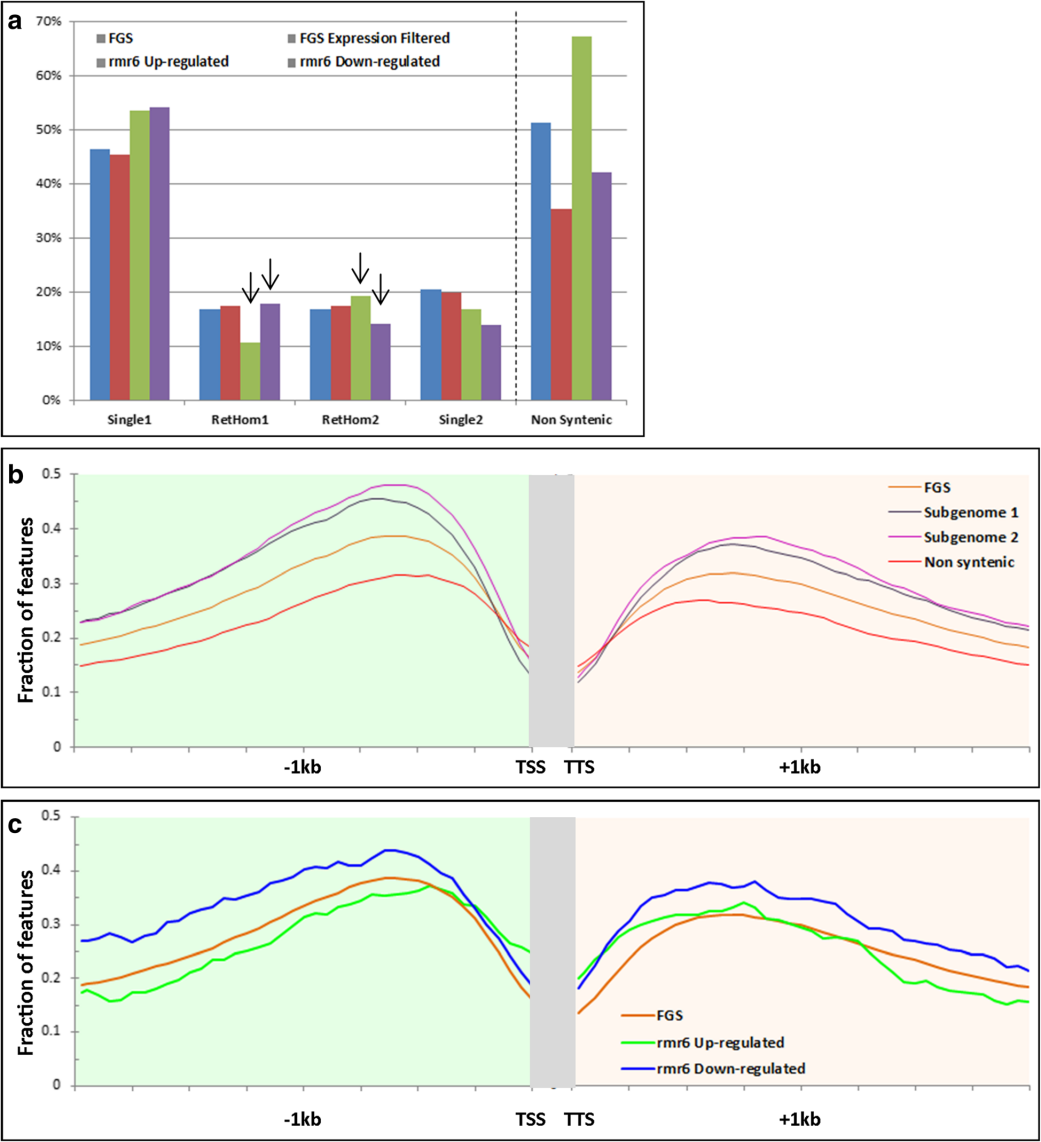
Correlation between genome dominance, Pol IV-mediated gene silencing and siRNA loci occupancy. Subdivision of differentially expressed gene models (log2FC > |1|, FDR < 0.05) into singletons, duplicates in the two subgenomes, or non-syntenic genes show an enrichment for subgenome 2 homeologs and non-syntenic genes within the *rpd1/rmr6* up-regulated targets (**a**). Percentages of singletons and duplicates were calculated on the total of genes assigned to subgenomes, while percentages of non-syntenic genes were referred to the total of FGS genes and are reported on an independent scale. The distribution plots of siRNA loci in flanking regions of genes belonging to different subgenome (**b**) reveal that subgenome 2 genes are more likely to have flanking 24 nt-siRNAs than subgenome 1, while non-syntenic genes are less covered by 24 nt-siRNA in their upstream region than syntenic ones. Similarly, the distribution of the 24sRNA loci along the flanking regions of *rpd1/rmr6* up- or down-regulated genes (**c**) shows that mutant up-regulated genes tend to have fewer siRNA in their flanks than-downregulated ones

Compared to the “reference” FGS expression filtered gene set, *rmr6* up- and down-regulated genes present two diametrically opposed enrichments in retained homeolog genes: for the *rpd1/rmr6* up-regulated genes the percentage of genes in subgenome 2 is significantly higher than it is for the expressed gene list, while for down-regulated genes the percentage is higher for subgenome 1 than subgenome 2 (Fig. 8a; Additional file 22). If the principal component of subgenome 2 over-representation in *rpd1/rmr6* mutant over-expressed genes was linked to the peculiar behavior of duplicate genes retained in both subgenomes, deeper analysis revealed variations in the percentage of both subgenome’s singletons in Pol IV up- and down-regulated genes (Fig. 8a). Further massive deviations were observed at level of genes not assigned to any subgenome that, even if less represented among expression filtered genes, were highly over-represented in *rpd1/rmr6* up-regulated (67%) than in B73 (42%; Fig. 8a and Additional file 22) induced genes.

A breakdown on paralog pairs differential expression between the two genotypes was obtained identifying among the DE genes, the retained homeolog pairs in which at least one paralog is differentially expressed. For 62.5% of *rmr6* up-regulated pairs, differential expression was caused by the recessive paralog, while this value dropped to 38.7% for the down-regulated pairs (Table 2).

**Table 2:** Genes with duplicates in subgenome 1 and 2 differentially expressed in one genotype

DE Class	DE Paralog pairs^a^	Homeolog1 DE	Homeolog2 DE	Both homeologs DE
*rpd1/rmr6* Up	64	21	40	3
*rpd1/rmr6* Down	111	61	43	7

Contribution of genes with duplicates in both subgenomes on differential expression of paralogs (^a^DE paralog pairs identified based on at least one paralog being differentially expressed)

### 24 nt-siRNAs preferentially target the recessive subgenome 2 while they are depleted from non-syntenic genes

Recent studies on the polyploid *Brassica rapa* revealed that 24 nt siRNAs target and cover the upstream region of retained genes preferentially when located in the recessive subgenome [67], hypothesizing a role for RdDM in genome dominance regulation.

To confirm this finding also in maize and to dissect the role of Pol IV-mediated siRNAs in genome dominance we analyzed the distribution of the identified 24sRNA loci along the flanking regions of FGS gene set (our reference) and different subgenome classes.

As reported in Brassica, an indirect correlation between genome dominance and siRNA coverage was observed in maize: genes of the recessive subgenome 2 are preferentially siRNA-enriched in the upstream region, without differences between retained homeologs and single copy genes (Fig. 8b, Additional file 23), while these differences between the analyzed gene sets are less evident in the downstream regions. In addition our analysis points to the very low frequency of siRNA loci in the upstream region of non-syntenic genes.

It is worth noting that non-syntenic genes are enriched in TE insertions in their upstream region compared to syntenic ones (Table 3), corroborating the hypothesis that they originated by transposon shuffling of existing genes [68, 69], but also again confirming the weak relation between TEs silencing and siRNA loci presence and expression.

**Table 3:** Frequency of TE insertions in the upstream regions of syntenic and non-syntenic genes

	Genes	TE_1Kb Upstream TSS		TE_-500/+500 bp TSS	
SUB1	12,190	1935	15.87%	828	6.79%
SUB2	7175	1233	17.18%	557	7.76%
Non-syntenic	20,291	5170	25.48%	3095	15.25%
	39,656	8338	21.03%	4480	11.30%

Finally, the distribution analysis of the 24sRNA loci along the flanking regions of FGS gene set and *rpd1/rmr6* up- or down-regulated genes further confirmed the lack of a direct association between siRNA loci abundance and *rpd1/rmr6* de-repression, with mutant up-regulated genes that tend to have fewer siRNA in their flanks than-downregulated ones (Fig. 8c).

## Discussion

### Loss of Pol IV activity affects gene regulation and plant development

Differential expression analysis in *rpd1/rmr6* mutants identified from 1 to 4 thousand differentially expressed genes, depending on the bioinformatics tools applied. The vast majority of these genes are up-regulated in *pol IV* mutant, confirming the repressive role of RdDM on gene transcription. Differentially expressed genes include direct Pol IV repressed genes and indirect targets, resulting from genome-wide changes triggered indirectly by the loss of Pol IV activity. A subset of differentially expressed genes commonly de-regulated in *rpd1/rmr6* and in the *mop1/rdr2* mutants, even in the different tissues analyzed, have been identified as likely direct targets of RdDM.

According to previous studies [41–43], the loss of a functional RdDM pathway results in both misregulation and changes in gene expression of many genes important for plant development, including difference in miRNAs expression [24] which might cause the observed pleiotropic developmental defects and mutated phenotypes in both *rpd1/rmr6* and *mop1* plants. Although we did not detect any differentially expressed miRNA in *rpd1/rmr6* developed leaf [23] the alteration of multiple regulatory pathways in meristematic tissue may be considered an important cause of changes in genes expression also for the leaf tissues. As a consequence, the loss of leaf polarity observed in *rpd1/rmr6* mutant [28], and the resulting different distribution of cell types in the leaf tissues, could explain the indirect misregulation of specific developmental-regulated genes.

The down-regulation of DNA demethylase ROS1/DML1 could instead be caused by the reduction of mCHH levels in *rmr6* and *mop1* mutants: indeed, Arabidopsis ROS1 expression is promoted by DNA methylation in its promoter region. This methylation-induced expression results in fine tuning of demethylase activity in response to methylation alteration, which allows the fine methylation homeostasis between gene expression and genome defense [60].

### Loss of Pol IV activity globally affects genome regulation

In addition to the relatively low number of differentially expressed genes, a deep analysis of our total RNA-Seq data demonstrated that loss of Pol IV activity caused a global increase in the transcribed fraction of the maize genome. In differential expression analysis, low-count genes are indeed unlikely to be found as differentially expressed compared to highly expressed, because of the low statistical power in calling significant differential expression. The rise in the fraction of transcribed genome, which is not restricted to its repetitive portion, is instead caused by small increases at many loci and is accompanied by different gene expression distribution profiles between wild type and mutant plants. *rpd1/rmr6* mutation resulted in a global increase in the basal expression of a vast number of genes compared to B73, with greater differences at the level of newly annotated loci and previously identified non coding sRNA precursors [40].

These loci are supposed to be normally transcribed by Pol IV/Pol V RNA polymerases to enter the canonical RdDM pathway, so their expression increase in the *rpd1/rmr6* mutant seems to be contradictory. The reduced length (25-45 bp), low expression and short life of Pol IV-transcripts [16, 17, 33] prevent their identification in classical RNA-Seq experiments. These characteristics exclude a biased enrichment of Pol II transcripts in *pol IV* mutant: Pol IV transcripts are indeed unsuccessfully detected and analyzed in both wild-type and *rpd1/rmr6* mutant. Therefore, lack of *pol IV* RNAs in *rpd1/rpd1* mutant could not lead to artefactual overexpression of Pol II transcripts compared to wild-type B73, i.e. due to read normalization bias. The observed increased expression in smallRNA precursors should then be ascribed to the increased activity of either RNA Pol II or Pol V. In the absence of a functional RdDM pathway, an involvement of these repeats-related transcripts in the non-canonical Pol II–RDR6-dependent RdDM pathway has been demonstrated [70, 71]. This silencing pathway has been associated to TAS loci and newly integrated transposons and relies on Pol II transcripts that are copied by RNA-dependent RNA polymerase 6 (RDR6) and processed by Dicer-like family proteins (DCL2 and DCL4) into 21–22-nt siRNAs. When transposons escape silencing, and are transcribed by Pol II, their mRNAs can be recognized and degraded, generating 21–22 nt small RNAs that can also guide initial DNA methylation, interacting with AGO4 or AGO6 to elicit RdDM in a Pol V–dependent manner [1, 72]. Interestingly, 22 nt smallRNAs showed slightly higher accumulation level in *rpd1/rmr6* and of the few smallRNA loci found up-regulated in *rpd1/ rmr6* relative to wild-type, most of them were dominated by 22 nt RNAs [23].

Altogether, these findings suggest an involvement of Pol II-RDR6 non-canonical RdDM silencing pathway in overcoming the lack of a functional Pol IV in maize.

### The shady role of TEs in RdDM

Even if the overall TE transcription appears modest and a low percentage of differentially expressed genes could be traced back to TEs (20% for up-regulated and 15.5% for down-regulated genes; compared to the 16% of TE-related genes annotated), the majority of non-coding siRNA precursors transcripts are related to TEs. The comparisons of expression profiles also showed that TEs could directly affect expression of the closer genes, clearly indicating transposons as important targets of Pol IV-mediated silencing.

Several studies on Arabidopsis demonstrated that TEs could affect the expression of proximal genes via different mechanisms [73–75]. While RdDM is reported to target transposons and repeats mainly at the level of smaller and younger transposons [76–79], these elements are not generally mobilized in mutants defective in RdDM in Arabidopsis. Our results confirm the preferential targeting of RdDM to younger TEs in maize, also reinforcing the traditional regulatory role of transposons.

We indeed showed that TEs located nearby genes (in particular in the interval − 500/+500 bp with respect to the TSS, but also downstream of the TTS) are the main effectors of Pol IV-mediated gene silencing release in *rpd1/rmr6* mutant. These results confirm TEs starring role as “controlling elements” of gene regulation in maize. Moreover, we confirmed both the previously observed lower expression of genes close to TEs compared to TE-lacking genes in Arabidopsis [74, 80], and that this positional silencing is mediated by a functional RdDM pathway. With the recently demonstrated contribution of TEs to the regulation of maize genes in response to abiotic stresses [39], we can speculate the existence of highly complex epigenetic and regulatory interactions between TEs and the transcriptional regulation of their host genome during whole maize development in response to environmental challenges and adaptation.

### Pol IV silenced loci are independent of siRNA loci

Pol IV is essential for the biogenesis of siRNAs and 24 nt siRNAs are dramatically reduced in *pol IV* and other RdDM mutants. Our results instead indicated that genes with associated siRNA loci are less affected by gene silencing release observed in *pol IV* loss of function mutant. These apparently contradictory results reinforce the discussion on the actual mechanism of Pol IV-mediated gene silencing in plants and in maize in particular. In Arabidopsis, a progression of transposon DNA methylation from the Pol IV/Pol V/RDR2 self-reinforced RdDM to an RdDM-independent silenced state that involves MET1- and CMT3/CMT2-mediated CG and CHG methylation, together with the deposition of the repressive histone mark H3K9me2, have been shown [7]. Similarly, Pikaard and co-workers demonstrated the existence of a double lock mechanism at RdDM loci [47]. Silencing of these loci, stabilized and memorized through histone modifications and CG DNA methylation, is not affected by Pol IV loss of function. It could thus be hypothesized that silencing of the vast majority of transposons is still maintained in both maize (in which CG and CHG methylation is commonly found and is only partially lost in RdDM mutants [32, 34]) and Arabidopsis in the absence of a functional RdDM pathway. In addition, siRNAs produced by Pol IV/RDR2 pathways at these stable silenced loci could act in trans to silence homologous TEs, repeats and genes with young TEs inserted nearby. The approach used for siRNA loci analysis was indeed aimed to identify the site of transcriptional origin of smallRNA, not all their possible targets [23, 81]. An action in trans of siRNAs, previously associated to transgenes silencing [82] and allelic regulation in hybrids [83], might therefore be involved in maintaining the repression of genes and TEs lacking siRNA direct transcription, the silencing of which will fail in the absence of a functional Pol IV.

### Loss of Pol IV activity results in spreading of chromatin features from genes to TEs or vice versa

Differently from methylation in CG and CHG contexts, CHH methylation surrounds genes and has been found associated only with certain types of transposons [25, 34, 84]. Independent analyses (identification of TE-related differentially expressed genes and reads re-mapping against the curated sequences of 1526 full-length TE families) revealed that loss of RdDM activity results in the preferential transcriptional activation of class I LTR retrotransposons belonging to RLG-Gypsy and RLC-Copia superfamilies. Furthermore, many *rpd1/rmr6* over-expressed LTRs consist of low-copy-number families that were previously shown to be most frequently inserted into or nearby genes [31], confirming the results previously discussed and the recently proposed role of RdDM in repressing transposons in accessible, active chromatin environments near genes [25, 33].

Members of RLC and RLG superfamilies are preferentially inserted in the flanking region of the genes showing strong up-regulation in *rpd1/rmr6* (they are associated to 66% of bin 3 genes with a flanking TE) and were previously shown to be characterized by high levels of CG and CHG methylation and lower levels of mCHH compared to class II DNA TEs [25, 84]. Re-analysis of existing methylation data confirmed the high levels of mCG and mCHG and relatively low levels of mCHH at the up-regulated genes: loosening of mCHH at these regions, predictable in *rpd1/rmr6* mutant, may be accompanied by additional loss of CG and CHG methylation within the transposon and increased expression of both TEs and genes as described in *mop1–1* mutant [33]. Accordingly, the loss of functional Pol IV activity and the linked epigenetic regulation resulted in the spreading of accessible chromatin states with the transcriptional de-repression of widespread chromosome regions (up to 100 Kbp) in *rpd1/rmr6*, with several loci commonly misregulated.

On the contrary, we found that CACTA class II transposons are mostly included in the *pol IV* down-regulated TE exemplars, as previously observed in *mop1–1* mutant [43]. Class II DNA TEs have been described more frequently associated to 24 nt sRNAs and mCHH islands [23, 33], and are also preferentially found in flanking regions of down-regulated genes (primarily CACTA, Mutator and hAT superfamilies). Given the above discussed role of mCHH islands as borders between open chromatin and heterochromatin, it could not be excluded that the lower expression levels of these genes in *rpd1/rmr6* compared to wild type are caused by the spreading silencing marks from heterochromatic flanking regions to the neighboring genes. Even if this phenomenon was not confirmed by a previous study on the *mop1–1* mutant [33], it is further corroborated by the relatively high frequency of spreading LTR elements (LTR retrotransposons that exhibit spreading of silencing marks such as DNA methylation and H3K9me2 to the neighboring sequences [85]) found closer to the strongly silenced genes (bins −2 and −3).

Altogether, this evidence indicates that TEs located nearby genes (or, alternatively, genes with nearby TE insertions) are the preferential targets of RdDM in maize, in agreement with the suggested role of RdDM on the borders between open euchromatic gene-rich regions and inaccessible heterochromatic regions with elevated CG/CHG methylation [25, 33].

### Does RdDM guide the evolution and stability of maize genome?

The results also indicated the existence of additional epigenetic TEs silencing mechanisms: in the absence of Pol IV-derived 24 nt-siRNA, Pol II–RDR6-dependent 21–22 nt siRNAs could efficiently maintain TE silencing, at least in the first generation (*rpd1/rmr6* mutants present progressive trans-generational degradation in plant quality compared to non-mutant siblings; [27, 28]). Furthermore, in other organisms transposons are silenced in the absence of additional RNA polymerases and specialized transcriptional machineries. Recent works have indicated that the Pol V branch of RdDM pathway has reached the maximum complexity in flowering plants: this might suggest that RdDM serves an as-yet-undefined function that has been especially important for this specific plant group. Consequently, speculating on the role of RdDM in flowering plants, a new role in accelerating the diploidization of polyploid genome was proposed for this pathway [44].

In the *pol IV* mutant the differentially expressed genes revealed an unexpected representation in the two maize subgenomes. The maize genome consists of two subgenomes derived from an ancient allotetraploidization event: the two subgenomes are distinguishable because the dominant subgenome 1 conserved more genes than subgenome 2, a phenomenon called “genome dominance”. Additionally, among retained gene pairs, the gene on the dominant subgenome tends to be more expressed than its recessive homeolog [65, 66]. A greater proportion of the genes up-regulated in the *rpd1/rmr6* mutant belong to subgenome 2 and represent the recessive homeolog of duplicated pairs. Similar expression enrichment for recessive subgenome 2 members was observed exclusively in the transcriptome of mature pollen [86] and some of the genes specifically de-repressed in *rpd1/rmr6* mutant have previously been indicated as pollen specific. Maize RNA-Seq data of 25 tissues derived from six independent studies (http://www.qteller.com/qteller4/) revealed that the expression of the *RMR6* gene is undetectable in pollen cells (http://qteller.com/bar_chart.php?name=Zm00001d031459&info=). Similarly, RdDM components were found down-regulated during pollen development in Arabidopsis [87–89], with a consequent depletion of CHH methylation pollen transposons. This phenomenon might either represent an ancient mechanism for transposon recognition with the following CHH methylation restoration in the Arabidopsis embryo after fertilization occurs [88], or be necessary to allow the expression of pollen specific genes commonly silenced in vegetative tissues by RdDM.

The high percentage of *rpd1/rmr6* up-regulated genes not assigned to subgenomes 1 or 2 (67% of up-regulated genes) is quite unusual, since they represent 50% of FGS genes and are globally less expressed than syntenic ones. Non-syntenic genes are considered “younger” than syntenic ones because they evolved after the last whole genome duplication (e.g., by modular rearrangement of protein encoding domains [90], by the transposition of existing genes [68], or by exon shuffling from helitrons and other transposons leading to fusion genes [69]). Recently, non-syntenic genes have been associated with heterosis and hybrid vigor manifestation [91].

It is worth noting that non-syntenic genes are enriched in TE insertions in their upstream region compared to syntenic ones, corroborating the previously reported hypothesis that they originated by “recent” transposon shuffling of existing genes. Moreover, non-syntenic genes are less covered by 24 nt siRNA in their upstream region than syntenic genes, again confirming the weak association between TEs near genes and siRNA loci occupancy. This lower siRNA abundance is in accordance with their globally lower expression: a positive correlation between gene expression level and the occupancy of upstream siRNA loci was demonstrated for both coding and non-coding genes [23, 34]. Alternatively, the lower abundance of 24 nt-siRNA loci in their flanking regions could be explained considering their recent evolution and hypothesizing the necessity for a longer time to set and establish functional siRNA loci.

On the contrary an inverse correlation between genome dominance and siRNA coverage was observed in maize: genes of the recessive subgenome 2 (that are globally expressed at lower levels than those of subgenome 1) are siRNA-enriched in the upstream region, without differences between retained homeologs and single copy genes. All these data are consistent with the idea that the Pol IV-mediated RdDM pathway was involved in (and probably continues to guide) genome dominance regulation, and subgenome stability and evolution, probably balancing TEs activity through 24 nt-siRNAs production, as previously reported in *Brassica rapa* [67, 92].

## Conclusions

In this study, starting from the first total RNA-Seq analysis in maize, we extensively investigated the *rpd1/rmr6* mutant transcriptome, demonstrating that the loss of RdDM activity causes a global increase in the transcribed fraction of the maize genome. Deep transcriptome analysis were integrated with investigations on TEs distribution, smallRNA targeting and DNA methylation levels, confirming that TEs inserted near genes widely affect neighboring loci expression. The TEs effect on nearby gene expression is linked to alternative methylation profiles on gene flanking regions, and these profiles are strictly dependent on specific characteristics of the TE member inserted.

This study also indicate an involvement of the Pol IV-mediated RdDM pathway in genome dominance regulation, and subgenome stability and evolution. Given the lack of a maize mutant for MET1 orthologs (responsible for CG methylation maintenance), only the creation, if possible, of viable double mutants between CHG and CHH methylation pathway (unsuccessful up to now; [32]) could finally probe the contribution of RdDM and DNA methylation in fine balance TEs silencing with gene expression regulation and genome stability over generations in maize.

## Methods

### Plant materials, growth conditions and total RNA-Seq

The *Zea mays* B73 inbred line and the *rpd1–1* (also known as *rmr6–1*) null mutant [27], previously introgressed in B73 background by repeated backcrosses, were used for RNA-Seq and sRNA-Seq analysis. B73 inbreed seeds were obtained from the North Central Regional Plant Introduction Station (NCRPIS; USDA-ARS), while *rdp1–1/rmr6–1* mutant seed were kindly provided by University of California - UC Berkeley under a Material Transfer Agreement. Plants were grown in pots in a greenhouse during spring-summer growing seasons in 2011 and 2012, and subjected to drought (D), salinity (S) and drought plus salinity (D + S) stresses as described in [23, 40, 93]. A total of 16 leaf samples were then collected for each of the three biological replicates (R1, R2, R3) produced.

Total RNA was extracted from frozen tissue using the Spectrum Plant Total RNA Kit (SIGMA) and subjected to On-Column DNase Digestion (SIGMA), according to the manufacturer’s instructions. rRNA depletion was performed with Ribo-Zero™ rRNA Removal Kits (Plant Leaf) from Epicenter-Illumina. Libraries for Illumina sequencing were prepared with the TruSeq RNA Library Prep Kit for the first replicate (non-directional sequencing) and with the TruSeq Stranded RNA Library Prep Kit (directional sequencing) for biological replicates 2 and 3, which were pooled and sequenced together [40]. The Illumina sequencing of the 32 RNA-Seq libraries was performed at the Istituto di Genomica Applicata (Udine, Italy) on an Illumina Hiseq2000 platform with a multiplex level of 4, producing on average 37 million 50 bp single-end reads per library (Additional file 1).

### Total RNA-Seq reads alignment and genome guided transcriptome assembly

The bioinformatics analyses are fully described in Forestan et al. [40]. In brief, the sequenced reads were pre-processed for adapter clipping using Cutadapt 1.2.1 [94] and then trimmed on quality scores and filtered from rRNA contaminant reads (rRNA reference sequences were retrieved from http://www.arb-silva.de/) with ERNE-FILTER 1.2 [95]. The resulting high quality reads (Additional file 1) were mapped to the maize B73 reference genome (RefGen ZmB73 Assembly AGPv3 and Zea_mays.AGPv3.20.gtf transcript annotation) with Tophat 2.0.9 [96]. About 50–60% of the aligned reads resulted as multi-mapped (mapped equally well to two or more genomic positions): to preserve their biological information (i.e. transcription from paralogous genes and repetitive sequences) avoiding negative impact on downstream analysis, we decided to discard those mapping on more than 10 different genomic positions. Reads with MAPping Quality (MAPQ) smaller than 1 and PCR duplicates were therefore filtered out using Samtools [97], reducing the multi-mapped reads to about the 30–40% of the mapped reads (Additional file 1). As described in [40], reads from R2 + R3 sequencing, aligned in strand-specific mode, were used for genome guided transcriptome assembly with Cufflinks 2.2.1 - RABT mode - [45, 49] resulting in the identification of 3410 new transcribed loci and about 21,400 potential novel isoforms at known loci.

Transcript sequences related to transposable elements were identified and classified using BLAST Best Hits. All 160,488 transcript models (reference plus newly annotated transcripts [40]) were BLASTed against the Maize TE database containing 1526 full-length sequences of curated, non-redundant maize TEs (http://maizetedb.org/~maize/). The bit score and coverage percentage of the alignment were scored to identify TE-related transcripts with stringent criteria and classify them in two subgroups: 9766 high-confident-TEs (HC-TEs: with Bit-score > 500 and coverage >50%) and 9013 putative/relics-TEs (PR-TEs: with Bit-score > 250 or coverage >30%). Each TE-related transcript was then associated with its specific TE-family and superfamily to analyze the preferential transcription of specific TE-classes in mutant samples.

### Quantification of the transcribed fraction of the genome

Filtered read alignments (MAPQ ≥ 1) from the 16 sequenced libraries for each genotype were combined producing two BAM files that contain 438,275,238 and 458,429,084 reads for B73 and *rpd1/rmr6*, respectively (corresponding to 714,514,067 and 775,422,668 alignments). The two BAM files were normalized by random down-sampling to 700 million alignments (resulting from 430 million and 410 million reads from B73 and *rpd1/rmr6*, respectively) and then used to calculate the coverage at each genomic base position. Similarly, uniquely mapped reads (MAPQ = 50: assigned to only one position in the reference and selected using Samtools [97]) were randomly down-sampled to 290 million reads for each BAM file before the genome transcription estimation. The transcribed fractions of the maize genome in the two genotypes were then calculated with the BEDTools function “genomeCoverageBed” [98] starting from the down-scaled BAM files. Down-scaling and coverage calculation were performed in triplicate and average coverages are reported.

The transcribed fraction of the genome in the two genotypes was calculated also for each individual sample (after down-sampling at level of individual RNA-Seq libraries; Additional file 3).

### Expression quantification and differential expression analysis

Gene and transcript expression values (including known and novel annotations [40]) were quantified for each genotype (considering the different growth conditions and timepoints together) with Cuffquant and normalized with Cuffnorm [45] producing a tab-delimited matrix with normalized FPKM (fragments per kilobase per million mapped fragment) values for all the genes and transcripts (Additional file 24).

Pairwise differential expression analyses were instead obtained with Cuffdiff [45] selecting the following options: --multi-read-correct, −-compatible-hits-norm, −-dispersion-method per-condition and --library-norm-method quartile. In both B73 and *rpd1/rmr6* plants grown in control conditions, preliminary tests revealed no differentially expressed genes (or transcripts) between the two timepoints (T0 and T7, data not shown), indicating that the different plant developmental stages at the two collection points didn’t cause expression variations.

To identify Pol IV regulated genes, three pairwise differential expression analyses were performed, grouping the sequenced samples in different groups, considering the different growth conditions as replicates: a) Control-test-set (B73:C_T0 + B73:C_T7 vs *rmr6*:C_T0 + *rmr6*:C_T7); b) Stress-test-set (B73:D_T0 + B73:S_T0 + B73:S+D_T0 vs *rmr6*:D_T0 + *rmr6*:S_T0 + *rmr6*:S+D_T0); c) All-test-set (all samples B73 vs all samples *rmr6*). In addition to the typical TopHat/Cuffdiff pipeline [49], differential expression analyses were carried out on the ‘All-test-set’ using the RSEM v1.2.25 software [50] and EBSeq [51]. In this case, high quality reads from each library were separately mapped with Bowtie2 [99] against the de novo assembled transcriptome to infer normalized gene abundances that were used by EBSeq to detect differentially expressed genes. Cuffdiff and EBSeq outputs were filtered for log2 fold change ratio ≥ |2| and FDR- adjusted *p* value ≤0.05 to identify statistically differentially expressed genes.

### Distributions of gene expression and statistical analysis

Genes and transcripts with test status = NOTEST or LOWDATA in all three expression analyses performed with Cuffdiff (roughly corresponding to FPKM < 1 in all the conditions) were filtered out as not expressed and not considered in expression frequency comparisons and plots (73,925 loci lowdata, 40,457 loci expressed; 94,335 transcripts lowdata, 66,163 transcripts expressed). We decided to apply the same stringent filters to both coding and ncRNAs, even though this resulted in the losing of more than 60% of non-coding transcripts, which are normally expressed at lower levels than coding ones. Expression profiles of different gene and transcript groups were investigated and statistically analyzed as described in [46]: Kolmogorov-Smirnov non-parametric test were used to compare the distributions of gene expression between the two genotypes. For each test the maximum vertical distance observed between the two curves (D-obs), the critical distance value for the test (D-crit), the *p*-value and number of genes for which the expression were compared are reported. When both D-obs > D-stat and *p* < 0.01 the expression distributions are considered statistically different.

### Transposable elements identification and annotation in gene boundaries

Repetitive regions and TEs annotation were extracted from the RefGen ZmB73 RepeatMasked Assembly AGPv3. This redundant annotation file contains more than 5 million masked regions ranging from few base pairs to several kilobases long, so after filtering low complexity regions (“dust”) and tandem repeats (“trf”), only class I retroelement annotations longer than 1 Kb and class II DNA TEs longer than 50 bp were considered in genomic classification of annotated loci. This reduced annotation was then used to identify loci with transposon located within 1 Kb upstream of the transcription start site (−1 Kb TSS), in the gene body, or 1 Kb downstream of the transcription termination site (+1 Kb TTS) using the “intersectBed” tool [98] and custom loci annotation files with modified coordinates. Genes with TEs insertion spanning the TSS (−500/+500 TSS) were identified in the same way. A schematic representation of how genes were classified with respect to TEs is reported in Additional file 7.

The distribution plots of the differentially expressed genes along maize genome were produced as follows. The genome was divided into 100 Kbp not-overlapping windows by using Bedtools “makewindows”. For each window the total number of genes and number of up- or down-regulated genes was calculated by using BEDTools “intersectbed” and “groupby” functions [98]. The gene content of each window was normalized by dividing by the number of genes in the corresponding chromosome, while the number of differentially expressed genes of each window was divided by the gene content of the window. As control, the distribution of the up-regulated genes was compared with a random dataset of 880 genes obtained from the list of the expressed genes. More specifically, the bash function “shuffle” was used to obtain 100 groups of 880 expressed genes, each group did not contain duplicated entries. The coordinates of the genes in each group were then intersected with the 100 Kbp windows, to obtain the number of genes in each window. The average number of genes per window was then calculated among the 100 repetitions to get a statistical representation of the distribution of the “random” genes.

The association between differentially expressed genes and TE was evaluated by permutation test using the regioneR R package v1.2.0 [100]. Up- and down-regulated genes were separated and for each group the distance from TE was compared to the average distance of the other genes in the genome by permutation test (*n* = 200), the “resampleRegions” was used as randomization function, “meanDistance” as evaluation function and giving the full set of genes as “universe”. To have an adequate number of genes (in particular of down-regulated ones) that guarantee statistical power to this analysis, the differentially expressed genes used for permutation tests were obtained with Cuffdiff on the previously described “All-test-set” (log2FC > |1|, FDR < 0.05; Additional file 19).

In addition, both up- and down-regulated genes were divided into expression bins based on the distribution of their log_2_FC values. For each group of genes three bins were created: 1) log_2_FC less than the first quartile, 2) log_2_FC included in the interquartile range and 3) log_2_FC higher than the third quartile (Additional file 19). The permutation test was performed on each bin as already described to assess their distance from TEs.

In order to check the DNA methylation around the differentially expressed genes, a B73 dataset of methylation values for CpG, CHG and CHH was retrieved from a whole genome bisulfite sequencing study [62]. The coordinates of the genes were expanded to 2Kbp in both directions (1Kbp for CHH context) with BEDOPS [101] and imported, together with the methylation data, as regioneR [100] variables. Misregulated genes were divided again into three bins of expression as previously described, and for each bin a permutation test (n = 200) was performed to compare the average methylation levels of the genes in the bin with respect to the rest of the genome. The “resampleRegions” was used as randomization function, “meanInRegions” as evaluation function and giving the full set of genes as “universe”. The plots of the methylation levels across the differentially expressed genes were obtained dividing the 2Kbp upstream and downstream regions in 100 bp windows with BEDTools [98] and intersecting them with the files containing the CG/CHG/CHH methylation values across the genome. Finally the average methylation value for each window was calculated with a custom script. The same procedure was applied to extract the average methylation values across all the genes in the genome.

### Identification of differentially expressed TE families

Since TEs represent a very high proportion of maize genome and transcriptome, Pol IV-mediated TE regulation was also assessed at the level of specific TE families. High quality reads were mapped with Bowtie2 [99] against the 1526 full-length TEs consensus sequences (http://maizetedb.org/~maize/). The counts table of uniquely mapped reads for each TE accession, obtained using the “multiBamCov” tool [98], was used with edgeR package [61] for differential expression analysis. 56 TE families with a log2 fold change ratio ≥ |2| and FDR- adjusted *p* value ≤0.01 were considered as differentially expressed.

### Small RNA analysis

Maize smallRNA loci were previously identified and analyzed in [23]. In brief, high quality sRNA reads obtained from the same samples (B73 and *rpd1/rmr6* young leaves from plants subjected to drought, salinity, and drought plus salinity stresses) were used to de novo annotate the sRNA generating loci using ShortStack version 3.3 [102]. Multi-mapped reads were assigned to their most likely location on a probabilistic way, guided by uniquely mapping reads counted at each of the possible multiple positions [81].

Overlaps between the 244,050 24 nt siRNA clusters annotated in this way and the 1 Kb flanking regions of genes were calculated with the “intersectBed” BEDTools function [98]. The “coverageBed” function was instead used to calculate the presence of 24 nt siRNA loci in each individual position of gene flanking regions to produce the distribution plots of siRNA loci in flanking regions of genes belonging to different subgenome classes.

## Additional files

Additional file 1: Summary of RNA-Seq reads generated per sample and mapping statistics. Number of raw sequenced reads, high quality reads and mapped reads for each analyzed sample. Multi-mapped reads mapping on more than 10 different positions (MAPQ = 0) were filtered out and library statistics after filtering are reported too. R1 and R2 indicate replicate 1 and 2, respectively. (XLSX 16 kb)

Additional file 2: *rpd1/rmr6* mutation results in the increase of the genome transcribed fraction. Histograms summarize the RNA-Seq reads coverage on the maize genome for the B73 wild-type and *rpd1/rmr6* mutant. At a threshold of two filtered mapped RNA-Seq reads, 226,168,609 bp resulted as transcribed in *rpd1/rmr6* vs the 213,466,972 bp of B73, corresponding to an increase of 6% (A), while exclusively considering the uniquely mapped reads (B) the transcription increase is 5.4% (184,098,461 bp in *rpd1/rmr6*, 174,661,954 bp in B73). Down-scaling and coverage calculation were performed in triplicate and average coverages are reported. (TIFF 642 kb)

Additional file 3: Analysis of the genome transcribed fraction at level of individual growth conditions. The tables summarize the increase in the fraction of genome transcribed (at least two mapped reads) observed in *rpd1/rmr6* mutant compared to B73, independently calculated for each individual sample. The proportion of the genome transcribed was calculated considering either multi-mapped reads (MAPQ ≥ 1) or uniquely mapped reads (MAPQ = 50) after down-sampling at level of individual RNA-Seq library. Mean and standard deviation for the fraction of genome covered by at least two reads are reported too. (XLSX 13 kb)

Additional file 4: Results of abundance filter on annotated genes and transcripts. The maize transcriptome annotation used in this study includes 160,488 transcripts at 114,382 loci [40] that were initially filtered based on the Cuffdiff test-status (see Methods) to exclude the not expressed or too lowly expressed genes/transcripts (roughly excluding all those with FPKM < 1 in all the analyzed samples). (DOCX 16 kb)

Additional file 5: Results of abundance filter on transcripts with different coding potential. Abundance filter (based on the Cuffdiff test-status; see Methods) was applied to filter out the not expressed or too lowly expressed transcripts (roughly excluding transcripts with FPKM < 1 in all the analyzed samples). (DOCX 16 kb)

Additional file 6: Distributions of transcript expression between B73 and *rpd1/rmr6* mutant for TE-related, HC-TE transcripts. Cumulative frequency is reported for HC-TE transcripts (for transcripts with >0 FPKM) subdivided in super-families based on Blastn results (see Methods). Expression distributions are statistically different between the two genotypes exclusively for RLC - Copia and RLG - Gypsy class I retrotransposons (*P* < 0.01 by K-S test). (TIFF 1163 kb)

Additional file 7: Schematic representation of how genes were classified with respect to TEs. The “intersectBed” tool [98] was used to identify maize genes with transposon located within 1 Kb upstream of the transcription start site (A; −1 Kb TSS), in the gene body (B), or 1 Kb downstream of the transcription termination site (C; +1 Kb TTS). The same locus could be included in two or three classes when TEs resulted inserted in different gene locations. TEs inserted spanning the TSS in the −500/+500 bp range are indicated in grey in A and B. (TIFF 71 kb)

Additional file 8: TEs inserted into gene boundaries affect expression of neighboring genes in *rpd1/rmr6* mutant. Histograms of expression distribution (left) and cumulative frequency (right) for genes with >0 FPKM are reported for genes with TEs inserted within 1 kb upstream of the transcription start site (−1 kb TSS; A), within 1 kb downstream of the transcription termination site (+1 kb TTS; B), in the gene body (C) or without TE insertions (D). For the three groups of genes with nearby TE insertions the distributions of gene expression (including only genes with FPKM > 0) are statistically different between the two genotypes (P < 0.01 by Kolmogorov-Smirnov test), with higher expression in *rpd1/rmr6* mutants compared to B73 for expression values ranging from 0.5 to 10 FPKM (expression bins −1 to 3). On the contrary, genes without TE insertions (inside or nearby) show similar distribution of gene expression between genotypes. Graph (E) represents instead the average gene expression levels as a function of the distance to the nearest TE for both genotypes. Distance was binned into 500 bp windows and a distance of 0 indicates genes that contain a TE in their gene bodies. Standard errors are shown. (TIFF 1251 kb)

Additional file 9: *b1* gene overexpression in *rpd1/rmr6* mutant leaves. The *booster1* (*b1*, GRMZM2G172795) gene, which encodes a basic helix-loop-helix protein, resulted highly up-regulated in all the performed differential expression analyses, represents an hallmark of epigenetic silencing release in *rpd1/rmr6* leaves compared with wild type. (DOCX 16 kb)

Additional file 10: *rpd1/rmr6* specific targets, commonly de-regulated in at least three of the four independent analyses. (XLSX 108 kb)

Additional file 11: Summary of genes resulting misregulated in both *rpd1/rmr6* and *rdr2/mop1* maize mutants. Venn diagrams representing the genes commonly up- and down-regulated in both maize RdDM mutants. Genes differentially expressed in *rdr2/mop1* mutant were taken from [42, 43]. (TIFF 257 kb)

Additional file 12: List and annotation of genes resulting as misregulated in both *rpd1/rmr6* and *rdr2/mop1* maize mutants. (DOCX 19 kb)

Additional file 13: TE superfamilies identified amongst genes differentially expressed in *rpd1/rmr6* mutant. The number and relative percentage of up- and down-regulated genes classified as TEs (loci with at least one transcript previously classified as HC-TE or pr-TE, see Methods) categorized in superfamilies. The relative abundance of each TEs superfamily within the 114,382 annotated genes is also reported. (DOCX 18 kb)

Additional file 14: TE families differentially transcribed in *rpd1/rmr6* mutant. Differentially expressed TE families (log2FC > |2|; FDR < 0.01) identified by edgeR; for class I TEs the number of full-length elements and partial fragments were obtained from [31]. (DOCX 22 kb)

Additional file 15: Distribution plots Pol IV silenced loci along maize genome. The distribution plots of the differentially expressed genes along the ten maize chromosomes indicate the preferential co-localization of *rpd1/rmr6* de-repressed genes (left plots). The chromosomes were divided in 100Kbp not-overlapping windows and for each window the percentage of genes (with respect to the total chromosome genes; blue bars) and of over-expressed genes (with respect to the window gene content) are reported. Yellow and red bars depict the window percentage of up-regulated genes shared in at least three or two independent comparisons, respectively. As control, the distribution of 880 genes randomly selected from the list of 40,457 expressed genes is reported as green bars (plots on the right). The two distributions resulted strongly statistically different (*P* = 1 × 10^−16^, by Wilcoxon test), with the random genes uniformly distributed along the genome (they resulted included in 876 genome 100 Kb windows versus the 737 including the up-regulated genes). (PDF 1141 kb)

Additional file 16: Distribution plots along maize genome of genes down-regulated in rpd1/rmr6 mutant leaves. The distribution plots of the 71 *rpd1/rmr6* down-regulated genes show they are included in 67 independent genome windows uniformly distributed along the maize chromosomes. The chromosomes were divided in 100Kbp not-overlapping windows and for each window the percentage of genes (with respect to the total chromosome genes; blue bars) and of down-regulate genes (with respect to the window gene content; red bars) are reported. (TIFF 2873 kb)

Additional file 17: Genome browser view of RNA-Seq reads mapped at *rpd1/rmr6* misregulated gene cluster. Genome browser (IGV - Integrative Genomics Viewer; http://software.broadinstitute.org/software/igv/) views of B73 (red) and *rpd1/rmr6–1* mutant (blue) RNA-Seq reads (normalized to the total of mapped reads) over three examples of large chromosomal regions de-repressed in *rpd1/rmr6* mutant. Pol IV transcriptional release could interest one (A, C) or both strands (B) and interests several genes in TE-rich regions. Total mapped reads (replicates 1 and 2) and strand-specific mapped reads (replicate 2) are reported. (TIFF 1222 kb)

Additional file 18: Elaborated and raw results of permutation test on association between differentially expressed genes and TEs. Permutation test using the regioneR R package [100] revealed that up-regulated genes are significantly closer to TEs (average distance: 1550 bp) than the average of genes in the genome (permutation evaluated average distance: 2017 bp; *P* < 0.005), while down-regulated genes resulted more distant (2374 bp; P < 0.005). Graph in (A) was produced by combining the single graphs reported in (B) and (C), obtained from the independent analysis of up- and down-regulated genes. Differentially expressed genes for this analysis (log2FC > |1|, FDR < 0.05) were obtained with Cuffdiff starting from all the sequenced samples. (TIFF 600 kb)

Additional file 19: *rpd1/rmr6* differentially expressed genes (log2FC > |1|, FDR < 0.05) obtained with Cuffdiff, starting from all the sequenced samples, subdivided into bins based on their fold change variation. (XLSX 357 kb)

Additional file 20: DNA methylation profiles at differentially expressed gene flanking regions. Methylation levels in each context (CG, CHG and CHH) were computed for the flanking regions (2 Kb for CG and CHG, 1 Kb for CHH; see Methods) of differentially expressed genes. Genes were divided in bins according to fold change variation, and methylation levels of each bin were compared to the average of genes in the genome. (TIFF 1402 kb)

Additional file 21: Summary of methylation levels at differentially expressed genes flanking regions obtained by permutation analysis. Methylation levels in each context (CG, CHG and CHH) were computed independently for the flanking regions (2 Kb for CG and CHG, 1 Kb for CHH; see Methods) of differentially expressed genes and compared to the average of genes in the genome using regioneR permutation approach [100]. Genes were divided into bins accordingly to fold change expression variation in *rpd1/rmr6* mutant compared to wild-type (Additional file 19). For each bin the average methylation value is associated to the *p*-value obtained by permutation analysis, value that summarizes the statistical significance divergence between bin average methylation level and the average of annotated genes in the genome. Red and blue values indicate statistically higher and lower methylation levels, respectively, compared to the whole gene set. (DOCX 19 kb)

Additional file 22: Expression of subgenome 1 and subgenome 2 assigned genes in *rpd1/rmr6* mutant and B73 wild-type plants. Differentially expressed gene models (log2FC > |1|, FDR < 0.05) ascribable to singletons and duplicates in the maize subgenomes or non-syntenic genes were obtained with Cuffdiff starting from all the sequenced samples. (DOCX 17 kb)

Additional file 23: Distribution plots of siRNA loci occupancy. The plots of siRNA loci coverage in flanking regions of subgenome genes, further split between homeologs and single copy genes of each subgenome, confirm that genes of the recessive subgenome 2 are preferentially siRNA-enriched in the upstream region, without differences between retained homeologs and single copy genes. (TIFF 395 kb)

Additional file 24: Matrix with normalized FPKM (fragments per kilobase per million mapped fragment) values for all the annotated genes and transcripts. (XLSX 11865 kb)

## Abbreviations

RdDM: RNA-directed DNA methylation
siRNA: small interfering RNAs
TEs: transposable elements

## References

[1] Matzke MA, Mosher RA (2014). RNA-directed DNA methylation: an epigenetic pathway of increasing complexity. Nat Rev Genet.

[2] Pikaard CS, Haag JR, Pontes OM, Blevins T, Cocklin R (2012). A transcription fork model for Pol IV and Pol V-dependent RNA-directed DNA methylation. Cold Spring Harb Symp Quant Biol.

[3] Wierzbicki AT (2012). The role of long non-coding RNA in transcriptional gene silencing. Curr Opin Plant Biol.

[4] Zhang H, Zhu JK (2011). RNA-directed DNA methylation. Curr Opin Plant Biol.

[5] Zilberman D, Cao X, Jacobsen SE (2003). ARGONAUTE4 control of locus-specific siRNA accumulation and DNA and histone methylation. Science.

[6] Meister G (2013). Argonaute proteins: functional insights and emerging roles. Nat Rev Genet.

[7] Kim MY, Zilberman D (2014). DNA methylation as a system of plant genomic immunity. Trends Plant Sci.

[8] Herr AJ, Jensen MB, Dalmay T, Baulcombe DC (2005). RNA polymerase IV directs silencing of endogenous DNA. Science.

[9] Onodera Y, Haag JR, Ream T, Costa Nunes P, Pontes O, Pikaard CS (2005). Plant nuclear RNA polymerase IV mediates siRNA and DNA methylation-dependent heterochromatin formation. Cell.

[10] Kanno T, Huettel B, Mette MF, Aufsatz W, Jaligot E, Daxinger L, Kreil DP, Matzke M, Matzke AJ (2005). Atypical RNA polymerase subunits required for RNA-directed DNA methylation. Nat Genet.

[11] Pontier D, Yahubyan G, Vega D, Bulski A, Saez-Vasquez J, Hakimi MA, Lerbs-Mache S, Colot V, Lagrange T (2005). Reinforcement of silencing at transposons and highly repeated sequences requires the concerted action of two distinct RNA polymerases IV in Arabidopsis. Genes Dev.

[12] Xie Z, Johansen LK, Gustafson AM, Kasschau KD, Lellis AD, Zilberman D, Jacobsen SE, Carrington JC (2004). Genetic and functional diversification of small RNA pathways in plants. PLoS Biol.

[13] Haag JR, Brower-Toland B, Krieger EK, Sidorenko L, Nicora CD, Norbeck AD, Irsigler A, LaRue H, Brzeski J, McGinnis K, Ivashuta S, Pasa-Tolic L, Chandler VL, Pikaard CS (2014). Functional diversification of maize RNA polymerase IV and V subtypes via alternative catalytic subunits. Cell Rep.

[14] Haag JR, Ream TS, Marasco M, Nicora CD, Norbeck AD, Pasa-Tolic L, Pikaard CS (2012). In vitro transcription activities of Pol IV, Pol V, and RDR2 reveal coupling of Pol IV and RDR2 for dsRNA synthesis in plant RNA silencing. Mol Cell.

[15] Law JA, Vashisht AA, Wohlschlegel JA, Jacobsen SE (2011). SHH1, a homeodomain protein required for DNA methylation, as well as RDR2, RDM4, and chromatin remodeling factors, associate with RNA polymerase IV. PLoS Genet.

[16] Blevins T, Podicheti R, Mishra V, Marasco M, Tang H, Pikaard CS. Identification of Pol IV and RDR2-dependent precursors of 24 nt siRNAs guiding de novo DNA methylation in Arabidopsis. elife. 2015;4 10.7554/eLife.09591. 10.7554/eLife.09591 PMC4716838 26430765

[17] Zhai J, Bischof S, Wang H, Feng S, Lee TF, Teng C, Chen X, Park SY, Liu L, Gallego-Bartolome J, Liu W, Henderson IR, Meyers BC, Ausin I, Jacobsen SE (2015). A one precursor one siRNA model for Pol IV-dependent siRNA biogenesis. Cell.

[18] Mosher RA, Schwach F, Studholme D, Baulcombe DC (2008). PolIVb influences RNA-directed DNA methylation independently of its role in siRNA biogenesis. Proc Natl Acad Sci U S A.

[19] Wierzbicki AT, Haag JR, Pikaard CS (2008). Noncoding transcription by RNA polymerase Pol IVb/Pol V mediates transcriptional silencing of overlapping and adjacent genes. Cell.

[20] Wierzbicki AT, Ream TS, Haag JR, Pikaard CS (2009). RNA polymerase V transcription guides ARGONAUTE4 to chromatin. Nat Genet.

[21] Law JA, Du J, Hale CJ, Feng S, Krajewski K, Palanca AM, Strahl BD, Patel DJ, Jacobsen SE (2013). Polymerase IV occupancy at RNA-directed DNA methylation sites requires SHH1. Nature.

[22] Zhang X, Henderson IR, Lu C, Green PJ, Jacobsen SE (2007). Role of RNA polymerase IV in plant small RNA metabolism. Proc Natl Acad Sci U S A.

[23] Lunardon A, Forestan C, Farinati S, Axtell M, Varotto S (2016). Genome-wide characterization of maize small RNA loci and their regulation in the required to maintain repression6-1 (rmr6-1) mutant and long-term abiotic stresses. Plant Physiol.

[24] Nobuta K, Lu C, Shrivastava R, Pillay M, De Paoli E, Accerbi M, Arteaga-Vazquez M, Sidorenko L, Jeong DH, Yen Y, Green PJ, Chandler VL, Meyers BC (2008). Distinct size distribution of endogeneous siRNAs in maize: evidence from deep sequencing in the mop1-1 mutant. Proc Natl Acad Sci U S A.

[25] Gent JI, Madzima TF, Bader R, Kent MR, Zhang X, Stam M, McGinnis KM, Dawe RK (2014). Accessible DNA and relative depletion of H3K9me2 at maize loci undergoing RNA-directed DNA methylation. Plant Cell.

[26] Huang Y, Kendall T, Mosher RA (2013). Pol IV-dependent siRNA production is reduced in Brassica Rapa. Biology (Basel).

[27] Erhard KF, Stonaker JL, Parkinson SE, Lim JP, Hale CJ, Hollick JB (2009). RNA polymerase IV functions in Paramutation in Zea Mays. Science.

[28] Parkinson SE, Gross SM, Hollick JB (2007). Maize sex determination and abaxial leaf fates are canalized by a factor that maintains repressed epigenetic states. Dev Biol.

[29] Arabidopsis Genome Initiative (2000). Analysis of the genome sequence of the flowering plant Arabidopsis Thaliana. Nature.

[30] Schnable PS, Ware D, Fulton RS, Stein JC, Wei F, Pasternak S, Liang C, Zhang J, Fulton L, Graves TA, Minx P, Reily AD, Courtney L, Kruchowski SS, Tomlinson C, Strong C, Delehaunty K, Fronick C, Courtney B, Rock SM, Belter E, Du F, Kim K, Abbott RM, Cotton M, Levy A, Marchetto P, Ochoa K, Jackson SM, Gillam B, Chen W, Yan L, Higginbotham J, Cardenas M, Waligorski J, Applebaum E, Phelps L, Falcone J, Kanchi K, Thane T, Scimone A, Thane N, Henke J, Wang T, Ruppert J, Shah N, Rotter K, Hodges J, Ingenthron E, Cordes M, Kohlberg S, Sgro J, Delgado B, Mead K, Chinwalla A, Leonard S, Crouse K, Collura K, Kudrna D, Currie J, He R, Angelova A, Rajasekar S, Mueller T, Lomeli R, Scara G, Ko A, Delaney K, Wissotski M, Lopez G, Campos D, Braidotti M, Ashley E, Golser W, Kim H, Lee S, Lin J, Dujmic Z, Kim W, Talag J, Zuccolo A, Fan C, Sebastian A, Kramer M, Spiegel L, Nascimento L, Zutavern T, Miller B, Ambroise C, Muller S, Spooner W, Narechania A, Ren L, Wei S, Kumari S, Faga B, Levy MJ, McMahan L, Van Buren P, Vaughn MW, Ying K, Yeh CT, Emrich SJ, Jia Y, Kalyanaraman A, Hsia AP, Barbazuk WB, Baucom RS, Brutnell TP, Carpita NC, Chaparro C, Chia JM, Deragon JM, Estill JC, Fu Y, Jeddeloh JA, Han Y, Lee H, Li P, Lisch DR, Liu S, Liu Z, Nagel DH, McCann MC, SanMiguel P, Myers AM, Nettleton D, Nguyen J, Penning BW, Ponnala L, Schneider KL, Schwartz DC, Sharma A, Soderlund C, Springer NM, Sun Q, Wang H, Waterman M, Westerman R, Wolfgruber TK, Yang L, Yu Y, Zhang L, Zhou S, Zhu Q, Bennetzen JL, Dawe RK, Jiang J, Jiang N, Presting GG, Wessler SR, Aluru S, Martienssen RA, Clifton SW, McCombie WR, Wing RA, Wilson RK (2009). The B73 maize genome: complexity, diversity, and dynamics. Science.

[31] Baucom RS, Estill JC, Chaparro C, Upshaw N, Jogi A, Deragon JM, Westerman RP, Sanmiguel PJ, Bennetzen JL (2009). Exceptional diversity, non-random distribution, and rapid evolution of retroelements in the B73 maize genome. PLoS Genet.

[32] Li Q, Eichten SR, Hermanson PJ, Zaunbrecher VM, Song J, Wendt J, Rosenbaum H, Madzima TF, Sloan AE, Huang J, Burgess DL, Richmond TA, McGinnis KM, Meeley RB, Danilevskaya ON, Vaughn MW, Kaeppler SM, Jeddeloh JA, Springer NM (2014). Genetic perturbation of the maize methylome. Plant Cell.

[33] Li Q, Gent JI, Zynda G, Song J, Makarevitch I, Hirsch CD, Hirsch CN, Dawe RK, Madzima TF, McGinnis KM, Lisch D, Schmitz RJ, Vaughn MW, Springer NM (2015). RNA-directed DNA methylation enforces boundaries between heterochromatin and euchromatin in the maize genome. Proc Natl Acad Sci U S A.

[34] Gent JI, Ellis NA, Guo L, Harkess AE, Yao Y, Zhang X, Dawe RK (2013). CHH islands: de novo DNA methylation in near-gene chromatin regulation in maize. Genome Res.

[35] McClintock B (1956). Controlling elements and the gene. Cold Spring Harb Symp Quant Biol.

[36] Slotkin RK, Martienssen R (2007). Transposable elements and the epigenetic regulation of the genome. Nat Rev Genet.

[37] Lisch D (2013). How important are transposons for plant evolution?. Nat Rev Genet.

[38] Cowley M, Oakey RJ (2013). Transposable elements re-wire and fine-tune the transcriptome. PLoS Genet.

[39] Makarevitch I, Waters AJ, West PT, Stitzer M, Hirsch CN, Ross-Ibarra J, Springer NM (2015). Transposable elements contribute to activation of maize genes in response to abiotic stress. PLoS Genet.

[40] Forestan C, Aiese Cigliano R, Farinati S, Lunardon A, Sanseverino W, Varotto S (2016). Stress-induced and epigenetic-mediated maize transcriptome regulation study by means of transcriptome reannotation and differential expression analysis. Sci Rep.

[41] Erhard KF, Talbot JE, Deans NC, AE MC, Hollick JB (2015). Nascent transcription affected by RNA polymerase IV in Zea Mays. Genetics.

[42] Madzima TF, Huang J, McGinnis KM (2014). Chromatin structure and gene expression changes associated with loss of MOP1 activity in Zea Mays. Epigenetics.

[43] Jia Y, Lisch DR, Ohtsu K, Scanlon MJ, Nettleton D, Schnable PS (2009). Loss of RNA-dependent RNA polymerase 2 (RDR2) function causes widespread and unexpected changes in the expression of transposons, genes, and 24-nt small RNAs. PLoS Genet.

[44] Matzke MA, Kanno T, Matzke AJ (2015). RNA-directed DNA Methylation: the evolution of a complex epigenetic pathway in flowering plants. Annu Rev Plant Biol.

[45] Trapnell C, Hendrickson DG, Sauvageau M, Goff L, Rinn JL, Pachter L (2013). Differential analysis of gene regulation at transcript resolution with RNA-seq. Nat Biotechnol.

[46] Deng X, Hiatt JB, Nguyen DK, Ercan S, Sturgill D, Hillier LW, Schlesinger F, Davis CA, Reinke VJ, Gingeras TR, Shendure J, Waterston RH, Oliver B, Lieb JD, Disteche CM (2011). Evidence for compensatory upregulation of expressed X-linked genes in mammals, Caenorhabditis Elegans and Drosophila Melanogaster. Nat Genet.

[47] Blevins T, Pontvianne F, Cocklin R, Podicheti R, Chandrasekhara C, Yerneni S, Braun C, Lee B, Rusch D, Mockaitis K, Tang H, Pikaard CS (2014). A two-step process for epigenetic inheritance in Arabidopsis. Mol Cell.

[48] Conesa A, Madrigal P, Tarazona S, Gomez-Cabrero D, Cervera A, McPherson A, Szczesniak MW, Gaffney DJ, Elo LL, Zhang X, Mortazavi A (2016). A survey of best practices for RNA-seq data analysis. Genome Biol.

[49] Trapnell C, Roberts A, Goff L, Pertea G, Kim D, Kelley DR, Pimentel H, Salzberg SL, Rinn JL, Pachter L (2012). Differential gene and transcript expression analysis of RNA-seq experiments with TopHat and cufflinks. Nat Protoc.

[50] Li B, Dewey CN (2011). RSEM: accurate transcript quantification from RNA-Seq data with or without a reference genome. BMC Bioinformatics.

[51] Leng N, Dawson JA, Thomson JA, Ruotti V, Rissman AI, Smits BM, Haag JD, Gould MN, Stewart RM, Kendziorski C (2013). EBSeq: an empirical Bayes hierarchical model for inference in RNA-seq experiments. Bioinformatics.

[52] Hollick JB, Kermicle JL, Parkinson SE (2005). Rmr6 maintains meiotic inheritance of paramutant states in Zea Mays. Genetics.

[53] Danilevskaya ON, Meng X, Selinger DA, Deschamps S, Hermon P, Vansant G, Gupta R, Ananiev EV, Muszynski MG (2008). Involvement of the MADS-box gene ZMM4 in floral induction and inflorescence development in maize. Plant Physiol.

[54] Walsh J, Waters CA, Freeling M (1998). The maize gene liguleless2 encodes a basic leucine zipper protein involved in the establishment of the leaf blade-sheath boundary. Genes Dev.

[55] Walsh J, Freeling M (1999). The liguleless2 gene of maize functions during the transition from the vegetative to the reproductive shoot apex. Plant J.

[56] Zhang X, Liu S, Takano T (2008). Two cysteine proteinase inhibitors from Arabidopsis Thaliana, AtCYSa and AtCYSb, increasing the salt, drought, oxidation and cold tolerance. Plant Mol Biol.

[57] Hwang JE, Hong JK, Lim CJ, Chen H, Je J, Yang KA, Kim DY, Choi YJ, Lee SY, Lim CO (2010). Distinct expression patterns of two Arabidopsis phytocystatin genes, AtCYS1 and AtCYS2, during development and abiotic stresses. Plant Cell Rep.

[58] Morales-Ruiz T, Ortega-Galisteo AP, Ponferrada-Marin MI, Martinez-Macias MI, Ariza RR, Roldan-Arjona T (2006). DEMETER and REPRESSOR OF SILENCING 1 encode 5-methylcytosine DNA glycosylases. Proc Natl Acad Sci U S A.

[59] Penterman J, Zilberman D, Huh JH, Ballinger T, Henikoff S, Fischer RL (2007). DNA demethylation in the Arabidopsis genome. Proc Natl Acad Sci U S A.

[60] Williams BP, Pignatta D, Henikoff S, Gehring M (2015). Methylation-sensitive expression of a DNA demethylase gene serves as an epigenetic rheostat. PLoS Genet.

[61] Robinson MD, McCarthy DJ, Smyth GK (2010). edgeR: a bioconductor package for differential expression analysis of digital gene expression data. Bioinformatics.

[62] Regulski M, Lu Z, Kendall J, Donoghue MT, Reinders J, Llaca V, Deschamps S, Smith A, Levy D, McCombie WR, Tingey S, Rafalski A, Hicks J, Ware D, Martienssen RA (2013). The maize methylome influences mRNA splice sites and reveals widespread paramutation-like switches guided by small RNA. Genome Res.

[63] Blanc G, Wolfe KH (2004). Widespread paleopolyploidy in model plant species inferred from age distributions of duplicate genes. Plant Cell.

[64] Swigonova Z, Lai J, Ma J, Ramakrishna W, Llaca V, Bennetzen JL, Messing J (2004). Close split of sorghum and maize genome progenitors. Genome Res.

[65] Schnable JC, Springer NM, Freeling M (2011). Differentiation of the maize subgenomes by genome dominance and both ancient and ongoing gene loss. Proc Natl Acad Sci U S A.

[66] Woodhouse MR, Schnable JC, Pedersen BS, Lyons E, Lisch D, Subramaniam S, Freeling M (2010). Following tetraploidy in maize, a short deletion mechanism removed genes preferentially from one of the two homologs. PLoS Biol.

[67] Woodhouse MR, Cheng F, Pires JC, Lisch D, Freeling M, Wang X (2014). Origin, inheritance, and gene regulatory consequences of genome dominance in polyploids. Proc Natl Acad Sci U S A.

[68] Freeling M, Lyons E, Pedersen B, Alam M, Ming R, Lisch D (2008). Many or most genes in Arabidopsis transposed after the origin of the order Brassicales. Genome Res.

[69] Barbaglia AM, Klusman KM, Higgins J, Shaw JR, Hannah LC, Lal SK (2012). Gene capture by Helitron transposons reshuffles the transcriptome of maize. Genetics.

[70] Nuthikattu S, McCue AD, Panda K, Fultz D, DeFraia C, Thomas EN, Slotkin RK (2013). The initiation of epigenetic silencing of active transposable elements is triggered by RDR6 and 21-22 nucleotide small interfering RNAs. Plant Physiol.

[71] Panda K, Slotkin RK: Proposed mechanism for the initiation of transposable element silencing by the RDR6-directed DNA methylation pathway. Plant Signal Behav 2013, 8(8)**;**10.4161/psb.25206. Epub 2013 Jun 5. 10.4161/psb.25206 PMC3999056 23759554

[72] McCue AD, Panda K, Nuthikattu S, Choudury SG, Thomas EN, Slotkin RK (2015). ARGONAUTE 6 bridges transposable element mRNA-derived siRNAs to the establishment of DNA methylation. EMBO J.

[73] Lippman Z, Gendrel AV, Black M, Vaughn MW, Dedhia N, McCombie WR, Lavine K, Mittal V, May B, Kasschau KD, Carrington JC, Doerge RW, Colot V, Martienssen R (2004). Role of transposable elements in heterochromatin and epigenetic control. Nature.

[74] Hollister JD, Gaut BS (2009). Epigenetic silencing of transposable elements: a trade-off between reduced transposition and deleterious effects on neighboring gene expression. Genome Res.

[75] Zhang X, Shiu SH, Cal A, Borevitz JO (2008). Global analysis of genetic, epigenetic and transcriptional polymorphisms in Arabidopsis Thaliana using whole genome tiling arrays. PLoS Genet.

[76] Huettel B, Kanno T, Daxinger L, Aufsatz W, Matzke AJ, Matzke M (2006). Endogenous targets of RNA-directed DNA methylation and Pol IV in Arabidopsis. EMBO J.

[77] Tran RK, Zilberman D, de Bustos C, Ditt RF, Henikoff JG, Lindroth AM, Delrow J, Boyle T, Kwong S, Bryson TD, Jacobsen SE, Henikoff S (2005). Chromatin and siRNA pathways cooperate to maintain DNA methylation of small transposable elements in Arabidopsis. Genome Biol.

[78] Zhong X, Hale CJ, Law JA, Johnson LM, Feng S, Tu A, Jacobsen SE (2012). DDR complex facilitates global association of RNA polymerase V to promoters and evolutionarily young transposons. Nat Struct Mol Biol.

[79] Lee TF, Gurazada SG, Zhai J, Li S, Simon SA, Matzke MA, Chen X, Meyers BC (2012). RNA polymerase V-dependent small RNAs in Arabidopsis originate from small, intergenic loci including most SINE repeats. Epigenetics.

[80] Wang X, Weigel D, Smith LM (2013). Transposon variants and their effects on gene expression in Arabidopsis. PLoS Genet.

[81] Johnson NR, Yeoh JM, Coruh C, Axtell MJ (2016). Improved placement of multi-mapping small RNAs. G3 (Bethesda).

[82] You W, Lorkovic ZJ, Matzke AJ, Matzke M (2013). Interplay among RNA polymerases II, IV and V in RNA-directed DNA methylation at a low copy transgene locus in Arabidopsis Thaliana. Plant Mol Biol.

[83] Greaves IK, Groszmann M, Ying H, Taylor JM, Peacock WJ, Dennis ES (2012). Trans chromosomal methylation in Arabidopsis hybrids. Proc Natl Acad Sci U S A.

[84] West PT, Li Q, Ji L, Eichten SR, Song J, Vaughn MW, Schmitz RJ, Springer NM (2014). Genomic distribution of H3K9me2 and DNA methylation in a maize genome. PLoS One.

[85] Eichten SR, Ellis NA, Makarevitch I, Yeh CT, Gent JI, Guo L, McGinnis KM, Zhang X, Schnable PS, Vaughn MW, Dawe RK, Springer NM (2012). Spreading of heterochromatin is limited to specific families of maize retrotransposons. PLoS Genet.

[86] Chettoor AM, Givan SA, Cole RA, Coker CT, Unger-Wallace E, Vejlupkova Z, Vollbrecht E, Fowler JE, Evans MM (2014). Discovery of novel transcripts and gametophytic functions via RNA-seq analysis of maize gametophytic transcriptomes. Genome Biol.

[87] Slotkin RK, Vaughn M, Borges F, Tanurdzic M, Becker JD, Feijo JA, Martienssen RA (2009). Epigenetic reprogramming and small RNA silencing of transposable elements in pollen. Cell.

[88] Calarco JP, Borges F, Donoghue MT, Van Ex F, Jullien PE, Lopes T, Gardner R, Berger F, Feijo JA, Becker JD, Martienssen RA (2012). Reprogramming of DNA methylation in pollen guides epigenetic inheritance via small RNA. Cell.

[89] He H, Yang T, Wu W, Zheng B (2015). Small RNAs in pollen. Sci China Life Sci.

[90] Kersting AR, Bornberg-Bauer E, Moore AD, Grath S (2012). Dynamics and adaptive benefits of protein domain emergence and arrangements during plant genome evolution. Genome Biol Evol.

[91] Paschold A, Larson NB, Marcon C, Schnable JC, Yeh CT, Lanz C, Nettleton D, Piepho HP, Schnable PS, Hochholdinger F (2014). Nonsyntenic genes drive highly dynamic complementation of gene expression in maize hybrids. Plant Cell.

[92] Freeling M, Xu J, Woodhouse M, Lisch D (2015). A solution to the C-value paradox and the function of junk DNA: the genome balance hypothesis. Mol Plant.

[93] Morari F, Meggio F, Lunardon A, Scudiero E, Forestan C, Farinati S, Varotto S (2015). Time course of biochemical, physiological, and molecular responses to field-mimicked conditions of drought, salinity, and recovery in two maize lines. Front Plant Sci.

[94] Martin M (2011). Cutadapt removes adapter sequences from high-throughput sequencing reads. EMBnet Journal.

[95] Del Fabbro C, Scalabrin S, Morgante M, Giorgi FM (2013). An extensive evaluation of read trimming effects on Illumina NGS data analysis. PLoS One.

[96] Kim D, Pertea G, Trapnell C, Pimentel H, Kelley R, Salzberg SL (2013). TopHat2: accurate alignment of transcriptomes in the presence of insertions, deletions and gene fusions. Genome Biol.

[97] Li H, Handsaker B, Wysoker A, Fennell T, Ruan J, Homer N, Marth G, Abecasis G, Durbin R (2009). 1000 genome project data processing subgroup: the sequence alignment/map format and SAMtools. Bioinformatics.

[98] Quinlan AR, Hall IM (2010). BEDTools: a flexible suite of utilities for comparing genomic features. Bioinformatics.

[99] Langmead B, Salzberg SL (2012). Fast gapped-read alignment with bowtie 2. Nat Methods.

[100] Gel B, Diez-Villanueva A, Serra E, Buschbeck M, Peinado MA (2016). Malinverni R: regioneR: an R/bioconductor package for the association analysis of genomic regions based on permutation tests. Bioinformatics.

[101] Neph S, Reynolds AP, Kuehn MS, Stamatoyannopoulos JA (2016). Operating on genomic ranges using BEDOPS. Methods Mol Biol.

[102] Axtell MJ (2013). ShortStack: comprehensive annotation and quantification of small RNA genes. RNA.

