# Supplementary figures and images for: Maize RNA PolIV affects the expression of genes with nearby TE insertions and has a genome-wide repressive impact on transcription
Source: BMC Plant Biol. 2017 Oct 12;17:161. doi: 10.1186/s12870-017-1108-1 (PMC5639751; doi:10.1186/s12870-017-1108-1)

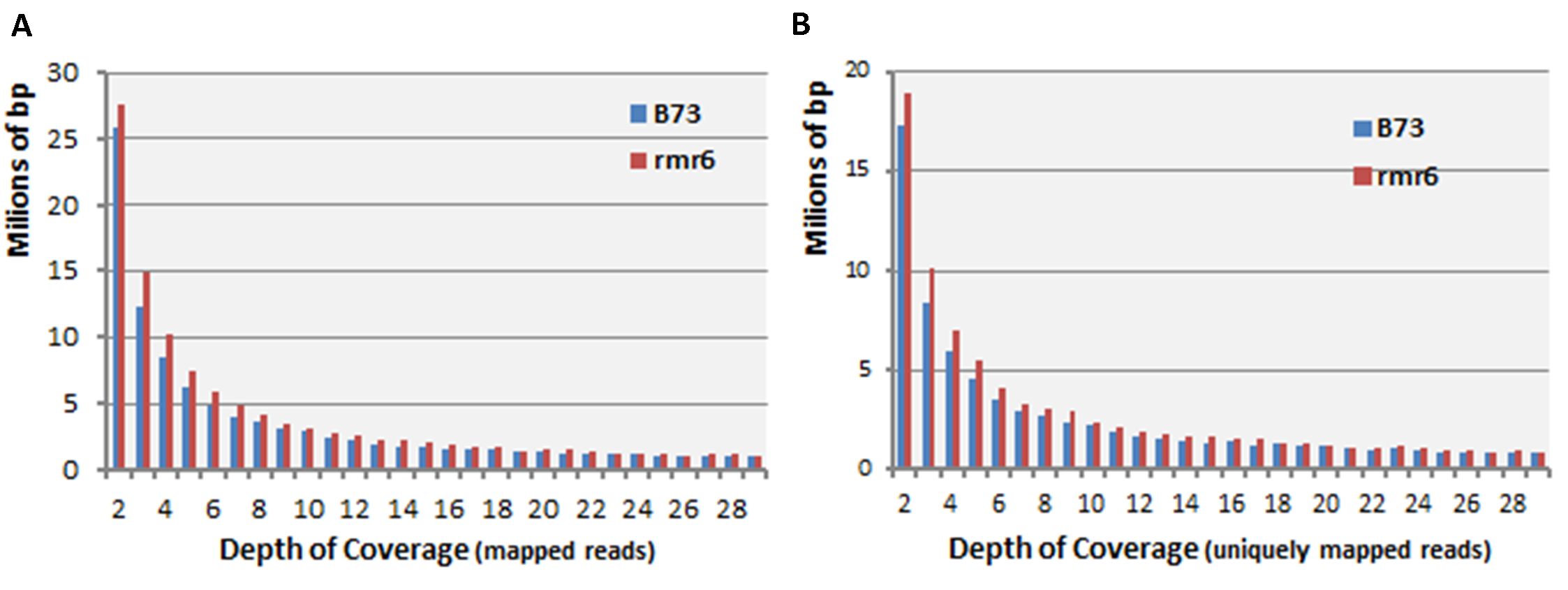

Supplement: Supplementary file 2 — rpd1/rmr6 mutation results in the increase of the genome transcribed fraction. Histograms summarize the RNA-Seq reads coverage on the maize genome for the B73 wild-type and rpd1/rmr6 mutant. At a threshold of two filtered mapped RNA-Seq reads, 226,168,609 bp resulted as transcribed in rpd1/rmr6 vs the 213,466,972 bp of B73, corresponding to an increase of 6% (A), while exclusively considering the uniquely mapped reads (B) the transcription increase is 5.4% (184,098,461 bp in rpd1/rmr6, 174,661,954 bp in B73). Down-scaling and coverage calculation were performed in triplicate and average coverages are reported. (TIFF 642 kb) [file 12870_2017_1108_MOESM2_ESM.tif]

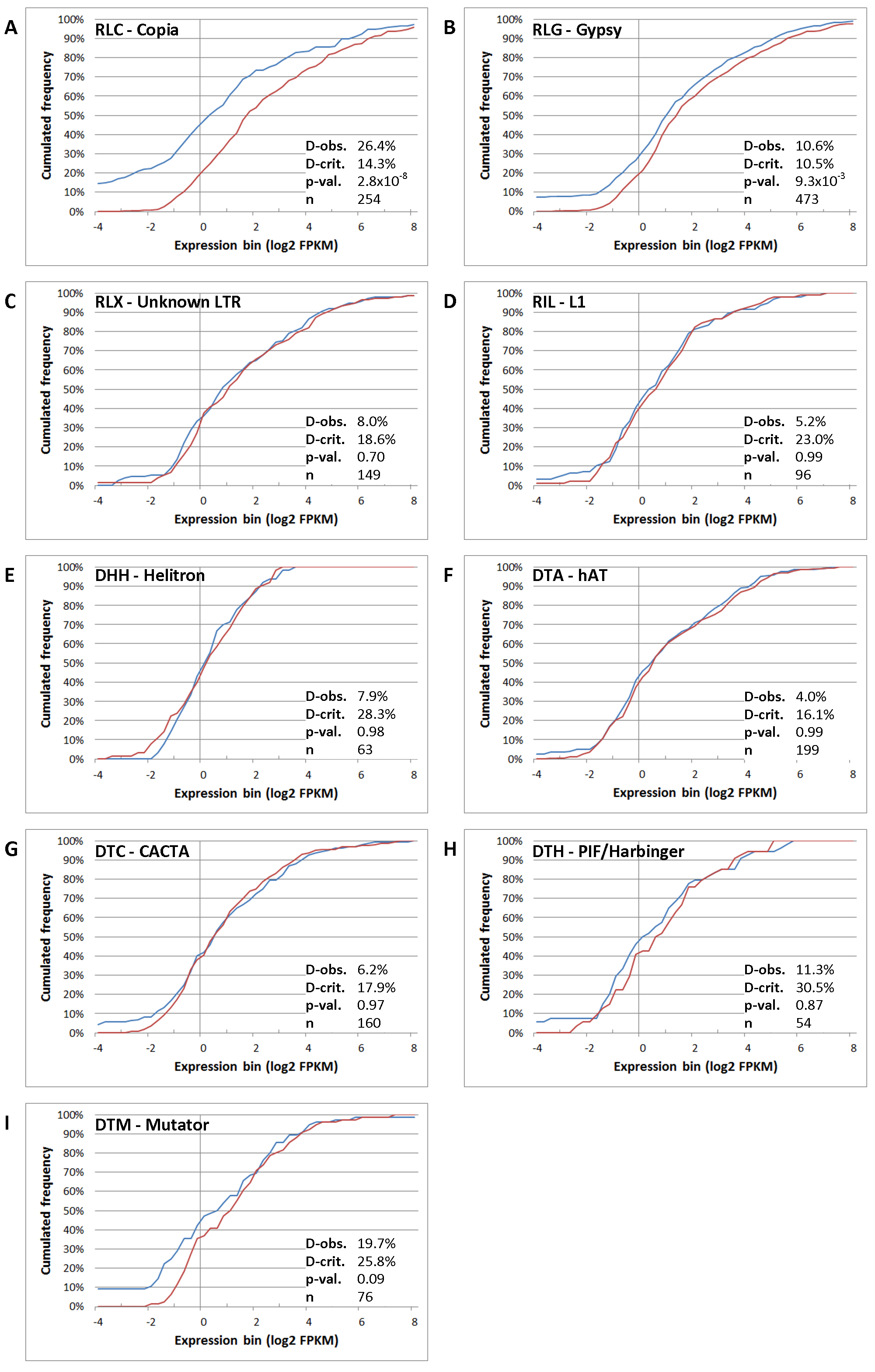

Supplement: Supplementary file 6 — Distributions of transcript expression between B73 and rpd1/rmr6 mutant for TE-related, HC-TE transcripts. Cumulative frequency is reported for HC-TE transcripts (for transcripts with >0 FPKM) subdivided in super-families based on Blastn results (see Methods). Expression distributions are statistically different between the two genotypes exclusively for RLC - Copia and RLG - Gypsy class I retrotransposons (P < 0.01 by K-S test). (TIFF 1163 kb) [file 12870_2017_1108_MOESM6_ESM.tif]

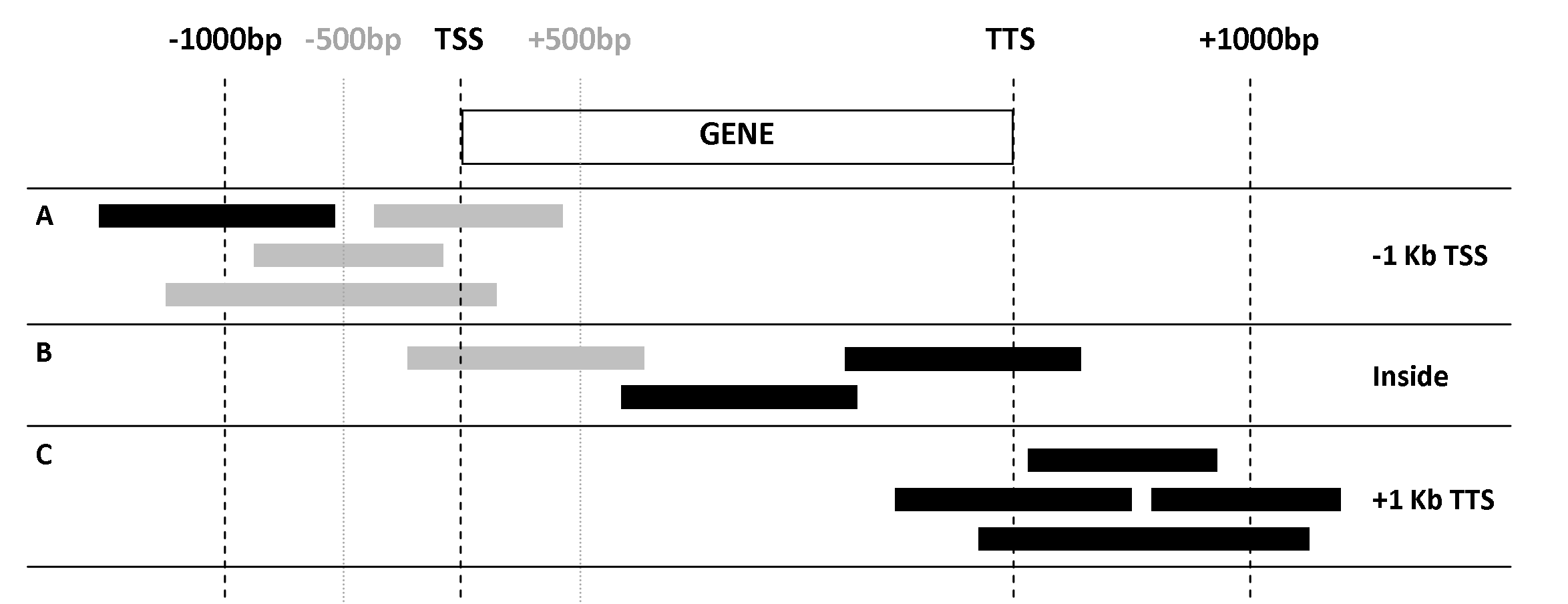

Supplement: Supplementary file 7 — Schematic representation of how genes were classified with respect to TEs. The “intersectBed” tool [98] was used to identify maize genes with transposon located within 1 Kb upstream of the transcription start site (A; −1 Kb TSS), in the gene body (B), or 1 Kb downstream of the transcription termination site (C; +1 Kb TTS). The same locus could be included in two or three classes when TEs resulted inserted in different gene locations. TEs inserted spanning the TSS in the −500/+500 bp range are indicated in grey in A and B. (TIFF 71 kb) [file 12870_2017_1108_MOESM7_ESM.tif]

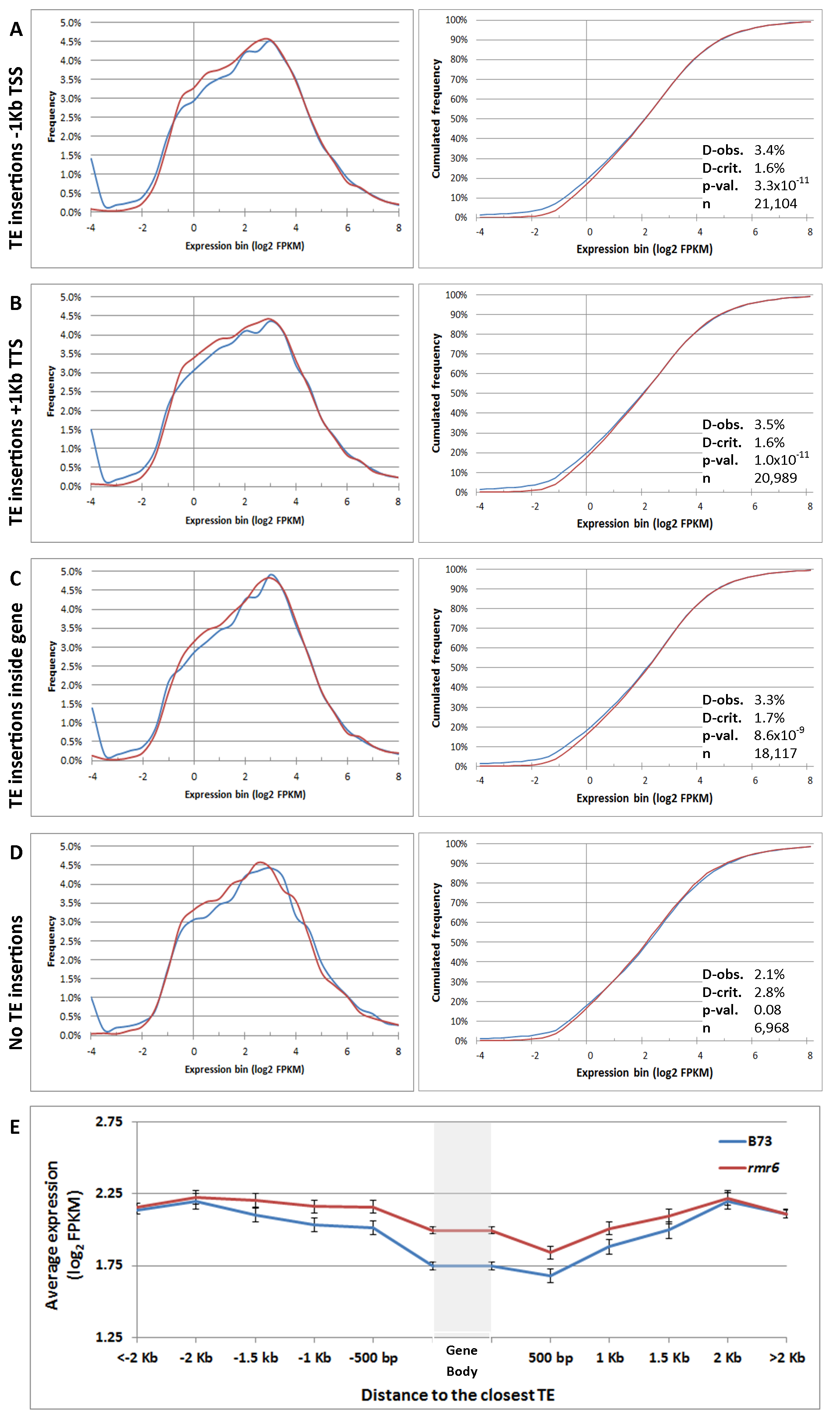

Supplement: Supplementary file 8 — TEs inserted into gene boundaries affect expression of neighboring genes in rpd1/rmr6 mutant. Histograms of expression distribution (left) and cumulative frequency (right) for genes with >0 FPKM are reported for genes with TEs inserted within 1 kb upstream of the transcription start site (−1 kb TSS; A), within 1 kb downstream of the transcription termination site (+1 kb TTS; B), in the gene body (C) or without TE insertions (D). For the three groups of genes with nearby TE insertions the distributions of gene expression (including only genes with FPKM > 0) are statistically different between the two genotypes (P < 0.01 by Kolmogorov-Smirnov test), with higher expression in rpd1/rmr6 mutants compared to B73 for expression values ranging from 0.5 to 10 FPKM (expression bins −1 to 3). On the contrary, genes without TE insertions (inside or nearby) show similar distribution of gene expression between genotypes. Graph (E) represents instead the average gene expression levels as a function of the distance to the nearest TE for both genotypes. Distance was binned into 500 bp windows and a distance of 0 indicates genes that contain a TE in their gene bodies. Standard errors are shown. (TIFF 1251 kb) [file 12870_2017_1108_MOESM8_ESM.tif]

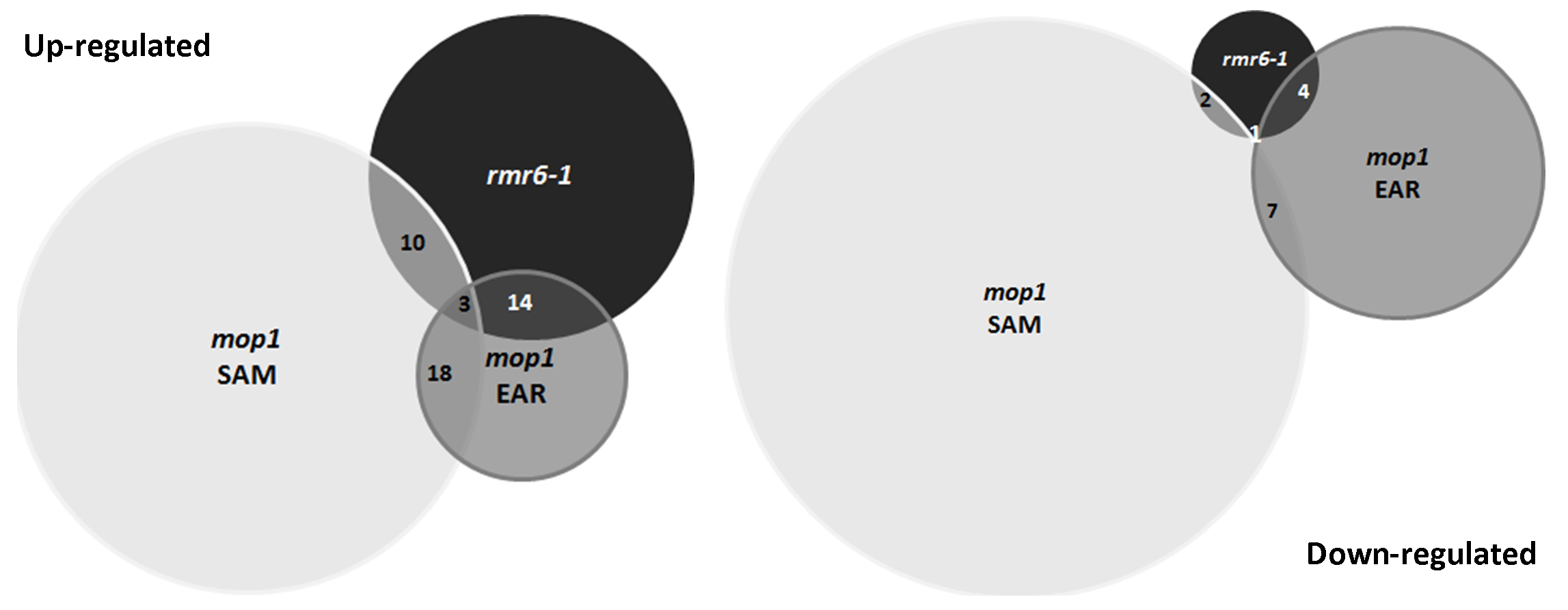

Supplement: Supplementary file 11 — Summary of genes resulting misregulated in both rpd1/rmr6 and rdr2/mop1 maize mutants. Venn diagrams representing the genes commonly up- and down-regulated in both maize RdDM mutants. Genes differentially expressed in rdr2/mop1 mutant were taken from [42, 43]. (TIFF 257 kb) [file 12870_2017_1108_MOESM11_ESM.tif]

Additional file 15

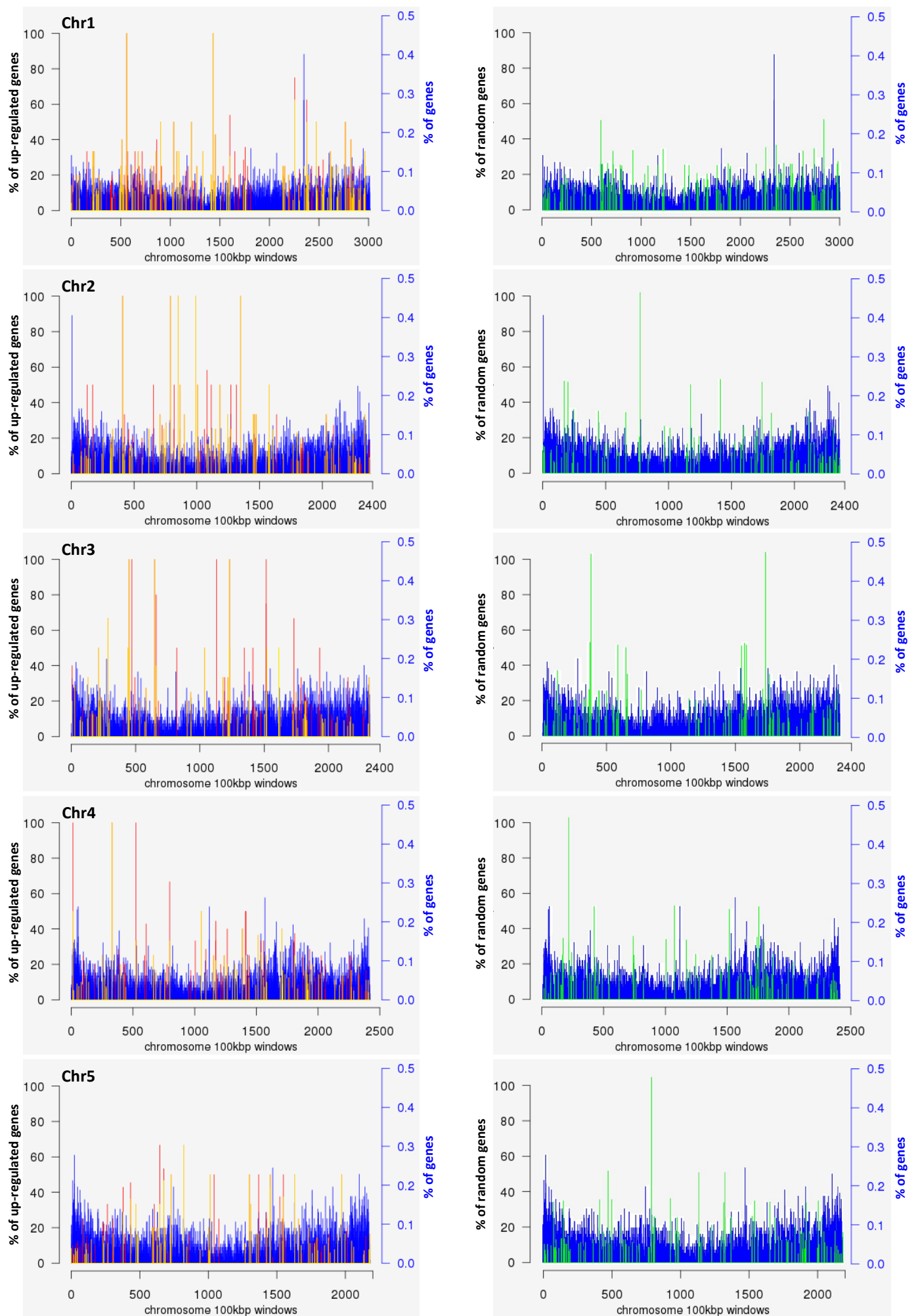

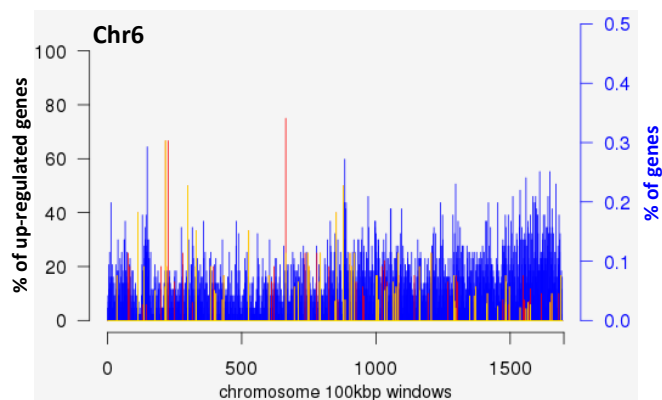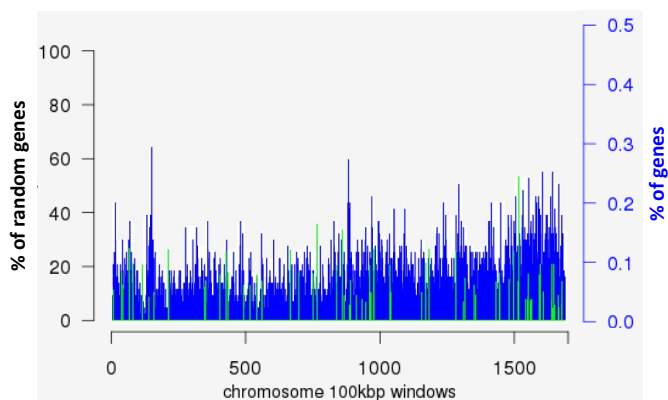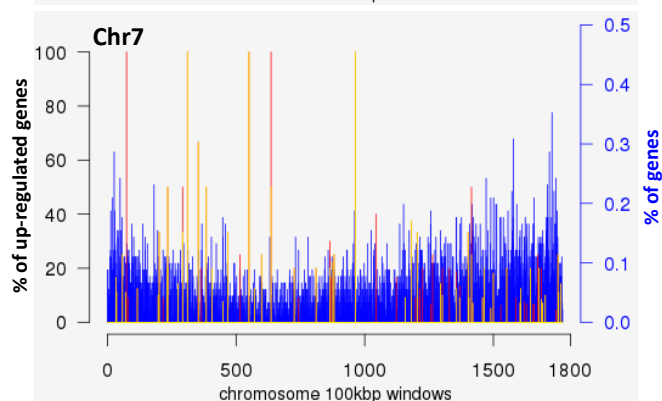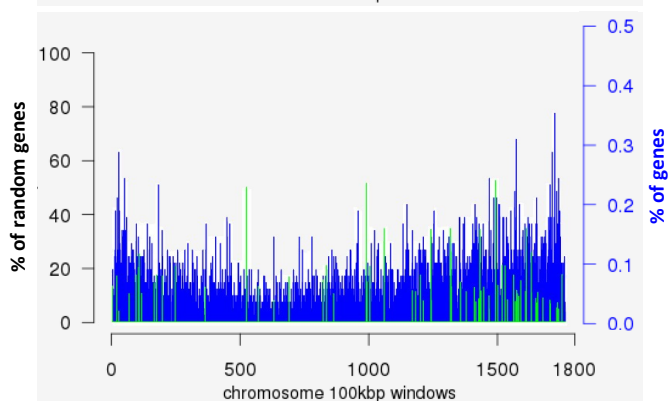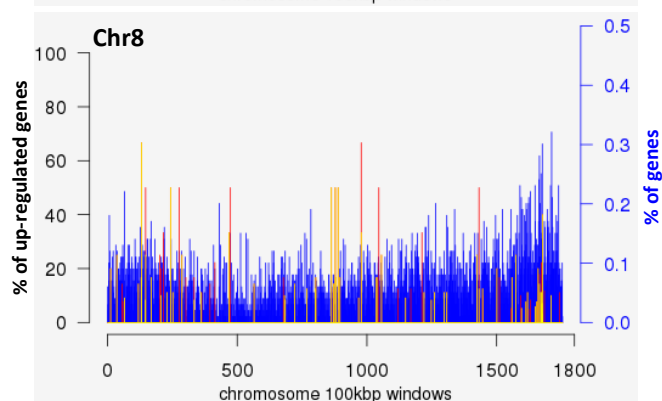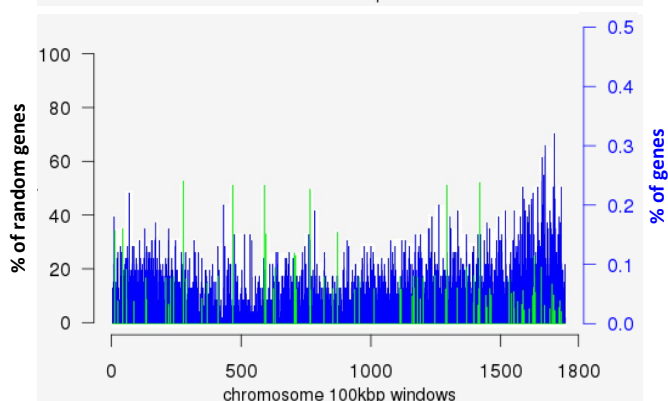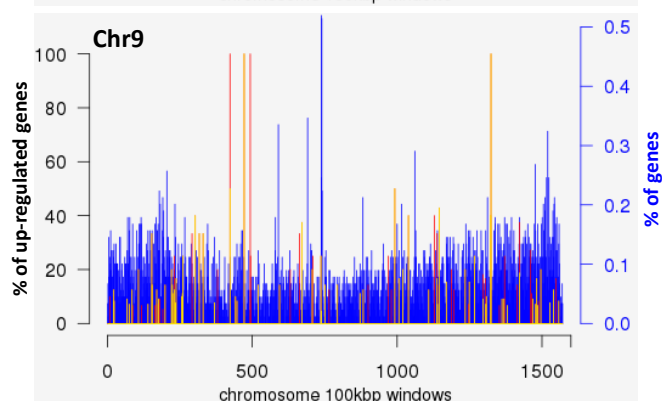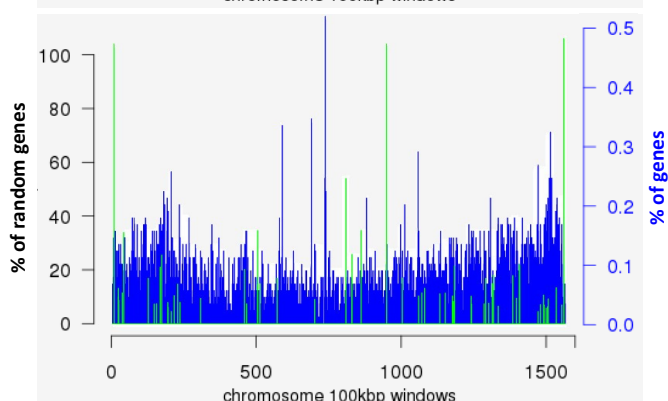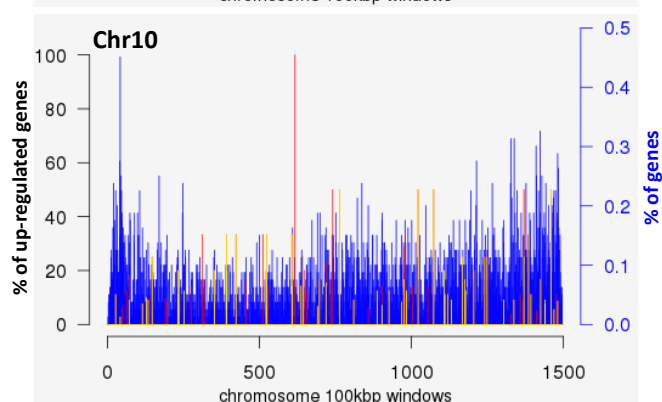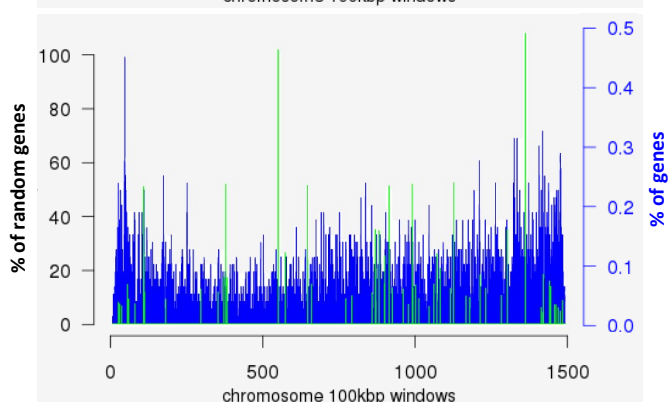

Supplement: Supplementary file 15 — Distribution plots Pol IV silenced loci along maize genome. The distribution plots of the differentially expressed genes along the ten maize chromosomes indicate the preferential co-localization of rpd1/rmr6 de-repressed genes (left plots). The chromosomes were divided in 100Kbp not-overlapping windows and for each window the percentage of genes (with respect to the total chromosome genes; blue bars) and of over-expressed genes (with respect to the window gene content) are reported. Yellow and red bars depict the window percentage of up-regulated genes shared in at least three or two independent comparisons, respectively. As control, the distribution of 880 genes randomly selected from the list of 40,457 expressed genes is reported as green bars (plots on the right). The two distributions resulted strongly statistically different (P = 1 × 10^−16^, by Wilcoxon test), with the random genes uniformly distributed along the genome (they resulted included in 876 genome 100 Kb windows versus the 737 including the up-regulated genes). (PDF 1141 kb) [file 12870_2017_1108_MOESM15_ESM.pdf]

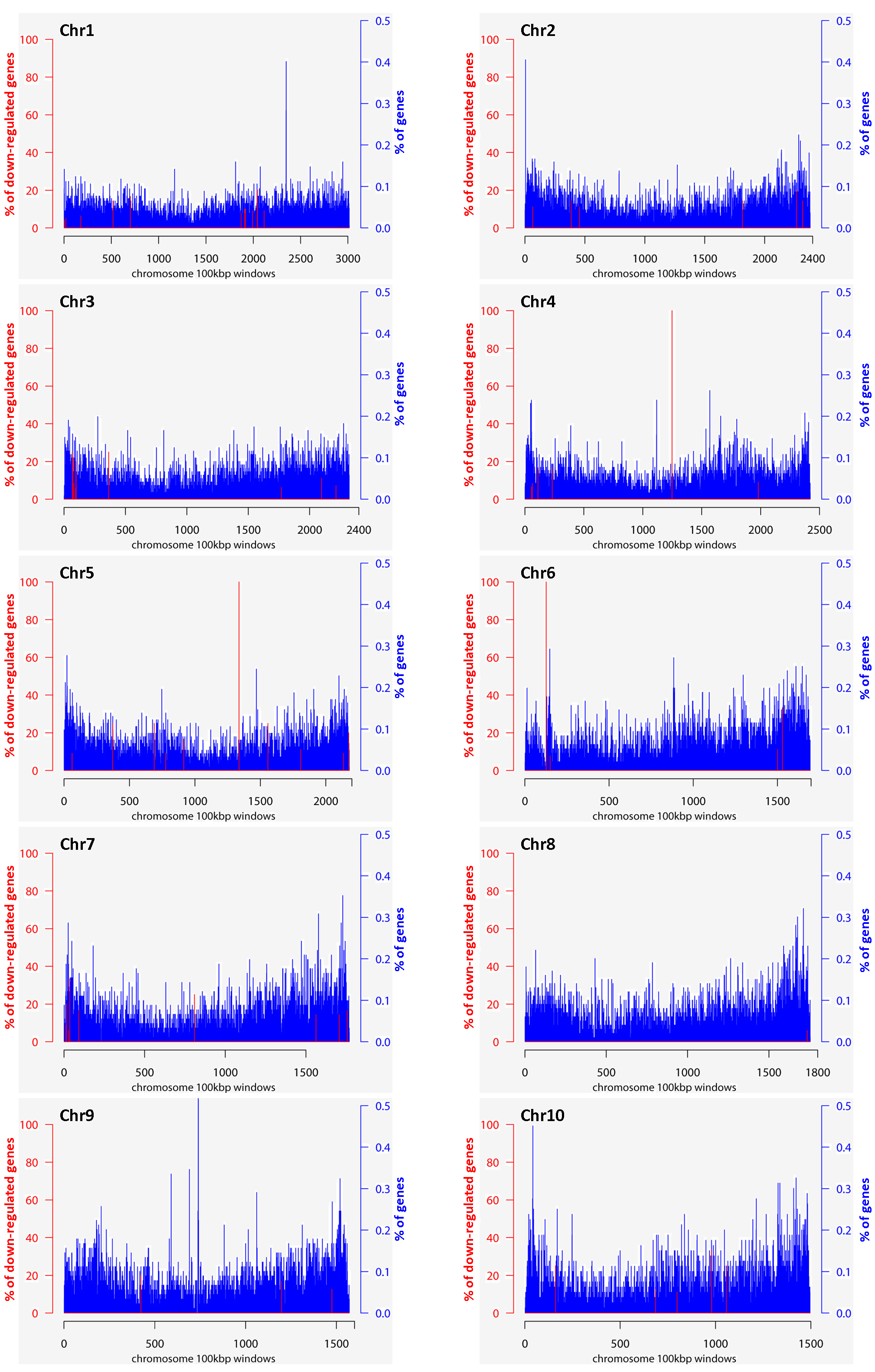

Supplement: Supplementary file 16 — Distribution plots along maize genome of genes down-regulated in rpd1/rmr6 mutant leaves. The distribution plots of the 71 rpd1/rmr6 down-regulated genes show they are included in 67 independent genome windows uniformly distributed along the maize chromosomes. The chromosomes were divided in 100Kbp not-overlapping windows and for each window the percentage of genes (with respect to the total chromosome genes; blue bars) and of down-regulate genes (with respect to the window gene content; red bars) are reported. (TIFF 2873 kb) [file 12870_2017_1108_MOESM16_ESM.tif]

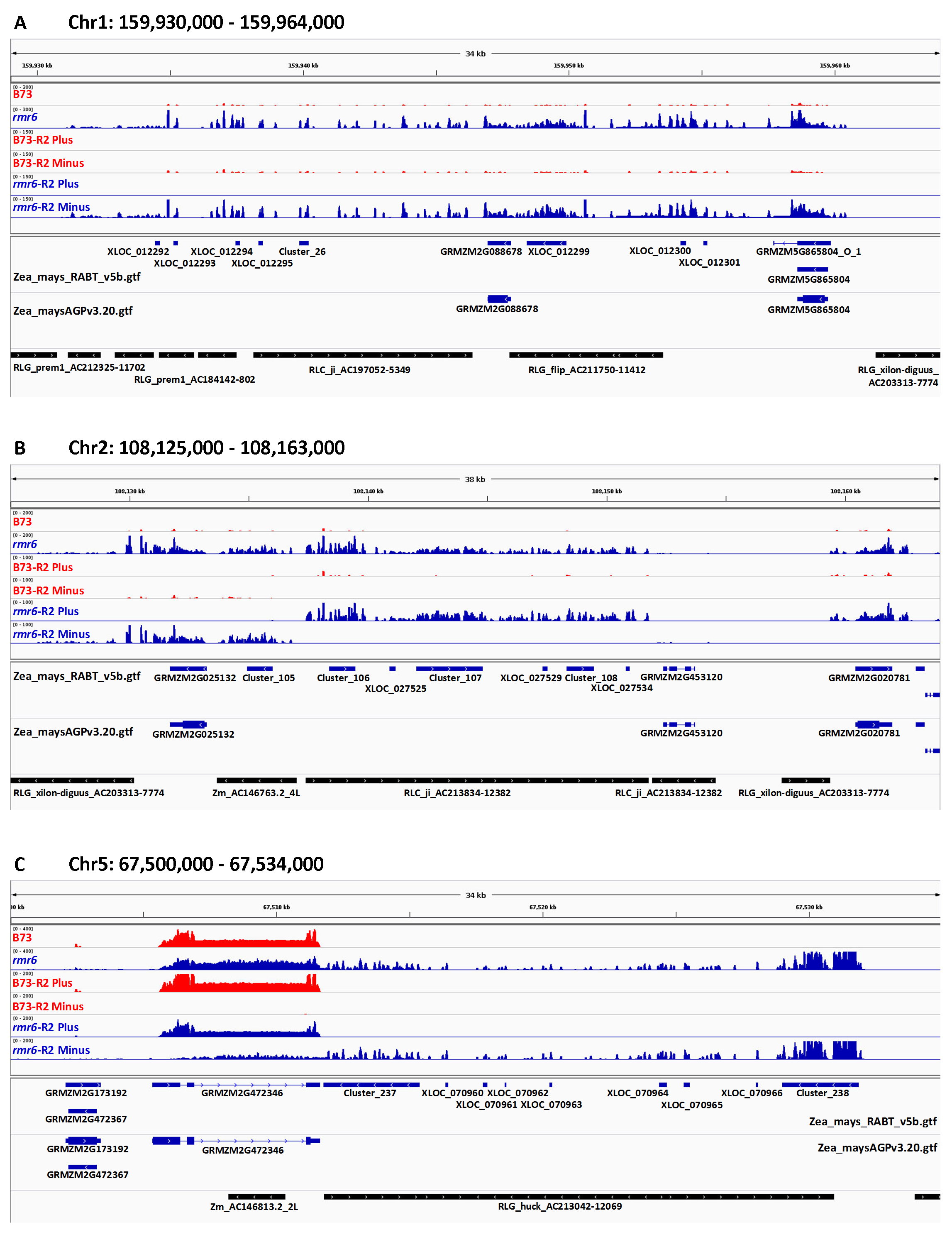

Supplement: Supplementary file 17 — Genome browser view of RNA-Seq reads mapped at rpd1/rmr6 misregulated gene cluster. Genome browser (IGV - Integrative Genomics Viewer; http://software.broadinstitute.org/software/igv/) views of B73 (red) and rpd1/rmr6–1 mutant (blue) RNA-Seq reads (normalized to the total of mapped reads) over three examples of large chromosomal regions de-repressed in rpd1/rmr6 mutant. Pol IV transcriptional release could interest one (A, C) or both strands (B) and interests several genes in TE-rich regions. Total mapped reads (replicates 1 and 2) and strand-specific mapped reads (replicate 2) are reported. (TIFF 1222 kb) [file 12870_2017_1108_MOESM17_ESM.tif]

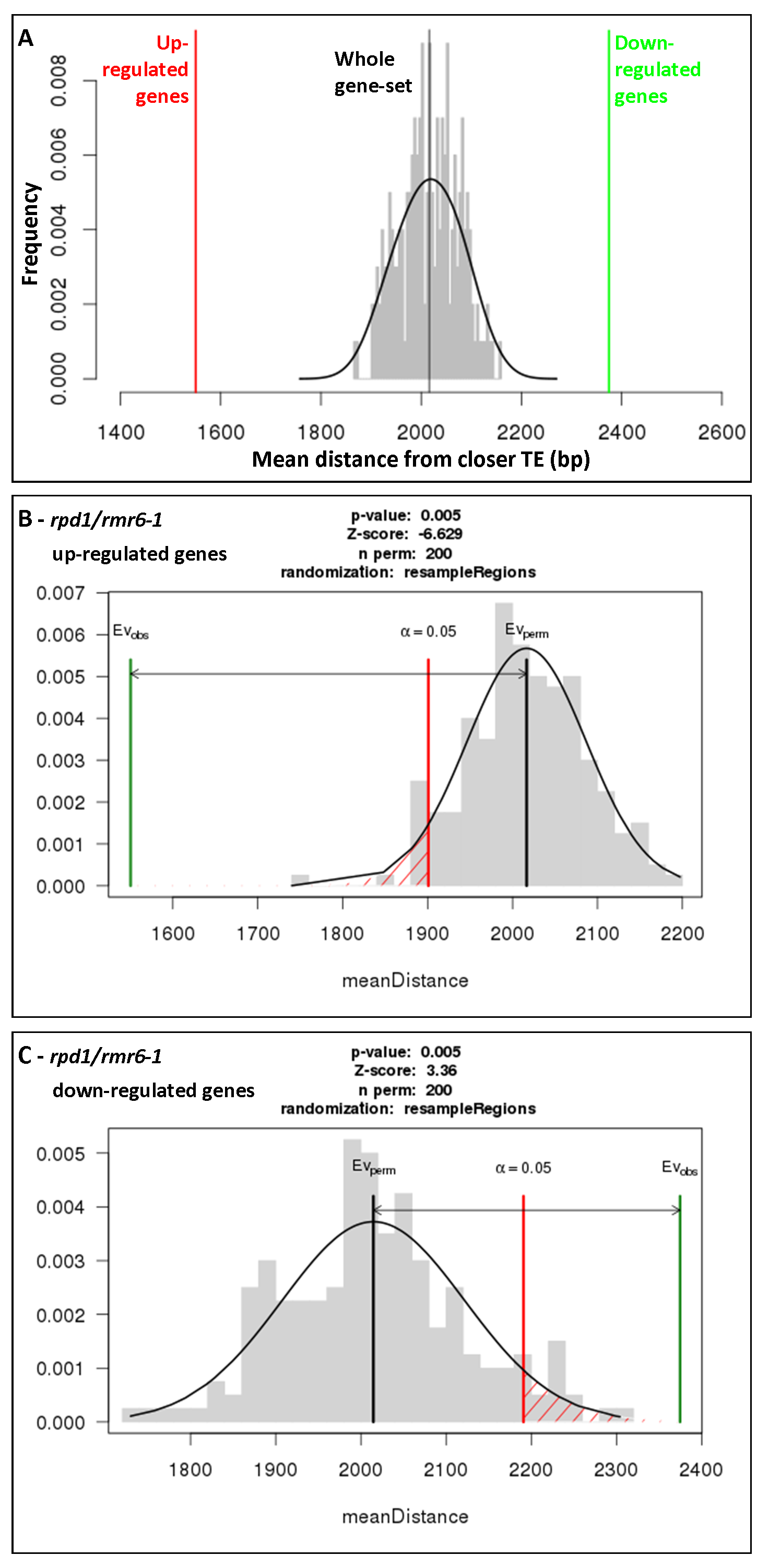

Supplement: Supplementary file 18 — Elaborated and raw results of permutation test on association between differentially expressed genes and TEs. Permutation test using the regioneR R package [100] revealed that up-regulated genes are significantly closer to TEs (average distance: 1550 bp) than the average of genes in the genome (permutation evaluated average distance: 2017 bp; P < 0.005), while down-regulated genes resulted more distant (2374 bp; P < 0.005). Graph in (A) was produced by combining the single graphs reported in (B) and (C), obtained from the independent analysis of up- and down-regulated genes. Differentially expressed genes for this analysis (log2FC > |1|, FDR < 0.05) were obtained with Cuffdiff starting from all the sequenced samples. (TIFF 600 kb) [file 12870_2017_1108_MOESM18_ESM.tif]

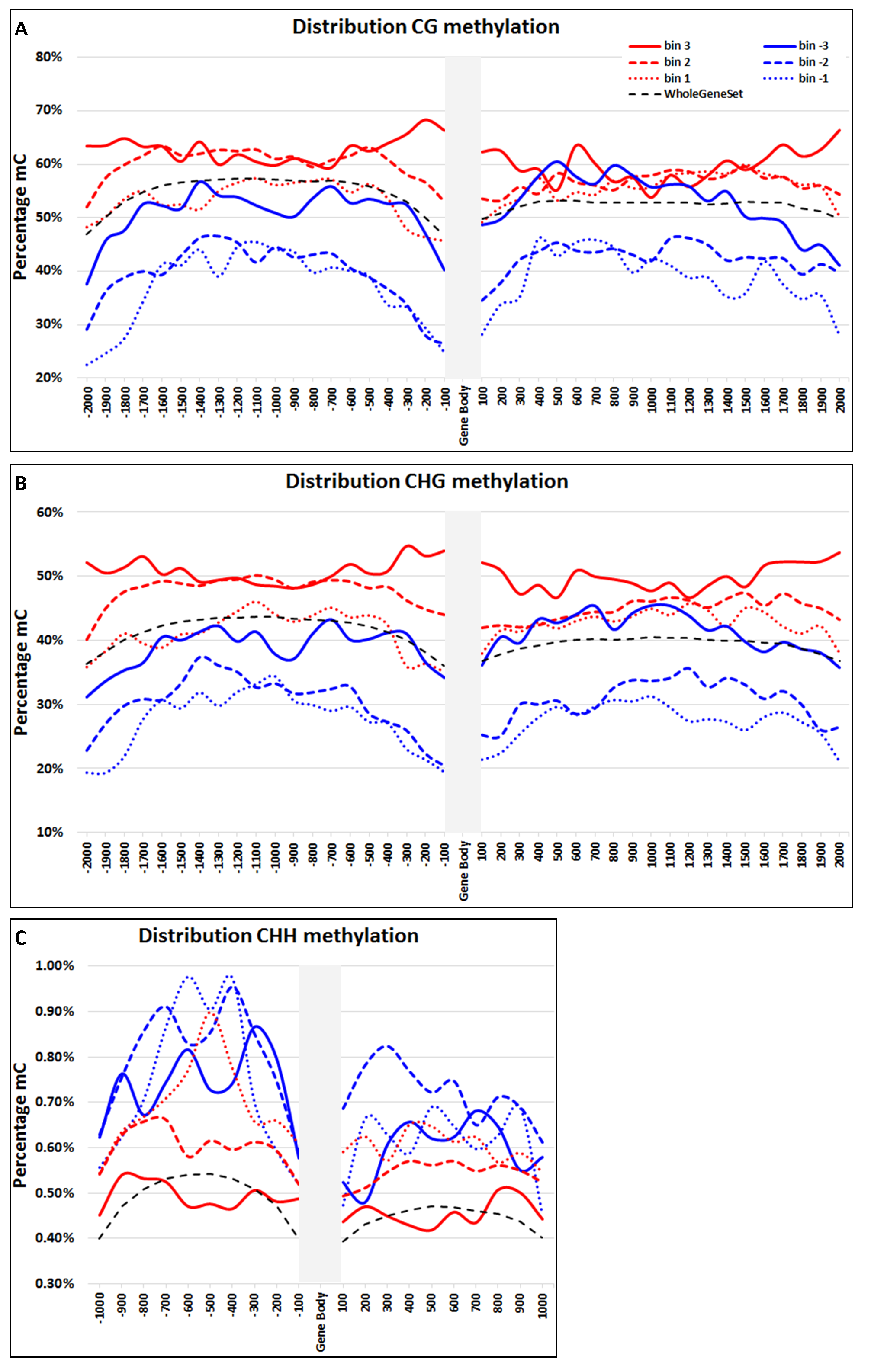

Supplement: Supplementary file 20 — DNA methylation profiles at differentially expressed gene flanking regions. Methylation levels in each context (CG, CHG and CHH) were computed for the flanking regions (2 Kb for CG and CHG, 1 Kb for CHH; see Methods) of differentially expressed genes. Genes were divided in bins according to fold change variation, and methylation levels of each bin were compared to the average of genes in the genome. (TIFF 1402 kb) [file 12870_2017_1108_MOESM20_ESM.tif]

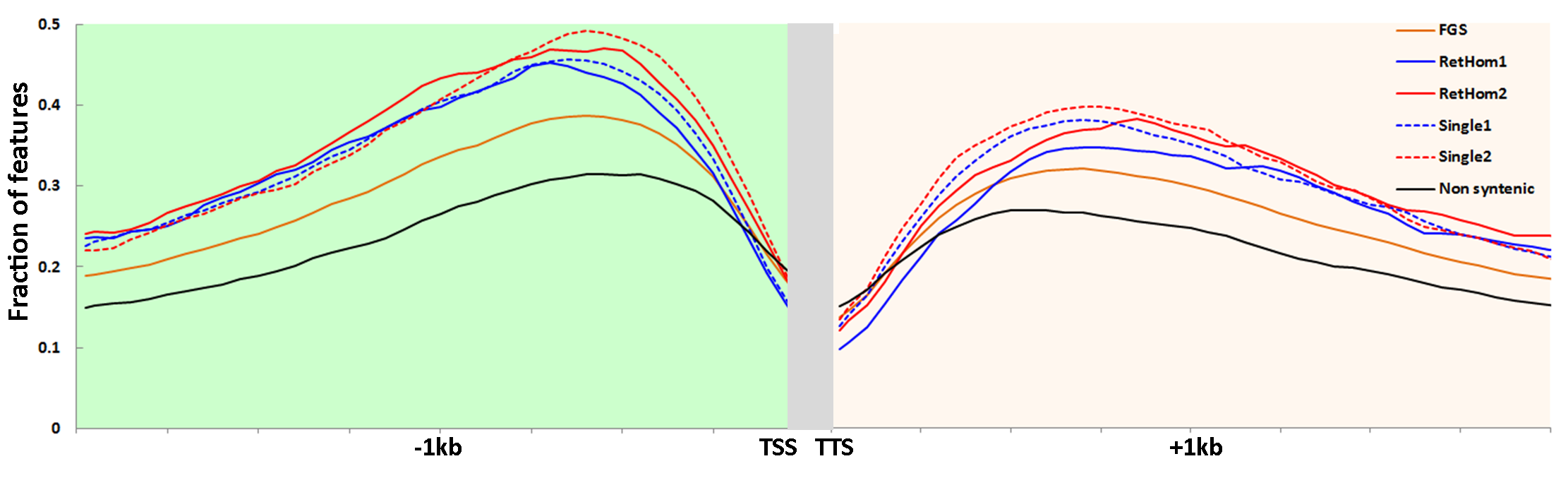

Supplement: Supplementary file 23 — Distribution plots of siRNA loci occupancy. The plots of siRNA loci coverage in flanking regions of subgenome genes, further split between homeologs and single copy genes of each subgenome, confirm that genes of the recessive subgenome 2 are preferentially siRNA-enriched in the upstream region, without differences between retained homeologs and single copy genes. (TIFF 395 kb) [file 12870_2017_1108_MOESM23_ESM.tif]
