# Supplementary material for: Maize RNA PolIV affects the expression of genes with nearby TE insertions and has a genome-wide repressive impact on transcription
Source: BMC Plant Biol. 2017 Oct 12;17:161. doi: 10.1186/s12870-017-1108-1 (PMC5639751; doi:10.1186/s12870-017-1108-1)
Supplement: Supplementary file 4 — Results of abundance filter on annotated genes and transcripts. The maize transcriptome annotation used in this study includes 160,488 transcripts at 114,382 loci [40] that were initially filtered based on the Cuffdiff test-status (see Methods) to exclude the not expressed or too lowly expressed genes/transcripts (roughly excluding all those with FPKM < 1 in all the analyzed samples). (DOCX 16 kb) [file 12870_2017_1108_MOESM4_ESM.docx]

**Additional file 4: Results of abundance filter on annotated genes and transcripts.**

|  | **Total** | **Expressed** | **Low data** |
| --- | --- | --- | --- |
| **Annotated Genes** | 114,382 | 40,457 | 73,925 |
| **Annotated Transcripts** | 160,488 | 66,153 | 94,335 |

The maize transcriptome annotation used in this study includes 160,488 transcripts at 114,382 loci (Forestan et al. 2016) that were initially filtered based on the Cuffdiff test-status (see Methods) to exclude the not expressed or too lowly expressed genes/transcripts (roughly excluding all those with FPKM<1 in all the analyzed samples).
