# Supplementary material for: Maize RNA PolIV affects the expression of genes with nearby TE insertions and has a genome-wide repressive impact on transcription
Source: BMC Plant Biol. 2017 Oct 12;17:161. doi: 10.1186/s12870-017-1108-1 (PMC5639751; doi:10.1186/s12870-017-1108-1)
Supplement: Supplementary file 5 — Results of abundance filter on transcripts with different coding potential. Abundance filter (based on the Cuffdiff test-status; see Methods) was applied to filter out the not expressed or too lowly expressed transcripts (roughly excluding transcripts with FPKM < 1 in all the analyzed samples). (DOCX 16 kb) [file 12870_2017_1108_MOESM5_ESM.docx]

**Additional file 5: Results of abundance filter on transcripts with different coding potential.**

| **Transcript Class** | **Coding** | **lncRNA** | **sRNA precursor** | **TE-related** | **Total** |
| --- | --- | --- | --- | --- | --- |
| **Expressed** | 53,654 | 4,898 | 7,292 | 309 | 66,153 |
| **Low data** | 70,683 | 8,489 | 14,332 | 831 | 94,335 |

Abundance filter (based on the Cuffdiff test-status; see Methods) was applied to filter out the not expressed or too lowly expressed transcripts (roughly excluding transcripts with FPKM<1 in all the analyzed samples).
