# Supplementary material for: Maize RNA PolIV affects the expression of genes with nearby TE insertions and has a genome-wide repressive impact on transcription
Source: BMC Plant Biol. 2017 Oct 12;17:161. doi: 10.1186/s12870-017-1108-1 (PMC5639751; doi:10.1186/s12870-017-1108-1)
Supplement: Supplementary file 9 — b1 gene overexpression in rpd1/rmr6 mutant leaves. The booster1 (b1, GRMZM2G172795) gene, which encodes a basic helix-loop-helix protein, resulted highly up-regulated in all the performed differential expression analyses, represents an hallmark of epigenetic silencing release in rpd1/rmr6 leaves compared with wild type. (DOCX 16 kb) [file 12870_2017_1108_MOESM9_ESM.docx]

**Additional file 9: *b1* gene overexpression in *rpd1/rmr6* mutant leaves.**

|  | **All-test-set** | **All-test-set - RSEM+EBSeq** | **Stress-test-set** | **Control-test-set** |
| --- | --- | --- | --- | --- |
| **log2FC *rmr6*/B73** | 2.70 | 3.12 | 1.55 | 3.06 |

The *booster1* (*b1*, GRMZM2G172795) gene, which encodes a basic helix-loop-helix protein, resulted highly up-regulated in all the performed differential expression analyses, represents an hallmark of epigenetic silencing release in *rpd1/rmr6* leaves compared with wild type.
