# Supplementary material for: Maize RNA PolIV affects the expression of genes with nearby TE insertions and has a genome-wide repressive impact on transcription
Source: BMC Plant Biol. 2017 Oct 12;17:161. doi: 10.1186/s12870-017-1108-1 (PMC5639751; doi:10.1186/s12870-017-1108-1)
Supplement: Supplementary file 12 — List and annotation of genes resulting as misregulated in both rpd1/rmr6 and rdr2/mop1 maize mutants. (DOCX 19 kb) [file 12870_2017_1108_MOESM12_ESM.docx]

**Additional file 12: List and annotation of genes resulting as misregulated in both *rpd1/rmr6-1* and *rdr2/mop1-1* maize mutants.**

| **ID** | **Description** |  |
| --- | --- | --- |
| GRMZM2G077256 | Ribosomal protein | Upregulated in *rmr6-1*, *mop1-1* SAM, *mop1-1* EAR |
| GRMZM2G479245 | MFP1 attachment factor 1 | Upregulated in *rmr6-1*, *mop1-1* SAM, *mop1-1* EAR |
| GRMZM2G028218 | DnaJ chaperonin | Upregulated in *rmr6-1*, *mop1-1* SAM, *mop1-1* EAR |
| GRMZM2G012160 | Cystatin2 | Upregulated in *rmr6-1*, *mop1-1* SAM |
| GRMZM2G032339 | ZmMADS4 | Upregulated in *rmr6-1*, *mop1-1* SAM |
| GRMZM2G063244 | Peptidyl-prolyl cis-trans isomerase | Upregulated in *rmr6-1*, *mop1-1* SAM |
| GRMZM2G300238 | Uncharacterized protein | Upregulated in *rmr6-1*, *mop1-1* SAM |
| GRMZM2G324221 | Uncharacterized protein | Upregulated in *rmr6-1*, *mop1-1* SAM |
| GRMZM2G392513 | myo-inositol-1-phosphate synthase | Upregulated in *rmr6-1*, *mop1-1* SAM |
| GRMZM2G400655 | C2H2 zinc-finger protein SERRATE | Upregulated in *rmr6-1*, *mop1-1* SAM |
| GRMZM2G703755 | Uncharacterized protein | Upregulated in *rmr6-1*, *mop1-1* SAM |
| GRMZM2G157505 | EGG APPARATUS-1 protein | Upregulated in *rmr6-1*, *mop1-1* SAM |
| GRMZM2G429982 | Osmotin-like protein | Upregulated in *rmr6-1*, *mop1-1* SAM |
| AC201780.3_FG003 | Uncharacterized protein | Upregulated in *rmr6-1*, *mop1-1* EAR |
| AC203863.3_FG007 | Uncharacterized protein | Upregulated in *rmr6-1*, *mop1-1* EAR |
| GRMZM2G010636 | Uncharacterized protein | Upregulated in *rmr6-1*, *mop1-1* EAR |
| GRMZM2G041619 | Uncharacterized protein | Upregulated in *rmr6-1*, *mop1-1* EAR |
| GRMZM2G047105 | Uncharacterized protein | Upregulated in *rmr6-1*, *mop1-1* EAR |
| GRMZM2G067842 | Uncharacterized protein | Upregulated in *rmr6-1*, *mop1-1* EAR |
| GRMZM2G088413 | Uncharacterized protein | Upregulated in *rmr6-1*, *mop1-1* EAR |
| GRMZM2G133514 | Uncharacterized protein | Upregulated in *rmr6-1*, *mop1-1* EAR |
| GRMZM2G333361 | Uncharacterized protein | Upregulated in *rmr6-1*, *mop1-1* EAR |
| GRMZM2G360389 | Putative SET-domain containing protein family | Upregulated in *rmr6-1*, *mop1-1* EAR |
| GRMZM2G411216 | Uncharacterized protein | Upregulated in *rmr6-1*, *mop1-1* EAR |
| GRMZM2G456241 | Putative RING zinc finger domain superfamily protein | Upregulated in *rmr6-1*, *mop1-1* EAR |
| GRMZM5G859350 | Uncharacterized protein | Upregulated in *rmr6-1*, *mop1-1* EAR |
| GRMZM5G866269 | Uncharacterized protein | Upregulated in *rmr6-1*, *mop1-1* EAR |
| GRMZM2G131756 | Demeter-like 1 | Downregulated in *rmr6-1*, *mop1-1* SAM, *mop1-1* EAR |
| GRMZM2G324973 | Uncharacterized protein | Downregulated in *rmr6-1*, *mop1-1* SAM |
| GRMZM2G449123 | Pumilio 5 | Downregulated in *rmr6-1*, *mop1-1* SAM |
| GRMZM2G099820 | Uncharacterized protein | Downregulated in *rmr6-1*, *mop1-1* EAR |
| GRMZM2G117281 | Uncharacterized protein | Downregulated in *rmr6-1*, *mop1-1* EAR |
| GRMZM2G433731 | Uncharacterized protein | Downregulated in *rmr6-1*, *mop1-1* EAR |
| GRMZM5G828987 | Uncharacterized protein | Downregulated in *rmr6-1*, *mop1-1* EAR |
