# Supplementary material for: Maize RNA PolIV affects the expression of genes with nearby TE insertions and has a genome-wide repressive impact on transcription
Source: BMC Plant Biol. 2017 Oct 12;17:161. doi: 10.1186/s12870-017-1108-1 (PMC5639751; doi:10.1186/s12870-017-1108-1)
Supplement: Supplementary file 13 — TE superfamilies identified amongst genes differentially expressed in rpd1/rmr6 mutant. The number and relative percentage of up- and down-regulated genes classified as TEs (loci with at least one transcript previously classified as HC-TE or pr-TE, see Methods) categorized in superfamilies. The relative abundance of each TEs superfamily within the 114,382 annotated genes is also reported. (DOCX 18 kb) [file 12870_2017_1108_MOESM13_ESM.docx]

**Additional file 13: TE superfamilies identified amongst genes differentially expressed in *rpd1/rmr6* mutant.**

| **TE FAMILY** | **% of Annotated Genes** | ***rpd1/rmr6* Up-regulated** | | ***rpd1/rmr6* Down-regulated** | |
| --- | --- | --- | --- | --- | --- |
| **RLG-Gypsy** | 11.6% | 61 | 34.7% | 2 | 18.2% |
| **RLC-Copia** | 6.8% | 61 | 34.7% | 0 | 0% |
| **RLX-Unknown LTR** | 6.2% | 15 | 8.5% | 1 | 9.1% |
| **DTA-hAT** | 11.5% | 6 | 3.4% | 1 | 9.1% |
| **DTM-Mutator** | 11.8% | 7 | 3.4% | 1 | 9.1% |
| **DTC-CACTA** | 33.4% | 15 | 8.5% | 4 | 36.3% |
| **DTH-PIF/Harbinger** | 7.8% | 7 | 4.0% | 0 | 0% |
| **RST-tRNA** | 0.3% | 1 | 0.6% | 0 | 0% |
| **DTT-Tc1/Mariner** | 0.8% | 1 | 0.6% | 1 | 9.1% |
| **RIL-L1** | 4.2% | 1 | 0.6% | 0 | 0% |
| **DHH-Helitron** | 5.1% | 1 | 0.6% | 1 | 9.1% |
| **RIT-RTE** | 0.5% | 1 | 0.6% | 0 | 0% |
| **Total TEs (**18,545) | 16.2% | **177** | 20.0% | **11** | 15.5% |
| **Genes (**114,382**)** |  | 880 |  | 71 |  |

The number and relative percentage of up- and down-regulated genes classified as TEs (loci with at least one transcript previously classified as HC-TE or pr-TE, see Materials and Methods) categorized in superfamilies. The relative abundance of each TEs superfamily within the 114,382 annotated genes is also reported.
