# Supplementary material for: Maize RNA PolIV affects the expression of genes with nearby TE insertions and has a genome-wide repressive impact on transcription
Source: BMC Plant Biol. 2017 Oct 12;17:161. doi: 10.1186/s12870-017-1108-1 (PMC5639751; doi:10.1186/s12870-017-1108-1)
Supplement: Supplementary file 14 — TE families differentially transcribed in rpd1/rmr6 mutant. Differentially expressed TE families (log2FC > |2|; FDR < 0.01) identified by edgeR; for class I TEs the number of full-length elements and partial fragments were obtained from [31]. (DOCX 22 kb) [file 12870_2017_1108_MOESM14_ESM.docx]

**Additional file 14: TE families differentially transcribed in rpd1/rmr6 mutant.**

| **TE** | **Counts B73** | **Counts rmr6** | **log2FC** | **FDR** | **# FL** | **# fragments** |
| --- | --- | --- | --- | --- | --- | --- |
| RLG_ahoru_AC187284_1845 | 1828 | 364485 | 7.58 | 0.0E+00 | 1 | 385 |
| RLC_gekog_AC203828_7984 | 33 | 6344 | 7.52 | 1.1E-243 | 2 | 203 |
| RLG_aneas_AC203312_7773 | 9 | 1624 | 7.40 | 3.2E-149 | 2 | 40 |
| DTA_ZM00070_consensus | 0 | 27 | 6.74 | 9.9E-08 |  |  |
| RLG_amiin_AC191769_3319 | 11 | 1249 | 6.74 | 2.9E-132 | 4 | 95 |
| RLG_gati_AC195589_4669 | 36 | 2274 | 5.92 | 6.6E-154 | 1 | 103 |
| RLX_uvis_AC197719_164 | 25 | 1523 | 5.86 | 1.7E-129 | 1 | 55 |
| DTM_Zm33213_AC206876_1 | 2 | 59 | 4.67 | 1.0E-12 |  |  |
| RLG_wyly_AC198779_6150 | 14 | 349 | 4.56 | 8.1E-50 | 2 | 24 |
| DTM_Zm25996_AC205363_1 | 31 | 702 | 4.43 | 3.6E-76 |  |  |
| RLG_ruugu_AC214105_12477 | 13 | 294 | 4.42 | 1.2E-42 | 1 | 107 |
| DTM_Zm23554_AC210619_1 | 29 | 630 | 4.37 | 1.6E-67 |  |  |
| RLX_poarow_AC200533_6899 | 10 | 198 | 4.22 | 1.2E-32 | 1 | 32 |
| DTM_Zm08014_AC190710_1 | 25 | 467 | 4.15 | 1.6E-56 |  |  |
| DTM_Zm11868_AC202888_1 | 329 | 5007 | 3.87 | 3.3E-117 |  |  |
| RLX_vufe_AC194263_159 | 13 | 199 | 3.86 | 6.1E-29 | 1 | 36 |
| DTM_Zm09664_AC195786_1 | 257 | 3796 | 3.83 | 3.3E-116 |  |  |
| RLC_huti_AC197153_5449 | 11 | 157 | 3.75 | 1.1E-29 | 2 | 208 |
| RLC_ajajog_AC191578_3186 | 8 | 113 | 3.72 | 3.4E-18 | 1 | 64 |
| RLG_rimaar_AC197236_5493 | 13 | 179 | 3.70 | 2.2E-27 | 1 | 31 |
| DTM_Zm09546_AC191082_1 | 24 | 326 | 3.69 | 5.7E-38 |  |  |
| RLC_debeh_AC177840_246 | 842 | 11244 | 3.68 | 6.6E-122 | 27 | 2050 |
| RLC_eninu_AC191055_2893 | 21968 | 282914 | 3.63 | 1.6E-128 | 64 | 1084 |
| DTA_ZM00020_consensus | 88 | 984 | 3.42 | 3.2E-61 |  |  |
| RLX_wuywu_AC190718_2536 | 3 | 34 | 3.34 | 8.3E-06 | 1 | 22 |
| DTM_Zm20615_AC216184_1 | 21 | 197 | 3.16 | 2.4E-24 |  |  |
| DTM_Zm33205_AC203276_1 | 31 | 267 | 3.04 | 4.4E-30 |  |  |
| RLG_uwum_AC190887_2701 | 1559 | 11116 | 2.78 | 1.1E-80 | 238 | 13271 |
| DTT_ZM00002_consensus | 25 | 166 | 2.66 | 1.4E-16 |  |  |
| RLC_gilovu_AC196048_4919 | 4 | 26 | 2.57 | 6.0E-04 | 2 | 82 |
| RLC_sofi_AC215261_13035 | 663 | 3909 | 2.50 | 4.9E-61 | 6 | 427 |
| DTC_ZM00082_consensus | 145 | 813 | 2.43 | 5.5E-39 |  |  |
| DTH_ZM00240_consensus | 20 | 112 | 2.42 | 2.5E-11 |  |  |
| RLG_pagof_AC198204_5908 | 21 | 116 | 2.40 | 3.9E-13 | 8 | 29 |
| DTA_ZM00039_consensus | 97 | 531 | 2.39 | 1.5E-28 |  |  |
| RLG_ulik_AC205416_8790 | 204 | 1104 | 2.38 | 1.2E-40 | 2 | 109 |
| DTM_Zm33222_AC190995_1 | 5 | 28 | 2.37 | 3.7E-04 |  |  |
| DTM_Zm00884_AC214130_1 | 346 | 1711 | 2.25 | 1.4E-46 |  |  |
| DTA_ZM00240_consensus | 16 | 77 | 2.19 | 6.3E-08 |  |  |
| RLC_fuvej_AC194215_3955 | 21 | 97 | 2.14 | 3.8E-08 | 2 | 55 |
| DTA_ZM00090_consensus | 20 | 89 | 2.08 | 3.3E-08 |  |  |
| RLC_stonor_AC212476_11849 | 5764 | 25378 | 2.08 | 1.0E-46 | 27 | 1453 |
| RLX_vegu_AC190718_85 | 8035 | 34520 | 2.05 | 1.2E-47 | 1 | 2113 |
| RLC_ijiret_AC211361_11134 | 101 | 26 | -2.00 | 3.8E-08 | 4 | 32 |
| RLX_sari_AC184117_11 | 640 | 161 | -2.05 | 5.0E-22 | 5 | 176 |
| DTC_ZM00066_consensus | 85 | 21 | -2.06 | 1.3E-05 |  |  |
| DTC_ZM00077_consensus | 3718 | 925 | -2.06 | 3.6E-43 |  |  |
| DTC_ZM00016_consensus | 1573 | 381 | -2.10 | 1.6E-38 |  |  |
| DTC_ZM00009_consensus | 157 | 37 | -2.13 | 2.3E-13 |  |  |
| RLX_teki_AC202867_7492 | 4608 | 932 | -2.36 | 3.5E-47 | 1 | 139 |
| RLX_sela_AC195130_4415 | 156 | 29 | -2.47 | 7.1E-15 | 1 | 301 |
| RLX_osed_AC191084_2931 | 34 | 6 | -2.51 | 1.1E-04 | 2 | 1052 |
| RLG_ewog_AC212715_11905 | 290 | 42 | -2.84 | 1.6E-27 | 1 | 107 |
| DTA_ZM00195_consensus | 36 | 5 | -2.84 | 4.3E-05 |  |  |
| RLG_nobe_AC198224_5924 | 563 | 61 | -3.26 | 1.1E-47 | 1 | 49 |
| RLG_boja_AC200053_6723 | 548 | 4 | -7.07 | 2.1E-88 | 1 | 17 |

Differentially expressed TE families (log2FC >|2|; FDR< 0.01) identified by edgeR; for class I TEs the number of full-length elements and partial fragments were obtained from (Baucom et al. 2009)
