# Supplementary material for: Maize RNA PolIV affects the expression of genes with nearby TE insertions and has a genome-wide repressive impact on transcription
Source: BMC Plant Biol. 2017 Oct 12;17:161. doi: 10.1186/s12870-017-1108-1 (PMC5639751; doi:10.1186/s12870-017-1108-1)
Supplement: Supplementary file 21 — Summary of methylation levels at differentially expressed genes flanking regions obtained by permutation analysis. Methylation levels in each context (CG, CHG and CHH) were computed independently for the flanking regions (2 Kb for CG and CHG, 1 Kb for CHH; see Methods) of differentially expressed genes and compared to the average of genes in the genome using regioneR permutation approach [100]. Genes were divided into bins accordingly to fold change expression variation in rpd1/rmr6 mutant compared to wild-type (Additional file 19). For each bin the average methylation value is associated to the p-value obtained by permutation analysis, value that summarizes the statistical significance divergence between bin average methylation level and the average of annotated genes in the genome. Red and blue values indicate statistically higher and lower methylation levels, respectively, compared to the whole gene set. (DOCX 19 kb) [file 12870_2017_1108_MOESM21_ESM.docx]

**Additional file 21: Summary of methylation levels at differentially expressed genes flanking regions obtained by permutation analysis**

| **Upstream Region** | | **Bin average methylation levels and significance of variation compared to the full set of genes** | | | | | | | | | | | |
| --- | --- | --- | --- | --- | --- | --- | --- | --- | --- | --- | --- | --- | --- |
| **Methylation context** | **Whole Gene Set** | **DOWN-Reg -3** | **p-val** | **DOWN-Reg -2** | **p-val** | **DOWN-Reg -1** | **p-val** | **UP-Reg 1** | **p-val** | **UP-Reg 2** | **p-val** | **UP-Reg 3** | **p-val** |
| **CG** | **0.487** | 0.504 | ns | 0.382 | <0.005 | 0.355 | <0.005 | 0.531 | <0.01 | 0.598 | <0.005 | 0.631 | <0.005 |
| **CHG** | **0.344** | 0.375 | ns | 0.299 | <0.005 | 0.269 | <0.005 | 0.409 | <0.005 | 0.476 | <0.005 | 0.511 | <0.005 |
| **CHH** | **0.0398** | 0.0739 | <0.005 | 0.0798 | <0.005 | 0.0721 | <0.005 | 0.0689 | <0.005 | 0.0601 | <0.005 | 0.0493 | <0.005 |
| **Downstream Region** | | **Bin average methylation levels and significance of variation compared to the full set of genes** | | | | | | | | | | | |
| **Methylation context** | **Whole Gene Set** | **DOWN-Reg -3** | **p-val** | **DOWN-Reg -2** | **p-val** | **DOWN-Reg -1** | **p-val** | **UP-Reg 1** | **p-val** | **UP-Reg 2** | **p-val** | **UP-Reg 3** | **p-val** |
| **CG** | **0.489** | 0.529 | ns | 0.424 | <0.005 | 0.381 | <0.005 | 0.559 | <0.005 | 0.567 | <0.005 | 0.599 | <0.005 |
| **CHG** | **0.342** | 0.414 | <0.005 | 0.307 | <0.005 | 0.272 | <0.005 | 0.428 | <0.005 | 0.431 | <0.005 | 0.498 | <0.005 |
| **CHH** | **0.0399** | 0.0597 | <0.005 | 0.0722 | <0.005 | 0.0609 | <0.005 | 0.0602 | <0.005 | 0.0545 | <0.005 | 0.0455 | <0.005 |

Methylation levels in each context (CG, CHG and CHH) were computed independently for the flanking regions (2 Kb for CG and CHG, 1 Kb for CHH; see Materials and Methods) of differentially expressed genes and compared to the average of genes in the genome using regioneR permutation approach (Gel et al. 2016). Genes were divided into bins accordingly to fold change expression variation in *rpd1/rmr6* mutant compared to wild-type (Additional file 15). For each bin the average methylation value is associated to the p-value obtained by permutation analysis, value that summarizes the statistical significance divergence between bin average methylation level and the average of annotated genes in the genome. Red and blue values indicate statistically higher and lower methylation levels, respectively, compared to the whole gene set.
