# Supplementary material for: Maize RNA PolIV affects the expression of genes with nearby TE insertions and has a genome-wide repressive impact on transcription
Source: BMC Plant Biol. 2017 Oct 12;17:161. doi: 10.1186/s12870-017-1108-1 (PMC5639751; doi:10.1186/s12870-017-1108-1)
Supplement: Supplementary file 22 — Expression of subgenome 1 and subgenome 2 assigned genes in rpd1/rmr6 mutant and B73 wild-type plants. Differentially expressed gene models (log2FC > |1|, FDR < 0.05) ascribable to singletons and duplicates in the maize subgenomes or non-syntenic genes were obtained with Cuffdiff starting from all the sequenced samples. (DOCX 17 kb) [file 12870_2017_1108_MOESM22_ESM.docx]

**Additional file 22: Expression of subgenome 1 and subgenome 2 assigned genes in rpd1/rmr6 mutant and B73 wild-type plants**

| **Gene List** | **Single Sub1** | **RetHom Sub1** | **RetHom Sub2** | **Single Sub2** | **Non- Synt** | **Total** | **Ratio**  **2/1** | **Ratio Retained 2/1s** | **Ratio non-syntenic/ Subgenomes** |
| --- | --- | --- | --- | --- | --- | --- | --- | --- | --- |
| **FGS** | 8,962 | 3,228 | 3,228 | 3,947 | 20,291 | 39,656 | 0.59 | 1 | 1.05 |
| **FGS Expression Filtered** | 7,031 | 2,705 | 2,681 | 3,060 | 8,448 | 23,895 | 0.59 | 0.99 | 0.55 |
| **rmr6 Up** | 106 | 21 | 38 | 33 | 407 | 605 | 0.559 | 1.81 | 2.05 |
| **rmr6 Down** | 188 | 62 | 49 | 48 | 254 | 601 | 0.388 | 0.79 | 0.73 |

Differentially expressed gene models (log2FC>|1|, FDR< 0.05) ascribable to singletons and duplicates in the maize subgenomes or non-syntenic genes were obtained with Cuffdiff starting from all the sequenced samples.
